# Enzymatic spiroketal formation via oxidative rearrangement of pentangular polyketides

**DOI:** 10.1038/s41467-021-21432-9

**Published:** 2021-03-04

**Authors:** Britta Frensch, Thorsten Lechtenberg, Michel Kather, Zeynep Yunt, Martin Betschart, Bernd Kammerer, Steffen Lüdeke, Michael Müller, Jörn Piel, Robin Teufel

**Affiliations:** 1 Faculty of Biology, University of Freiburg, Schänzlestrasse 1, 79104 Freiburg, Germany; 2 BIOSS Center for Biological Signaling Studies, University of Freiburg, 79104 Freiburg, Germany; 3 Department of Molecular Biology and Genetics, Koç University, Istanbul, 34450 Turkey; 4 Institute of Pharmaceutical Sciences, University of Freiburg, 79104 Freiburg, Germany; 5 Hermann Staudinger Graduate School, University of Freiburg, 79104 Freiburg, Germany; 6 Institute of Microbiology, Eidgenössische Technische Hochschule (ETH) Zürich, 8093 Zürich, Switzerland

**Keywords:** Enzyme mechanisms, Biosynthesis, Natural products, Natural product synthesis

## Abstract

The structural complexity and bioactivity of natural products often depend on enzymatic redox tailoring steps. This is exemplified by the generation of the bisbenzannulated [5,6]-spiroketal pharmacophore in the bacterial rubromycin family of aromatic polyketides, which exhibit a wide array of bioactivities such as the inhibition of HIV reverse transcriptase or DNA helicase. Here we elucidate the complex flavoenzyme-driven formation of the rubromycin pharmacophore that is markedly distinct from conventional (bio)synthetic strategies for spiroketal formation. Accordingly, a polycyclic aromatic precursor undergoes extensive enzymatic oxidative rearrangement catalyzed by two flavoprotein monooxygenases and a flavoprotein oxidase that ultimately results in a drastic distortion of the carbon skeleton. The one-pot in vitro reconstitution of the key enzymatic steps as well as the comprehensive characterization of reactive intermediates allow to unravel the intricate underlying reactions, during which four carbon-carbon bonds are broken and two CO_2_ become eliminated. This work provides detailed insight into perplexing redox tailoring enzymology that sets the stage for the (chemo)enzymatic production and bioengineering of bioactive spiroketal-containing polyketides.

## Abstract (web summary)

Rubromycin family of natural products belongs to aromatic polyketides with diverse bioactivities, but details of their biosynthesis are limited. Here, the authors report the complete in vitro reconstitution of enzymatic formation of the spiroketal moiety of rubromycin polyketides, driven by flavin-dependent enzymes, and characterize reaction intermediates.

## Introduction

**Fig. 1:**
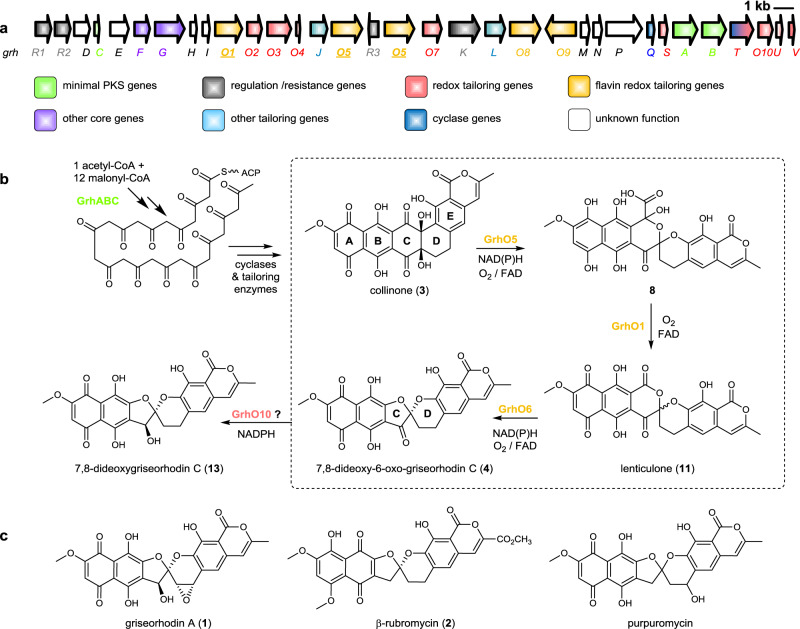
Overview of the proposed biosynthesis of bacterial rubromycin-type polyketides and final pathway products. **a** Griseorhodin A biosynthetic gene cluster encoding, e.g., the minimal type II polyketide synthase (PKS), cyclases, and tailoring enzymes^9^. **b** Initial steps afford a reactive acyl-carrier protein (ACP)-bound poly-β-ketone, which is subsequently cyclized and modified to **3**. Compounds **3** and **11** were previously identified in the course of gene deletion experiments (ΔgrhO5 and ΔgrhO6, respectively, encoding flavin-dependent tailoring enzymes investigated in this work) and assigned as putative advanced intermediates^10^. The conversion of **3** into **4** via **8** and **11** (dashed box) and additional intermediates was elucidated in this work. A ketoreductase (presumably GrhO10) then converts **4** into **13**. **c** Examples of mature rubromycins likely formed from **13**.

The benastatins, pradimicins, fredericamycins, xantholipins (among others), as well as the rubromycin family belong to a growing group of biosynthetically related aromatic type II polyketide natural products with extended “pentangular” architecture that are produced by numerous actinobacterial species^1–8^. The intensely colored rubromycins comprise various griseorhodins (e.g., griseorhodin A (**1**)), hyaluromycin, purpuromycin, heliquinomycin, as well as the eponymous rubromycins (e.g., β-rubromycin (**2**))^2,9–12^. Often, these compounds act as potent enzyme inhibitors and **2** was consequently suggested as a lead structure for drug development^13^. The hallmark structural feature of the rubromycins is a bisbenzannulated [5,6]-spiroketal pharmacophore that disrupts the planarity of the polycyclic, aromatic carbon backbone (Fig. 1)^13^. Even though **2** was first described in 1953^11^ (Fig. 1), it took almost half a century before the total synthesis of a rubromycin polyketide was achieved owing to their intricate structures^12,14^. In fact, synthetic strategies for the griseorhodins have yet to be reported, which are distinguished by even more complex, highly oxygenated pharmacophores such as the epoxyspiroketal of **1**^12^.

Moreover, details on the biosynthesis of the rubromycins and the spiroketal moiety remain scarce. First insights originated from extensive gene-inactivation studies with the griseorhodin (*grh*) A biosynthetic gene cluster of *Streptomyces* sp. JP95 isolated from the marine tunicate *Aplidium lenticulum*, which was expressed in the heterologous producer *S. albus* sp. J1074^9,10^. Initial steps resemble typical type II polyketide pathways involving a minimal polyketide synthase (PKS) that likely utilizes an acetyl-CoA starter unit and 12 malonyl-CoA extender units to generate a highly reactive acyl-carrier protein (ACP)-bound poly-β-ketone chain. Following enzyme-catalyzed regioselective ketoreduction, cyclization, aromatization and ACP elimination, further tailoring reactions modify the polyketide backbone and lead to the advanced and highly oxidized intermediate collinone (**3**) (previously also isolated from a heterologous producer expressing parts of the rubromycin biosynthetic gene cluster^15^), which may serve as a direct precursor for spiroketalization^10^. This would necessitate an extensive oxidative backbone rearrangement as well as the elimination of two C_1_ units, which may be mediated by mechanistically versatile flavin-dependent enzymes^16–22^ that often facilitate redox tailoring reactions in natural product biosynthesis (Fig. 1)^16,19^.

Here, we report the full in vitro reconstitution of enzymatic spiroketal formation in the biosynthesis of rubromycin-type polyketides. We elucidate the conversion of **3** into the [5,6]-spiroketal-containing 7,8-dideoxy-6-oxo-griseorhodin C (**4**) via various reactive intermediates by the concerted action of the flavoprotein monooxygenases GrhO5 and GrhO6, as well as the flavoprotein oxidase GrhO1 that are encoded by the *grh* gene cluster. This process is primarily mediated by the multifunctional monooxygenase GrhO5 that oxidatively rearranges the carbon backbone and ultimately forms a [6,6]-spiroketal and is assisted by GrhO1, before the ring-contracting GrhO6 generates the [5,6]-spiroketal pharmacophore found in mature rubromycin polyketides (Fig. 1).

## Results

### Flavoprotein monooxygenase GrhO5 initiates spiroketal formation by rapid collinone reduction

*S. albus* sp. J1074 KR8 (Δ*grhO5*) was previously reported to produce **3** as major shunt metabolite, which may thus represent the native substrate of GrhO5^10^. To investigate this, **3** was isolated from the *S. albus* mutant, while GrhO5 (fused with an N-terminal maltose binding protein tag) was obtained from the heterologous producer *Escherichia coli* BL21 DE3 (see Online Methods section for details on gene cloning as well as production and purification of enzymes and compounds). GrhO5 is predicted to function as flavoprotein monooxygenase based on the amino acid sequence^10^ and is homologous to the NAD(P)H- and FAD-dependent class A flavoprotein monooxygenases with “glutathione reductase type” Rossmann fold^21^. Typically, these enzymes catalyze aromatic hydroxylation reactions via an electrophilic flavin-C4a-hydroperoxide oxygenating species, while some members instead act as Baeyer–Villiger monooxygenases (BVMOs) that employ a nucleophilic flavin-C4a-peroxide anion^22,23^. The purified enzyme showed an intense yellow coloration indicative of a bound flavin cofactor that was further determined as flavin adenine dinucleotide (FAD; Supplementary Fig. 1). Under optimized assay conditions, GrhO5-dependent consumption of **3** could indeed be observed by UV-Vis spectroscopy in the presence of the electron donor NADPH (≈20% activity with NADH; see Supplementary Fig. 2 for kinetics). To further investigate this and elucidate the reaction course, samples from enzyme reactions were quenched after different incubation times, the compounds extracted and then analyzed by reverse-phase high performance liquid chromatography (RP-HPLC).

**Fig. 2:**
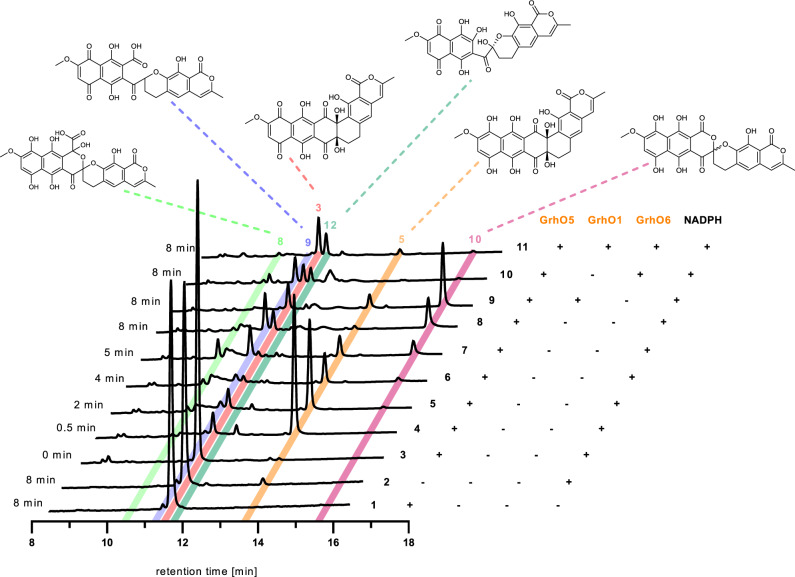
Enzyme assays with substrate **3** in presence of O_2_ (shown are the RP-HPLC chromatograms at *λ* = 254 nm). Incubation times before reaction quenching are indicated to the left. The respective enzyme and cofactor composition is shown to the right. No conversion of **3** was observed in control assays lacking NADPH (trace 1) or GrhO5 (trace 2). Traces 3–8 show time points from the same, discontinuous assay with GrhO5, in which shunt product **9** accumulated aside from intermediate **10**. Addition of GrhO1 boosted **10** formation and counteracted **9** formation (trace 9). Addition of GrhO6 (traces 10 and 11) lead to conversion of **10** into **4** (that rapidly forms ring-opened **12**). Note that **7** is not observed due to poor separation resulting from polymerization and irreversible binding. The structures of the intermediates are shown above. The proposed enzymatic steps are presented in Fig. 3. All assays were at least conducted three times independently and representative examples are shown (for uncropped chromatograms, see Supplementary Fig. 8).

**Fig. 3:**
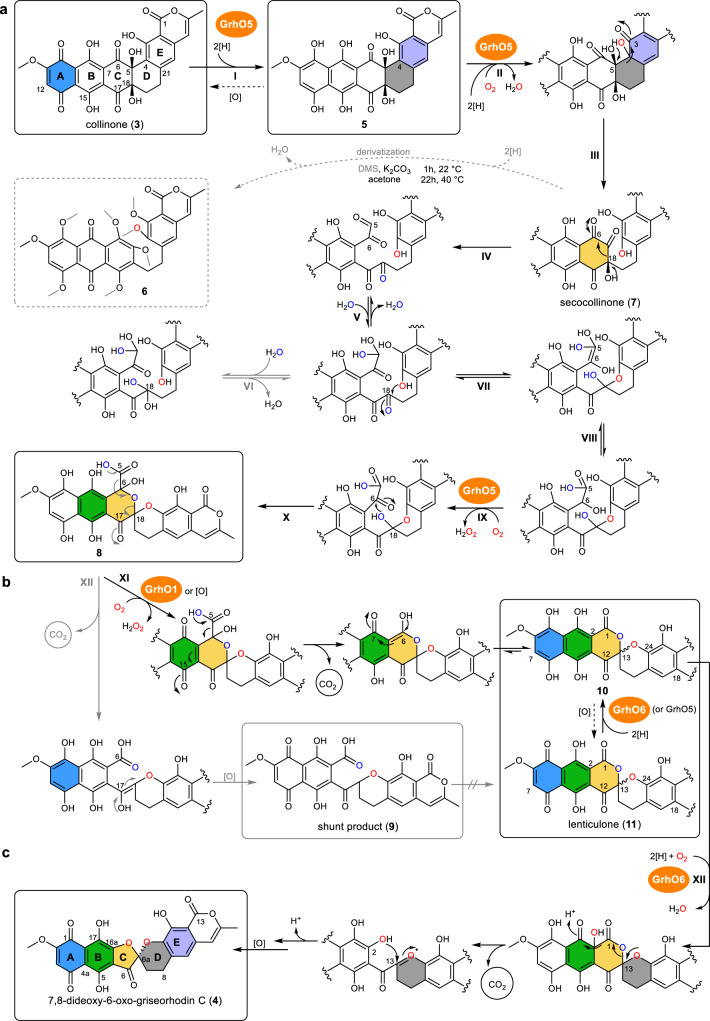
Proposed spiroketal formation in rubromycin polyketide biosynthesis. Redox steps catalyzed by GrhO5, GrhO1, and GrhO6 are highlighted with the respective enzyme symbols. Other steps may be catalyzed or occur spontaneously in the enzymes’ active sites, see text for details. The rings A–E of compounds that undergo modification in the ensuing biosynthetic step are color-coded according to the final product **4** (bottom left); important carbons are numbered for each step. Note that the carbon numbering of intermediates up to **8** is according to compound **3**, whereas different carbon numberings are used for **10**/**11** and the final product **4**. Oxygen atoms derived from O_2_ and H_2_O (based on isotope-labeling experiments) are highlighted in red and blue, respectively. Water-derived ^18^O is most likely also incorporated from spontaneous and reversible keto hydration as shown in step VI (gray arrows). **a** Proposed GrhO5-mediated conversion of **3** into **8**. For secocollinone detection and structural characterization by NMR, derivatization to **6** by dimethyl sulfate (DMS) was required (gray dashed box). **b** Formation of on-pathway intermediate **10** (black arrows) and shunt product **9** (gray arrows) from **8**. GrhO1 boosts **10** formation, while **9** production is minimized (see Fig. 2). **c** Proposed GrhO6-catalyzed formation of **4** from **10**/**11**. Presumably, **3** and **11** are largely skipped as intermediates in the reducing environment of the cell. Dashed arrows indicate autooxidation steps. Electron-dependent steps (indicated by 2[H]) require NADPH as preferred reductant for both GrhO5 and GrhO6, see text. The spiroketal-configuration of **11** (racemic) and **4** (chiral) were analyzed by CD spectroscopy, see text. All intermediates in boxes were characterized by NMR and/or HRMS. Note that the final product **4** spontaneously converts to **12** (see below).

First, GrhO5 catalyzed the rapid conversion of **3** into intermediate **5** (Supplementary Fig. 3). Extracted **5** featured a distinct UV-Vis spectrum and intense yellow color, as compared to the purple-red **3**. Liquid chromatography high-resolution mass spectrometry (LC-HRMS) indicated that **5** represents a reduced form of **3**, which spontaneously reoxidized in the presence of O_2_, as shown by the color change and confirmed by RP-HPLC (Supplementary Fig. 3). This was further supported by the non-enzymatic chemical reduction of **3** (using Ti(III) citrate or DTT), which also afforded **5** (Supplementary Fig. 3a). Notably, compared to the much faster GrhO5-dependent **5** formation, NADPH (±free FAD) only reduced **3** at very low rates (Fig. 2 and Supplementary Fig. 2c). To solve the structure of **5** and of other compounds described below, large scale enzymatic assays were conducted. Anaerobic conditions enabled the complete conversion of **3** into **5**, which was afterwards extracted, purified via RP-HPLC, and lyophilized. NMR spectroscopy (^1^H NMR, ^13^C NMR, HSQC, HMBC, Supplementary Figs. 4–7) in a sealed, anaerobic tube then identified **5** as ring A-reduced dihydrocollinone featuring a naphthohydroquinone moiety (Fig. 3a).

### Dihydrocollinone is oxygenolytically cleaved by GrhO5 and converted to secocollinone

Compound **5** was not further processed by GrhO5 under anaerobic conditions, suggesting a succeeding O_2_-dependent reaction probably in form of an oxygenation. Indeed, in presence of O_2_, **3** was first reduced to **5** and then further converted; however, significantly reduced amounts of putative intermediates/products were subsequently retrieved. We thus suspected that GrhO5 converts **5** into a labile and/or insoluble intermediate. To investigate this, chemical derivatization of the putative intermediate was attempted after quenching of the enzyme assay. Methylation of the reactive phenol groups by dimethyl sulfate (DMS) proved suitable and afforded a completely methylated and reduced derivative (**6**) of an enzymatically hydroxylated intermediate based on LC-HRMS and tandem MS (MS^2^) analysis (Supplementary Fig. 9). Comprehensive NMR spectroscopy (Supplementary Figs. 10–13) of **6** revealed a ruptured D-ring as well as a hydroxylated E-ring. We propose the native intermediate to be secocollinone **7**, which is likely derivatized to **6** by non-enzymatic reduction (via NADPH), keto-enol tautomerization (that triggers water elimination at C18), and complete methylation of the free hydroxyl groups (Fig. 3a).

To confirm that **7** represents an intermediate, the time course for **6** formation was scrutinized in the GrhO5 assay. For that, samples were withdrawn at different time points, quenched, and methylated. HPLC analysis of the derivatized samples then showed the formation of significant amounts of **6** at 1 min, a maximum amount at 3 min, and only residual amounts after 4 min, consistent with the envisaged formation of **7** from **5** (Fig. 3a and Supplementary Fig. 9a). Based on these results, we propose the following reaction sequence. First, ring A of **3** is reduced to **5** (Fig. 3a, step I), before aromatic hydroxylation at C4 of **5** (step II) prompts the first C–C bond cleavage of ring D via retro-aldol condensation to afford **7** (step III). The flavin-mediated hydroxylation most likely proceeds via an electrophilic flavin-C4a-hydroperoxide (Fl_C4aOOH_) oxygen transferring agent, as discussed below.

### Secocollinone is rearranged to the [6,6]-spiroketal-containing dihydrolenticulone

Concurrent with consumption of **7**, compound **8** emerged in the HPLC chromatograms, followed shortly by shunt product **9** and on-pathway intermediate **10** (Figs. 2 and 3ab). Notably, isolated **8** proved highly unstable and LC-HRMS analysis verified the spontaneous conversion into both **9** and **10** (Supplementary Fig. 14). Moreover, compound **10** rapidly autooxidized to compound **11**, analogous to the oxidation of **5** into **3**, as verified by RP-HPLC and LC-HRMS (Supplementary Fig. 15). Compounds **9** and **11** were only moderately stable in aqueous solution, thus explaining why prolonged incubation (>30 min) of **3** with GrhO5 did not yield significant amounts of enzymatic product. Surprisingly, **11** was identified as the previously reported lenticulone featuring a [6,6]-spiroketal^10^ and **10** accordingly as the respective ring A-reduced dihydrolenticulone based on LC-HRMS/MS^2^ analysis, UV-Vis spectra, NMR spectroscopy, and comparison with a standard acquired from *S. albus* sp. J1074 MP66 (Δ*grhO6*)^10^ (Fig. 3b and Supplementary Figs. 16–19). In contrast, comprehensive NMR spectroscopy and MS^2^ fragmentation of shunt product **9** revealed a non-spiro compound with opened C-ring (Fig. 3b, Supplementary Figs. 20–25, and Supplementary Table 1).

To consolidate these results, we propose that **7** first undergoes retro-aldol cleavage of ring C generating an α-keto aldehyde motif at C5–C6 (Fig. 3a, step IV). Then, a series or reversible steps takes place including carbonyl-hydrations, tautomerization, and ring D re-formation via hemiacetal formation (steps V–VII), before isomerization affords a C5–C6 α-hydroxy acid (step VIII). Subsequently, the C6 hydroxyl group is oxidized to the respective ketone, likely mediated by the flavin cofactor of GrhO5 (step IX). Lastly, ring C is reinstalled via attack of the C18 hydroxyl group on the C6-ketone to afford **8** (step X). Although highly labile **8** could not be isolated for structural elucidation by NMR, isotope-labeling studies with **8** corroborate this proposal. Accordingly, up to two ^18^O atoms derived from H_2_^18^O were observed in **8** by LC-HRMS/MS^2^ (Supplementary Fig. 26), consistent with water-dependent α-hydroxy acid formation (part of steps V–VIII) and incorporation of ^18^O via reversible carbonyl hydrate formation (step VI).

Next, we propose that spontaneous oxidation of ring B of **8** allows the third C–C bond cleavage via facile decarboxylation. This is enabled by an appropriately positioned C15-ketone in ring B that acts as electron sink, thereby producing on-pathway intermediate **10** after keto-enol tautomerization, before ring A autooxidation yields **11** (step XI, Fig. 3b). Interestingly, in addition to reduction of **3**, GrhO5 was also capable of reducing **11** into **10** with NAD(P)H (Supplementary Fig. 17). Alternatively, decarboxylation can occur (without prior oxidation) through spiroketal-opening enabled by the C17-ketone of ring C to afford shunt product **9** after tautomerization and autooxidation of ring A (step XII). To further investigate this mechanistic proposal, both **9** and **11** were enzymatically produced in presence of either ^18^O_2_ or H_2_^18^O. As expected, one ^18^O label from O_2_ was incorporated in the eastern molecule halves of **9** and **11** as a result of GrhO5’s oxygenase functionality (Supplementary Figs. 27 and 28). Notably, in the labeling experiments with H_2_^18^O, both **9** and **11** only retained a single ^18^O-label from H_2_^18^O in the western molecule half (Supplementary Figs. 27 and 28 and Supplementary Tables  2–5) in contrast to the doubly labeled **8**, confirming that one water-derived ^18^O was eliminated via decarboxylation (Fig. 3b). The UV-Vis data were furthermore fully consistent with the proposed redox states of these intermediates and clearly indicated a napthohydroquinone moiety with reduced rings A and B for **5**, **8**, and **10** (Supplementary Fig. 29a), while spectral features of **3**, **9**, and **11** suggested oxidized quinonic A rings (Supplementary Fig. 29b).

### The quinone-forming oxidase GrhO1 boosts dihydrolenticulone formation

Although GrhO5 proved sufficient for in vitro formation of **10**, ≈50% of shunt product **9** was generated. This prompted us to consider additional enzymes that could mediate the oxidation of ring B of **8** and thus promote formation of **10**/**11**. Previous studies with deletion mutants suggested that GrhO1 may be involved in spiroketal maturation^10^. GrhO1 also seemed a prime candidate because it is a member of the vanillyl alcohol oxidase/*para*-cresol methylhydroxylase (VAO/PCMH) flavoenzyme family that typically catalyze (non-oxygenative) two-electron oxidation reactions rather than hydroxylations, although some exceptions exist^18,24^. Hence, we supplemented our in vitro GrhO5 assays with heterologously produced polyhistidine-tagged GrhO1 that features a covalently bound flavin (Supplementary Fig. 30). Indeed, the presence of GrhO1 minimized production of **9** and boosted formation of **10** (Fig. 2), whereas purified **9** (and its reduced form) could not be converted by GrhO1 (Supplementary Fig. 31). These results indicate that GrhO1 indeed catalyzes the selective oxidation of ring B of **8** (step XI, Fig. 3b) and assists GrhO5 in [6,6]-spiroketal formation. Next, we analyzed the GrhO5 and GrhO1-produced **11** by circular dichroism (CD) spectroscopy, revealing a racemic mixture. Presumably, enzymatically formed **11** is chiral at first but undergoes spontaneous racemization via rapid ring-opening and recylization. Consistent with that, the racemate could not be separated by chiral HPLC (Supplementary Fig. 32).

### GrhO6 completes formation of the mature [5,6]-spiroketal moiety

GrhO6 is also predicted to be a member of the class A flavoprotein monooxygenases and has 31.4% amino acid (aa) identity (70% sequence coverage) with GrhO5. The Δ*grhO6* mutant strain was previously shown to accumulate **11**, suggesting a possible role for GrhO6 in spiroketal formation^10^. Hence, polyhistidine-tagged GrhO6 was heterologously produced and isolated, which contained a tightly, but non-covalently bound FAD cofactor similar to GrhO5 (Supplementary Fig. 33). Indeed, upon complementing the GrhO5/GrhO1 assay with GrhO6, a stable main product **12** was formed in vitro (Fig. 2). LC-HRMS^2^ and UV-Vis spectra suggested that **12** may represent a derivative of the anticipated final product 7,8-dideoxy-6-oxogriseorhodin C (**4**) with a mass of [**4** + H_2_O] (Supplementary Fig. 34).

**Fig. 4:**
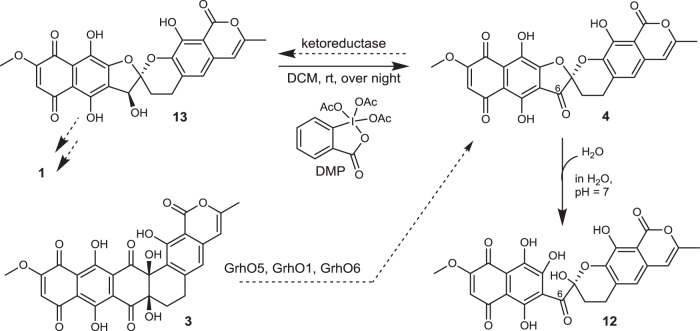
Chemical preparation of **4** from **13** (isolated from *S. albus* KR7^10^). Synthesized **4** converts into **12** in aqueous solution at neutral pH, as also observed in the enzyme assays (Fig. 2). Note that the structure of **12** is supported by NMR and IR data, as well as DFT calculations, but a C6-ketohydrate instead of the shown ring-opened compound **12** cannot be ruled out (see Supplementary Fig. 48). Enzymatic steps are indicated with dashed arrows, non-enzymatic chemical steps with bold arrows. A ketoreductase may deter water addition in vivo en route to **1**. The stereochemical configuration of **13** and **4**/**12** is depicted as previously determined for **1** and verified in this work. DMP: Dess–Martin periodinane, DCM: dichloromethane.

To further investigate this, we cultured *S. albus* sp. J1074 KR7 (Δ*grhO7*) for the production of 7,8-dideoxygriseorhodin C (**13**, Fig. 4)^10^. After confirmation of the authenticity of **13** by HRMS/MS^2^, and NMR (Supplementary Figs. 35–37), we chemically oxidized the secondary alcohol at C6 of **13** using Dess-Martin periodinane to generate **4**. In total, 20 mg of **13** was oxidized to 15 mg of **4**, as verified by HPLC, HRMS/MS^2^, and NMR (Supplementary Figs. 34a, 38a, and 39–42). Indeed, when **4** was dissolved in water and incubated at pH 2 or pH 7 with temperatures ranging from 4 to 50 °C, it was non-enzymatically converted to **12** in aqueous phase at neutral pH (i.e. enzyme assay-like conditions), while remaining largely stable under acidic conditions (Fig. 4 and Supplementary Fig. 43a). When this reaction was carried out in H_2_^18^O, incorporation of ^18^O was observed, suggesting that **12** represents a hydrate of **4** (Supplementary Fig. 43b). Moreover, upon scrutinizing our enzymatic assays, **4** could be detected (Supplementary Fig. 38b). Although NMR analysis of **12** proved inadequate to fully elucidate the structure and chemical derivatization also failed (Supplementary Figs. 44–47), infrared spectroscopy and density functional theory (DFT) computation (Supplementary Fig. 48) suggested that **4** undergoes spiroketal hydrolysis to yield **12**, consistent with NMR, MS, and UV-Vis data (Supplementary Figs. 34, and 44–47). This exergonic reaction (Δ*G*° = −5.1 kJ mol^−1^ according to DFT calculations) is likely driven by the ring strain caused by the sp^2^-hybridized C6-ketone of **4** and proceeds via a transient ring-opened oxocarbenium that is quenched by water to form **12**. Most likely, **4** represents the true GrhO6 product that rapidly converts to **12** when not further processed by downstream pathway enzymes (most likely by a **13**-forming ketoreductase^10^, Figs. 1 and 4).

To confirm that **11** is a substrate for GrhO6, the compound was purified and used for separate in vitro assays with GrhO6. Similar to GrhO5, GrhO6 first reduced **11** into **10** before forming **12**/**4** in the presence of NADPH (≈40% activity with NADH), as quantified by UV-Vis spectroscopy (see Supplementary Fig. 2 for kinetics) and further analyzed by RP-HPLC, whereas **9** was not accepted as substrate (Supplementary Fig. 49a, b). Moreover, the requirement for NAD(P)H suggested a monooxygenase functionality for GrhO6. This was further confirmed by ^18^O_2_-labeling experiments, which revealed the incorporation of ^18^O into the western molecule half (Supplementary Fig. 49c and Supplementary Table 6). GrhO6 likely employs a similar mechanism to GrhO5. Following NAD(P)H-dependent **10** formation, GrhO6 then likely catalyzes an aromatic hydroxylation at C2 of **10** (step XII, Fig. 3c), which dearomatizes ring B and affords a C3-ketone in β-position to the C1-lactone moiety. This enables the subsequent decarboxylation of C1 via formation of a transient oxocarbenium that is quenched through intramolecular nucleophilic attack by the introduced C2-hydroxyl-group and thereby generates the [5,6]-spiroketal of **4** (Fig. 3c). CD spectroscopy verified the chirality of compound **4** that features the same configuration at the C6a as **1**^10,25^, suggesting that [5,6]-spiroketal formation is stereochemically controlled by GrhO6 (Supplementary Fig. 50).

### Metabolomic and genomic analyses support a universal pathway for spiroketal formation in the rubromycin polyketide family

In contrast to **3**, **4**, **11**, the intermediates **5**, **7**, **8**, **9**, **10**, **12** were hitherto uncharacterized. To verify the authenticity of some key pathway intermediates, we extracted liquid cultures at various time points during growth of *S. albus* sp. J1074 (heterologous **1** producer)^10^, *Actinoplanes ianthinogenes* (native producer of purpuromycin^26^), and *S. puniceus* (native producer of **1** and griseorhodin C)^25^. RP-HPLC and LC-HRMS analysis clearly confirmed the presence of **10** (that likewise underwent spontaneous oxidation to **11**) and **12** in various cell free lysates, while **5**, unstable **8**, and shunt product **9** were not observed. Also, **7** could not be directly detected as a result of the poor interaction with chromatographic stationary phases. However, complex compound mixtures including insoluble compounds prone to polymerization were previously reported (but not further characterized) in the Δ*grhO1* mutant strain *S. albus* J1074 KR5^10^. Indeed, upon treatment of the cell free lysate of a culture of this strain with DMS, **6** could be obtained (Supplementary Fig. 9b).

**Fig. 5:**
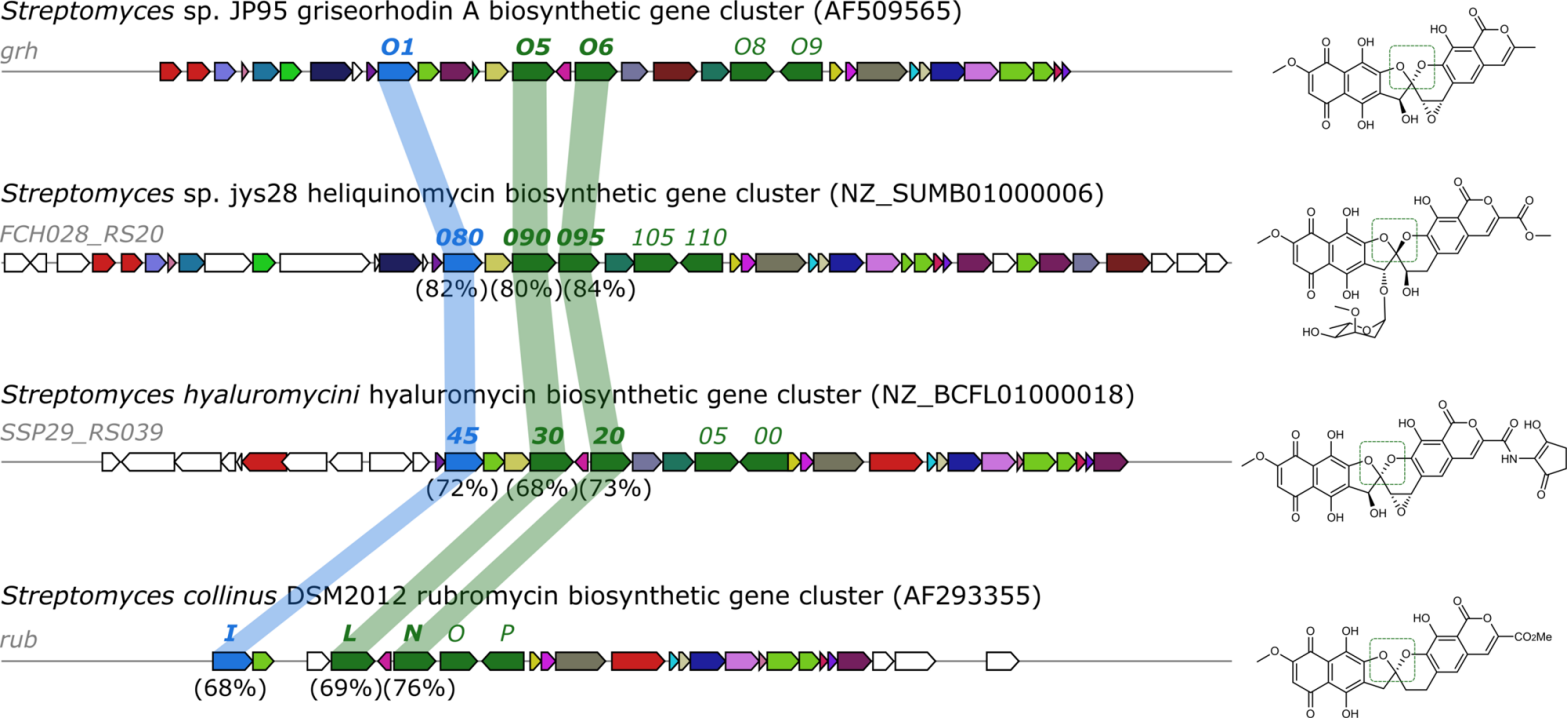
Gene cluster comparison performed with MultiGeneBlast^29^. Same colors indicate high amino acid sequence similarities and predicted similar functions. The biosynthetic gene clusters for production of the spiroketal-containing compounds griseorhodin A (**1**, AF509565), heliquinomycin (NZ_SUMB01000006), hyaluromycin (NZ_BCFL01000018), and rubromycin (**2**, AF293355) are shown. These clusters harbor *grhO5* (green), *grhO1* (blue), and *grhO6* (green) homologs. Corresponding homologs are connected with a shadow and the percentages of the amino acid sequence identities of the encoded proteins are shown. The *grhO8* and *grhO9* genes also show homology to *grhO5* and *grhO6* and encode FAD-dependent monooxygenases involved in early redox tailoring steps. The biosynthetic gene clusters of the non-spiroketal pentangular polyketides lack *grhO1*, *grhO5*, and *grhO6* gene candidates necessary for spiroketal formation (see Supplementary Fig. 51 for full comparison).

Furthermore, genomic data suggest that the biosynthetic gene clusters for production of spiroketal-containing **1** (*S*. sp. JP95), **2** (of *S. collinus*)^15^, hyaluromycin (of *S. hyaluromycini* MB-PO13)^27^, and of heliquinomycin (*S*. *piniterrae*)^28^ harbor *grhO5*, *grhO1*, and *grhO6* homologs (the genome of the bacterial purpuromycin producer is not available yet). Compared to that, respective candidate genes were absent in the biosynthetic gene clusters of the non-spiroketal compounds benastatin A, fredericamycin A (featuring a carbaspirocycle), pradimicin A, lysolipin, and arixanthomycins (Fig. 5 and Supplementary Fig. 51). To further verify a common pathway for spiroketal formation in the rubromycin polyketide family, we acquired heterologously produced polyhistidine-tagged RubL – the predicted functional homolog of GrhO5 encoded by the rubromycin biosynthetic gene cluster – and showed that it catalyzed the virtual identical conversion of **3** into **10/11** (Supplementary Fig. 52).

## Discussion

Ever since their discovery, rubromycin-type polyketides have sparked the interest of synthetic chemists and biochemists because of their complex framework and potent bioactivities. While spiroketal groups are occasionally encountered in natural products, adjacent aromatic rings giving rise to distinct bisbenzannulated spiroketals are exceedingly rare^30^. These spiroketals are remarkably robust owing to the stabilizing effect of the connected aryl functionalities through delocalization of the lone pairs of electrons from the oxygen atoms^30^. In this work, we provide detailed insights into the enzymology and biochemistry underlying the installment of the [5,6]-spiroketal pharmacophore into the backbone of rubromycin natural products. These redox tailoring processes are driven by the multifunctional spiroketalase GrhO5 (assisted by GrhO1) that produces key intermediate **10**, which is subsequently transformed by GrhO6 into **4**. Both GrhO5 and GrhO6 resemble characterized class A flavoprotein monooxygenases, e.g., GrhO5 is related to the aromatic hydroxylase RdmE (40.7% aa identity, 100% coverage) from aklavinone biosynthesis^31^ and to XanO4 (42.3% aa identity, 98% coverage) catalyzing oxygenation and demethoxylation in xanthone biosynthesis^32^. Moreover, GrhO5 resembles the predicted aromatic hydroxylases GrhO8 (45.5% aa identity, 98% coverage) and GrhO9 (44% aa identity, 94% coverage) from **1** biosynthesis that presumably catalyze early tailoring steps en route to **3**^10^. On the other hand, GrhO6 is more closely related to aromatic hydroxylases such as OxyS (43.9% aa identity, 94% coverage) from oxytetracycline biosynthesis^33^ and verified BVMOs, e.g., MtmOIV (45% aa identity, 92% coverage) from mithramycin biosynthesis^34^ (Supplementary Fig. 53).

Our results imply a reaction sequence for GrhO5 that combines versatile redox chemistry (quinone reduction, aromatic hydroxylation, and alcohol oxidation) with various non-redox steps (e.g., retro-aldol ring cleavage and spiroketalization). While flavoenzyme functionalities such as quinone reduction^35^, quinone reduction coupled to aromatic hydroxylation^36^, or alcohol oxidation^37^ have been reported before, a single flavoenzyme that combines these steps and additionally drastically rearranges the carbon backbone is noteworthy. Notably, although initial substrate reduction is not common for flavoprotein monooxygenases^22^, it seems to be a more widely employed strategy in natural product biosynthesis for the activation of quinonic compounds prior to further redox transformations of the resulting hydroquinones or respective diketotautomers, e.g., in aflatoxin or actinorhodin polyketide biosynthesis^36,38–40^. In the case of GrhO5, however, the first reduction of ring A is distant from the actual hydroxylation site and serves a further purpose: it is the requisite for the conversion of **8** into **10**, as a quinonic ring A would deter ring B oxidation and thus lead to accumulation of shunt product **9** (Fig. 3b). The flavoprotein oxidase GrhO1 additionally counteracts **9** generation by catalyzing the selective oxidation of the hydroquinonic ring B to the *p*-quinone and thereby redirecting the metabolic flow to formation of **10** (Fig. 3b). Presumably, oxidized intermediates such as **3** and **10** might be largely skipped in the reductive environment of the cell and the quinone-reducing flavoenzyme functionality of GrhO5 and GrhO6 would thus be primarily required for the salvaging of autooxidation products.

Finally, spiroketal maturation is completed by flavoprotein monooxygenase GrhO6 that converts the [6,6]-spiroketal moiety into the mature [5,6]-spiroketal. Based on the nucleophilicity of C2 of substrate **10**, an aromatic hydroxylation appears likely (an alternative carbonate-producing Baeyer–Villiger oxidation of the C1-lactone^10^ is conceivable, but chemically unfavorable^37^). This hydroxylation then enables the final fourth C–C bond cleavage via decarboxylation, followed by recyclization to the [5,6]-spiroketal of **4** (Fig. 3c). Our combined data suggest a universal flavoenzyme-mediated pathway for spiroketal formation in the rubromycin polyketide family. Presumably, final tailoring steps on the molecular periphery of **4** then expedite the diversification of rubromycin polyketides. Notably, **4** readily formed ring-opened derivative **12** and it remains to be seen if this compound can be further converted or represents a dead-end product. Most likely, keto reduction is required to decrease ring-strain and prevent this non-enzymatic spiroketal-opening in vivo, consistent with the evident lack of mature rubromycins with C6-ketone functionalities.

The complex flavin enzymology underlying the formation of the rubromycin pharmacophore thus provides an astonishing solution for the generation of stable chroman spiroketals in nature. These complex redox transformations of a polycyclic, aromatic backbone starkly contrast with synthetic approaches (that rely on convergent syntheses in which the naphthazarin and isocoumarin moieties are separately generated and combined prior to spiroketal generation^12^) as well as biosynthetic strategies and precursor molecules. Conventional enzyme-dependent routes rely, e.g., on a dihydroxy-ketone motif allowing hemiketal formation and dehydrative cyclization to afford the [6,6]-spiroketal of the pesticide avermectin^41^. Similarly, the proposed cascade-like [5,6]-spiroketal formation in the generation of the polyether antibiotics such as lasalocid A and monensin requires a hydroxy-ketone-epoxide motif and proceeds via initial hemiketal formation followed by epoxide-dependent spiroketalization^42^. Moreover, a radical mechanism has been proposed in griseofulvin biosynthesis^43^. Compared to these straightforward pathways, precursor **3** lacks evident structural requisites and functionalities for spiroketal formation and instead the pentangular precursor undergoes an unanticipated series of oxidative backbone rearrangements. Other noteworthy examples of flavoenzyme-driven oxidative rearrangements in type II polyketide biosynthesis are found, e.g., in chartreusin^44^, xanthone^32^, and rishirilide^45^ biosynthesis. Interestingly, an alternative strategy to disrupt aromatic polyketide planarity is employed in enterocin biosynthesis^18,46,47^, where a single flavoprotein monooxygenase drastically rearranges a linear poly-β-ketone chain^18,24,48,49^ rather than the cyclized backbone. Aside from flavoenzymes, other types of multifunctional monooxygenases (e.g., cytochrome P450s and non-heme iron α-ketoglutarate-dependent enzymes) have also been reported in natural product biosynthesis^50^.

Taken together, this study contributes to our understanding of the catalytic versatility of flavin-dependent enzymes in natural product biosynthesis and lays the foundations for future (chemo)enzymatic formation of novel rubromycin analogs and hybrid compounds. Our work illustrates a counterintuitive principle for the generation of structurally complex polyketide scaffolds and spiroketal pharmacophores.

## Methods

### Gene cloning, heterologous production, and protein purification

#### Gene cloning

The *grhO5*, *grhO1*, and *grhO6* genes were amplified from the cosmid pMP31^9^ containing the *grh* gene cluster (all oligonucleotide primers from Sigma-Aldrich are shown in Supplementary Table 7). The *grhO5* gene was inserted between the NdeI and PstI sites of the expression vector pMAL-c5x-His (New England Biolabs), the *grhO1* gene between the NcoI and EcoRI sites of the expression vector pHis8^51^ and the *grhO6* gene between the EcoRI and PstI sites of the expression vector pETDuet-1 (Novagen, Merck KGaA). The *rubL* gene was synthesized (codon optimized) and purchased already cloned between the NdeI and XhoI sites of the expression vector pET-16b (BioCat).

#### Heterologous production and purification of GrhO5

The recombinant pMal-c5x-His-*grhO5* plasmid was transformed into *E. coli* BL21 (DE3) (Thermo Fisher Scientific). LB-medium was inoculated with the transformed strain and supplemented with 100 µg/mL ampicillin at 37 °C overnight under shaking at 180 rpm. Then, 1 L of TB-medium with 100 µg/mL ampicillin was inoculated with 10 mL of the starter culture and grown to an OD_600_ of 0.5–0.6. The culture was induced with a final concentration of 250 µM IPTG and the cells were grown overnight at 18 °C under shaking (130 rpm). The cells were retrieved by centrifugation at 4000 × *g* for 20 min at 4 °C. Cell pellets were suspended in buffer A (20 mM Tris pH 7.4, 200 mM sodium chloride) with additional 1 mg/mL lysozyme and 1 mM phenylmethylsulfonyl fluoride and then stirred for 30 min at 4 °C. Then, the cells were lysed by sonication and the cell fragments removed by centrifugation at 18,000 × *g* for 30 min at 4 °C. The supernatant was filtered through a 0.45-µm syringe filter and then loaded onto a 5-mL MBPTrap HP Dextrin Sepharose^TM^ column (GE Healthcare) connected to an FPLC system. Following a washing step with buffer A, bound mbp-tagged GrhO5 was eluted by a gradient ranging from 0% to 100% buffer B (20 mM Tris pH 7.4, 200 mM sodium chloride, 10 mM maltose) over 5 column volumes. GrhO5 was concentrated using a 30-kDa MW cut-off MACROSEP spin column (Pall Cooperation) and subsequently desalted with a 5-mL HiTrap desalting column (GE Healthcare). The identity and purity of recombinant GrhO5 was analyzed by SDS-PAGE.

#### Heterologous production and purification of GrhO1

The recombinant pHis8-*grhO1* plasmid was transformed into *E. coli* Lemo21 (DE3). The production and purification was performed according to the procedure described above using kanamycin (50 µg/mL) and chloramphenicol (30 µg/mL) as antibiotics and 750 µM L-rhamnose for optimized expression levels. A 5-mL HisTrap^TM^ FF crude column (GE Healthcare) was used for purification. Unbound protein was removed by washing with buffer A (50 mM Tris pH 7.1, 300 mM sodium chloride, 20 mM imidazole, 10% glycerol) and 0–6% buffer B (50 mM Tris pH 7.1, 300 mM sodium chloride, 500 mM imidazole, 10% glycerol) over five column volumes and bound his-tagged protein was then eluted with 100% buffer B. The protein was concentrated using a 10-kDa MW cut-off MACROSEP spin column (Pall Cooperation) and then further purified by size exclusion chromatography with a HiLoad 16/600 Superdex 200 pg column (GE Healthcare) equilibrated with buffer A (50 mM Tris, pH 7.4, 300 mM sodium chloride) and connected to a FPLC system. Finally, 10% (*v*/*v*) glycerol was added to the fractions containing GrhO1. The identity and purity of the eluted protein was confirmed by SDS-PAGE.

#### Heterologous production and purification of GrhO6

The recombinant pETDuet-1-*grhO6* plasmid was transformed into *E. coli* BL21 (DE3) (Thermo Fisher Scientific). The production and purification was performed according to the procedure described above with ampicillin (100 µg/mL) and the respective buffers (buffer A: 50 mM Tris pH 7.1, 300 mM sodium chloride, 20 mM imidazole; buffer B: 50 mM Tris pH 7.1, 300 mM sodium chloride, 500 mM imidazole).

#### Heterologous production and purification of RubL

The recombinant pET-16b-*rubL* (BioCat) was transformed into *E. coli* BL21 (DE3). The production and purification was performed according to the procedure described above with ampicillin (100 µg/mL) and the respective buffers (buffer A: 50 mM Tris pH 7.4, 300 mM sodium chloride, 10% glycerol; buffer B: 50 mM Tris pH 7.4, 300 mM sodium chloride, 500 mM imidazole, 10% glycerol).

#### Collinone (**3**) production and purification

Spores of the *S. albus grhO5* knock-out strain were inoculated into 50 mL of TSB medium supplemented with apramycin (50 µg/mL) and grown at 30 °C, 200 rpm for 24 h. The resulting culture was used to inoculate 3 L of TSB medium distributed to 1 L flasks with stainless-steel springs. Each flask contained 300 mL of culture supplemented with apramycin (50 µg/mL) and was incubated at 30 °C, 200 rpm for 4 days. The cells were harvested by centrifugation and the pellet was suspended in dH_2_O (300 mL). The pellet/water solution was acidified to pH 1 with 2 N HCl, EtOAc (500 mL) was added and the mixture then stirred overnight. The supernatant was also acidified to pH 1 with 2 N HCl and extracted with EtOAc (3 × 500 mL for 1 L culture). The combined extracts of the supernatant were purified by column chromatography [SiO_2_, CH_2_Cl_2_/MeOH = 9:1 (*ν*/*ν*)]. Fractions containing **3** (detected via thin layer chromatography (TLC)) were combined, dried, and further purified by semi-preparative HPLC. The extract of the pellet fraction was directly purified by semi-preparative HPLC. An average of 10 mg/L cell culture of **3** was obtained. The purity of the red solid **3** was confirmed by high-resolution liquid chromatography mass spectrometry (LC-HRMS) and 1D NMR analysis. The acquired NMR data are in accordance with previously published data^2,15^. TLC (CH_2_Cl_2_:MeOH, 9:1 *v*/*v*): *R*_f_ = 0.59; UV/Vis (CH_3_CN/H_2_O): *λ* 244, 340, 518 nm; HRMS (*m*/*z*): [M + H]^+^ calcd. for C_27_H_18_O_12_, 535.087; found, 535.088.

#### Lenticulone (**11**) production in *S. albus* KR42

Spores of the *S. albus* KR42 strain were inoculated into 50 mL of LB medium supplemented with apramycin (50 µg/mL) and grown at 30 °C, 200 rpm for 4 days. The cells were harvested by centrifugation and the supernatant was acidified to pH 3 with 2 N HCl and extracted with EtOAc (2 × 100 mL). The solvent of the combined extracts was removed under reduced pressure and the residue dissolved in CH_3_CN and EtOAc. After centrifugation for 10 min at 18,000 × *g*, samples were analyzed by HPLC using a NUCLEODUR 100-5 C18ec column (varioprep, 250 mm × 10 mm ID, 5 µM, Macherey-Nagel) coupled with a UNIVERSAL RP guard column (4 mm × 3 mm ID, Macherey-Nagel).

#### Photometric assays for kinetic parameters of GrhO5 and GrhO6

The kinetic parameters for GrhO5 and GrhO6 were determined photometrically at wavelengths of 556 for collinone (**3**) and and 536 nm for lenticulone (**11**) using a Shimadzu Photometer (UV-1650PC, Shimadzu, Duisburg, Germany). A mixture of 50 mM Tris pH 7.4, 1.5 mM NADPH or NADH and substrate (16, 39, 79, 392 µM collinone (**3**) or 20, 50, 100 µM lenticulone (**11**), both dissolved in CH_3_CN) was prepared. The reaction was started by addition of 1 µM GrhO5 or GrhO6 (or free FAD as control) that allowed the measurement of the linear decrease in absorption. The turnover rates were calculated based on extinction coefficients of 4850 L × mol^−1^ × cm^−1^ at 556 nm (**3**) and 1700 L × mol^−1^ × cm^−1^ at 536 nm (**11**) according to Beer-Lambert law. The slope of the absorption was read out by the software (UV Probe 2.43, Shimadzu).

#### Standard assay with collinone (**3**) and GrhO5 (or RubL)

A mixture of 50 mM Tris pH 7.4, 1.5 mM NADH and NADPH, 10 µM GrhO5, and 1% (*ν*/*ν*) hydroxypropyl-beta-cyclodextrin (HPCD) was prepared. The reaction was started by addition of collinone (0.25 mM, dissolved in CH_3_CN, final conc. of 2.5% (*ν*/*ν*)) before incubation for 0.5–8 min at 30 °C while shaking at 750 rpm. The reaction was terminated by addition of EtOAc/formic acid [9:1 (*ν*/*ν*)] and extracted by vigorous shaking. The mixture was centrifuged for 30 s and the layers then separated. The solvent was removed under reduced pressure and the compounds dissolved in CH_3_CN (20 µL) and EtOAc (20 µl). After centrifugation for 10 min at 18,000 × *g*, samples were analyzed by HPLC using a NUCLEODUR 100-5 C18ec column (varioprep, 250 mm × 10 mm ID, 5 µM, Macherey-Nagel) coupled with a UNIVERSAL RP guard column (4 mm × 3 mm ID, Macherey-Nagel). The assay was also performed using identical conditions with RubL instead of GrhO5.

#### Enzymatic assay with GrhO5, GrhO1, and GrhO6

The enzyme assay was performed identical to the standard GrhO5 assay with additional 10 µM of GrhO6 and/or 5 µM of GrhO1. Reactions were quenched after 8 min and analyzed as described above.

#### Enzymatic assay to isolate dihydrocollinone (**5**)

The enzymatic assay was performed under anaerobic conditions using the above described assay composition. The reaction was started in an anaerobic chamber by adding solution A (50 mM Tris, 1 µM GrhO5, 1% (*v*/*v*) HPCD and collinone (0.25 mM, dissolved in CH_3_CN, final conc. of 2.5% (*v*/*v*)) to solution B (10% glycerol, 1.5 mM NADH and NADPH). After 1 min the reaction mixture was extracted [EtOAc/formic acid = 9:1 (*ν*/*ν*)] inside the anaerobic chamber. The mixture was centrifuged for 30 s and the layers then separated inside the anaerobic chamber. The next steps were performed under minimal exposure to O_2_. The solvent was removed under reduced pressure and the residue dissolved in CH_3_CN and EtOAc. After centrifugation for 10 min at 18,000 × *g*, compound **5** was purified by HPLC using a NUCLEODUR 100-5 C18ec column (varioprep, 250 mm × 10 mm ID, 5 µM, Macherey-Nagel) coupled with a UNIVERSAL RP guard column (4 mm × 3 mm ID, Macherey-Nagel). The yield of **5** was ~12 mg of orange solid. The product was analysed by ^1^H, ^13^C, 2D NMR, UV/Vis and ESI-HRMS. ^1^H NMR (600 MHz, CDCl_3_): *δ* 2.07–2.11 (m, 1H), 2.18–2.21 (m, 1H), 2.30 (s, 3H), 2.92–2.96 (m, 1H), 3.33–3.39 (m, 1H), 3.61 (brs, 1H), 4.00 (s, 3H), 6.24 (s, 1H), 6.35 (brs, 1H), 6.73 (s, 1H), 6.88 (s, 1H), 9.55 (s, 1H), 9.89 (s, 1H), 12.29 (s, 1H), 14.68 (brs, 1H), 15.48 (s, 1H).; ^13^C NMR (150 MHz, CDCl_3_): *δ* 19.4, 26.1, 29.1, 56.5, 76.6, 80.1, 100.6, 102.8, 103.8, 104.2, 104.6, 107.5, 114.9, 115.7, 117.7, 137.3, 141.0, 147.4, 152.2, 154.0, 154.9, 160.4, 161.4, 162.7, 167.0, 196.0, 197.1; UV/Vis (CH_3_CN/H_2_O): *λ* 259, 338, 455, 481 nm; and HRMS (m/z): [M-H]^−^ calcd. for C_27_H_20_O_12_, 535.088; found, 535.088.

#### Enzymatic assays with compound **9**

A solution of 50 mM Tris pH 7.4, 1.5 mM NADH/NADPH, 10 µM of either GrhO5, GrhO1 or GrhO6, and 1% (*ν*/*ν*) HPCD) was prepared. The reaction was started by addition of compound **9** (dissolved in CH_3_CN, final conc. of 2.5% (*ν*/*ν*) CH_3_CN in assay) before incubation for 5 min at 30 °C while shaking at 750 rpm. The reaction was quenched by addition of EtOAc/formic acid [9:1 (*ν*/*ν*)] and extracted by vigorous shaking. The mixture was centrifuged for 30 s and the layers then separated. The solvents were removed under reduced pressure and the compounds dissolved in CH_3_CN (20 µl) and EtOAc (20 µl). After centrifugation for 10 min at 18,000×*g*, samples were analyzed by HPLC.

#### Enzymatic assay to isolate compound **9** and compound characterization

A large scale enzyme assay (200 mL distributed to Eppendorf tubes each containing 500 µL) was performed using the same composition of constituents as described for the standard GrhO5 assay. The reaction was quenched after 15 min and compound **9** purified as described above for **5** but under aerobic conditions. The yield of **9** was ~2 mg of red solid. The product was analysed by ^1^H, ^13^C, 2D NMR, UV/Vis, and ESI-HRMS. ^1^H NMR (400 MHz, DMSO-d_6_, 1% TFA-d): *δ*2.04–2.08 (m, 2H), 2.15 (s, 3H), 2.61–2.65 (m, 2H), 3.96 (s, 3H), 6.33 (s, 1H), 6.55 (s, 1H), 6.57 (s, 1H), 10.71 (brs, 1H), 12.13 (brs, 1H), 13.01 (s, 1H); ^1^H NMR (400 MHz, 1,4-dioxane-d_8_): *δ* 2.10–2.16 (m, 5H), 2.73–2.78 (m, 2H), 3.94 (s, 3H), 5.24 (brs, 1H), 6.13 (s, 1H), 6.39 (s, 1H), 6.55 (s, 1H), 8.10 (brs, 1H), 10.82 (brs, 1H), 12.92 (brs, 1H); ^13^C NMR (100 MHz, DMSO-d_6_, 1% TFA-d) *δ* 18.6, 24.3, 33.7, 57.6, 76.9, 103.9, *103.9*, 104.2, 110.7, 116.1, 118.4, *128.3*, 128.3, 137.4, *141.7*, 141.7, *147.3*, 147.3, *151.2*, 151.2, 161.5, *166.1*, 166.1, 183.1, 190.0, 197.9; Most signals are unambiguously assigned, also due to HSQC and HMBC signals. Some signals of quaternary carbon atoms are extraordinarily strong because of the complete overlap of two carbons. The signals in italics show the carbon atoms with lacking HSQC and HMBC signals that are assigned due to chemical shifts observed for biosynthetically related molecules (see Supplementary Figs. 20–24); Notably, the proton attached to C13 could not be detected in DMSO-d_6_, possibly because of a proton exchange with the adjacent carboxyl group or interaction with metals that is often observed for such compounds. However, the measurement in 1,4-dioxane-d_8_ revealed the proton attached to C13 and HRMS as well as extensive MS/MS fragmentation including ^18^O labeled fragments (in particular the diagnostic unlabeled 319 and labeled 321 fragments) strongly support the proposed structure (see Supplementary Figs. 25–27 and Supplementary Table 1–3 for fragmentation). UV/Vis (CH_3_CN/H_2_O): *λ* 232, 364, 503 nm; HRMS (*m*/*z*): [M + H]^+^ calcd. for C_26_H_18_O_12_, 523.087; found, 523.089.

#### Standard assay with lenticulone (**11**) and GrhO6

A solution of 50 mM Tris pH 7.4, 1.5 mM NADH and/or NADPH, 10 µM GrhO6, and 1% (*ν*/*ν*) HPCD was prepared. The reaction was started by addition of lenticulone (dissolved in CH_3_CN, final conc. of 2.5% (*ν*/*ν*) CH_3_CN in assay) before incubation for 30 min at 30 °C while shaking at 750 rpm. The reaction was quenched by addition of EtOAc/formic acid [9:1 (*ν*/*ν*)] and extracted by vigorous shaking. The mixture was centrifuged for 30 s and the layers then separated. The solvents were removed under reduced pressure and the compounds dissolved in CH_3_CN (20 µl) and EtOAc (20 µl). After centrifugation for 10 min at 18,000 × *g*, samples were analyzed by HPLC.

#### Enzymatic assay to isolate lenticulone (**11**) and compound characterization

The large scale enzyme assay (75 mL distributed to Eppendorf tubes each containing 1 mL) was performed using the same composition of constituents as described for the standard GrhO5 assay with additional 5 µM of GrhO1. The reaction was quenched after 10 min and **10**/**11** were isolated as described above for **5** but under aerobic conditions (collected compound **10** subsequently autooxidized to **11**). The yield of **11** was ~1.1 mg of red solid. The product was analysed by ^1^H, ^13^C NMR, UV/Vis, and ESI-HRMS. The acquired NMR data are in accordance with previously published data^10^. UV/Vis (CH_3_CN/H_2_O): *λ* 233, 354, 496 nm; HRMS (*m*/*z*): [M + H]^+^ calcd. for C_26_H_16_O_12_, 521.072; found, 521.070.

#### Reduction of lenticulone (**11**)

The enzymatic assay was performed under anaerobic conditions. The reaction was started in an anaerobic chamber by adding solution A (50 mM Tris pH 7.4, 1.5 mM NADH and/or NADPH, 5 µM GrhO5 or GrhO6 or 1 mM Ti(III)citrate and 1% (*ν*/*ν*) HPCD) to solution B (10% glycerol, NADH and NADPH). After 1 min the reaction mixture was extracted [EtOAc/formic acid = 9:1 (*ν*/*ν*)], the layers separated and the mixture analyzed on a photometer inside the anaerobic chamber.

#### ^18^O-labeling assays with GrhO5

The dioxygen in solution A (Tris pH 7.4, 50 mM, 10 µM GrhO5, and 1.5 mM NADH and NADPH) and solution B (0.5 mM compound **3**, 1% (*v*/*v*) HPCD and 10% glycerol) was thoroughly removed by 10 cycles of treatment with vacuum and nitrogen gas. Solution B was added to solution A in an anaerobic chamber and the reaction was started by injection of about 97% ^18^O_2_ gas (Campro Scientific). The reaction was stirred at room temperature for 1 and 5 min and then extracted [EtOAc/formic acid = 9:1 (*ν*/*ν*)]. The solvent was removed under reduced pressure and the residue dissolved in CH_3_CN (20 µl) and EtOAc (20 µl) before LC-HRMS analysis. The assays with H_2_^18^O were performed corresponding to typical GrhO5 assays with at least 55% of labeled water in the reaction mixture.

#### Secocollinone (**7**) derivatization via methylation and compound characterization

*S. albus* KR5 spores were inoculated into 100 mL of TSB medium and grown overnight. The resulting seed culture was used to inoculate 9 L of TSB medium, which was distributed to thirty 1 L baffled flasks with stainless-steel springs. Flasks were incubated at 200 rpm for 4 days at 30 °C. The cells were harvested by centrifugation and resuspended in H_2_O (500 mL) with trifluoroacetic acid (0.1% TFA; at pH 3). EtOAc (1 L) was added and the solution was stirred overnight at room temperature. After centrifugation, the layers were separated and the solvent of the organic layer was removed under reduced pressure. In total, 1.4 g crude extract was obtained from 9 L of culture. To a suspension of crude extract (1.4 g) of *S. albus* KR5 and anhydrous K_2_CO_3_ (15 g, 108.5 mmol) in dry acetone (150 mL), dimethyl sulfate (DMS) (17 mL, 22.6 g, 179.3 mmol) was added dropwise. The mixture was stirred at room temperature for 1 h and then heated under reflux for 20 h at 40 °C. Afterwards, the mixture was cooled to room temperature and diluted with H_2_O (300 mL). After stirring for additional 30 min, the mixture was extracted with EtOAc (500 mL). The layers were separated and the organic layer was washed with water. The solvent was removed under reduced pressure. The remaining extract was purified by column chromatography [SiO_2_, CH_2_Cl_2_/EtOAc = 1:1 (*ν*/*ν*)]. Fractions containing **6** (detected via TLC) were combined, dried, and further purified by semi-preparative HPLC. After a second purification step, the isolated yield was 4 mg of a yellow solid. The purity of **6** was confirmed by LC-HRMS and NMR analysis. TLC (CH_2_Cl_2_/EtOAc, 1:1 *v*/*v*): *R*_f_ = 0.42; ^1^H NMR (600 MHz, DMSO-d_6_): *δ* 2.16 (s, 3H), 2.83–2.85 (m, 2H), 2.90–2.91 (m, 2H), 3.79 (s, 3H), 3.79 (s, 3H), 3.81 (s, 6H), 3.87 (s, 3H), 3.88 (s, 3H), 3.91 (s, 3H), 3.97 (s, 3H), 6.40 (s, 1H), 7.01 (s, 1H), 7.06 (s, 1H); ^13^C NMR (150 MHz, DMSO-d_6_): *δ* 19.3, 25.6, 31.1, 56.9, 57.1, 61.5, 61.5, 61.6, 62.0, 62.0, 63.1, 102.7, 103.2, 112.5, 115.6, 121.9, 124.8, 128.8, 130.4, 135.4, 135.8, 140.4, 144.4, 147.4, 151.0, 153.9, 153.9, 154.4, 156.2, 156.4, 158.5, 158.6, 180.7, 183.6; UV/Vis (CH_3_CN/H_2_O): *λ* 231, 265, 350, 396 nm; HRMS (*m*/*z*): [M + H]^+^ calcd. for C_34_H_34_O_12_, 635.212; found, 635.214. The methylation of **7** in the course of enzymatic assays with GrhO5 was performed analogously in smaller scale and analyzed by HPLC as described for the standard assays with GrhO5.

#### 7,8-dideoxygriseorhodin C (**13**) and compound characterization

*S. albus* KR7 spores (50 µL) were inoculated into 50 mL LB medium and grown at 30 °C, 200 rpm for 24 h. 5 mL of the starter culture was transferred into 10 flasks containing 300 mL LB medium and cultivation was continued at 30 °C, 200 rpm for 96 h. This procedure was repeated four times resulting in a total culture volume of 12 L. After cell harvesting by centrifugation (60 min, 4000 × *g*, 4 °C) the supernatant was separated from the pellet and the pellet was resupended in H_2_O (4 × 500 mL). Pellet suspensions were acidified with HCl (6 M, 20 mL) in 500 mL portions. EtOAc (2 × 500 mL) was added and the mixture was stirred overnight. The combined organic layers were concentrated under reduced pressure and the residue subjected to a short silica column [SiO2, CH2Cl2/MeOH/AcOH = 95:5:0.1]. Fractions containing compound **13** were combined. Solvents were removed under reduced pressure and further purified via semi-preparative HPLC, which yielded 120 mg of red solid from the pellet. The supernatant was acidified with conc. HCl (10 mL/L) and extracted with EtOAc (1 L culture with 2 × 500 mL). The combined organic layers were filtered and evaporated under reduced pressure yielding 2.60 g of crude extract from the supernatant. Further purification was performed by column chromatography [SiO2, CH_2_Cl_2_/MeOH/AcOH = 98:2:0.1 (*ν*/*ν*) (2 L), then CH_2_Cl_2_/MeOH/AcOH = 95:5:0.1 (*ν*/*ν*)] and provided 750 mg of crude product. The crude product was further purified via semi-preparative HPLC. Solvents were removed under reduced pressure yielding again 11.6 mg of red solid isolated from the supernatant. The overall yield was 131.6 mg of red solid compound **13**. The product was analysed by ^1^H, ^13^C NMR, UV/Vis and ESI-HRMS. The acquired NMR data are in accordance with previously published data^52^. TLC (CH_2_Cl_2_/MeOH/AcOH, 98:2:0.1 *v*/*v*): *R*_f_ = 0.26; UV/Vis (CH_3_CN/H_2_O): *λ* 233, 316, 354, 505 nm; HRMS (m/z): [M + H]^+^ calcd. for C_25_H_18_O_11_, 495.092; found, 495.092.

#### 7,8-dideoxy-6-oxo-griseorhodin C (**4**) and compound characterization

Dideoxygriseorhodin C (**13**) (20.0 mg, 40.5 µmol) was dissolved in dichloromethane (100 mL) by vigorously stirring for 1 h. DMP solution (0.3 M, DCM) (660 µL, 198 µmol, 4.89 eq) was added and the reaction mixture was stirred at room temperature for 24 h. The solvent was removed under reduced pressure at room temperature and the residue dissolved in CH_3_CN (100 µL). After centrifugation (10 min, 18,000 × *g*, 4 °C), **4** was isolated by semi-preparative HPLC. The collected compound **4** was directly frozen in liquid nitrogen and solvents were removed by subsequent lyophilization for at least 24 h yielding 15 mg of **4** (30.5 µmol, 75%) as dark purple solid. The product was analysed by NMR, UV/Vis and ESI-HRMS. The acquired NMR data are in accordance with previously published data^53^. TLC (CHCl_3_/MeOH/AcOH, 95:5:0.1): *R*_f_ = 0.40; UV/Vis (CH_3_CN/H_2_O) *λ* 232, 255, 317, 352, 508 nm; HRMS (m/z): [M + H]^+^ calcd. for C_25_H_16_O_11_ 493.077, found 493.077.

#### Conversion of compound **4** into **12**

A solution of 50 mM Tris pH 7.4, 7,8-dideoxy-6-oxo-griseorhodin C (**4**) (dissolved in CH_3_CN, final conc. of 2.5% (*ν*/*ν*) CH_3_CN in assay) and 1% (*ν*/*ν*) HPCD in aqueous solution at different pH values (pH = 2, 0.01 M HCl and pH = 7.4) was prepared. The mixtures were incubated at different temperatures (4 °C, 30 °C, 50  °C) for 1 h (750 rpm). The reactions were quenched by addition of EtOAc/formic acid [9:1 (*ν*/*ν*)] and extracted by vigorous shaking. The mixture was centrifuged for 30 s and the layers then separated. The solvents were removed under reduced pressure and the residues dissolved in CH_3_CN (40 µl). After centrifugation for 10 min at 18,000 × *g*, samples were analyzed by HPLC.

#### Isolation of compound **12** and compound characterization

*A. ianthinogenes* spores (60 µL) were inoculated into 60 mL GYM medium and grown at 30 °C and 200 rpm for 48 h. 5 mL each of the preculture were then transferred into 10 flasks equipped with a stainless-steel spring containing 200 mL GYM medium and culture was continued at 30 °C and 200 rpm for ~96 h. The metabolite production was monitored by HPLC. If sufficient amounts of compound **12** were detected, the culture was harvested by centrifugation (30 min, 4000 × *g*, 4 °C). The separated supernatant was acidified with HCl (6 M to pH = 1) before dichloromethane (700 mL) was added. The mixture was stirred vigorously for 1 h followed by centrifugation (10 min, 2000 × *g*, 4 °C). The solvent of the combined extracts was removed under reduced pressure and the residue dissolved in CH_3_CN. After centrifugation for 10 min at 18,000 × *g*, samples were analyzed and compound **12** isolated by HPLC using a NUCLEODUR 100-5 C18ec column (varioprep, 250 mm × 10 mm ID, 5 µM, Macherey-Nagel) coupled with a UNIVERSAL RP guard column (4 mm × 3 mm ID, Macherey-Nagel). The purification via semi-preparative HPLC yielded 31 mg of dark purple solid. The product was analysed by ^1^H, ^13^C and 2D NMR, UV/Vis and ESI-HRMS. ^1^H NMR (600 MHz, DMSO-d_6_, 1% TFA-d): *δ* 2.15 (s, 3H), 2.34 (m, 2H), 2.73–2.75 (m, 2H), 3.91 (s, 3H), 6.30 (s, 1H), 6.62 (brs, 1H), 6.64 (s, 1H), 9.30 (brs, 1H), 10.75 (s, 1H), 12.22 (s, 1H); ^13^C NMR (150 MHz, DMSO-d_6_, 1% TFA-d): *δ* 18.6, 24.4, 34.1, 56.9, 103.9, 104.1, 109.8, 116.2, 128.2, 137.4, 141.7, 147.3, 151.0, 158.2, 166.2, 194.2; The signals of 16 carbon atoms are unambiguously assigned, also due to HSQC and HMBC signals. The remaining nine carbon atoms are not detectable possibly due to interaction with metals that is often observed for such compounds. However, IR spectroscopy and DFT calculations (Supplementary Fig. 48) as well as high-resolution MS/MS spectrometry and UV-Vis data (Supplementary Fig. 34: UV-Vis spectrum is indicative of intact western napthoquinone moiety of which some NMR signals are missing; the MS/MS fragmentation pattern resembles compound **4**) strongly support this structure; UV/Vis (CH_3_CN/H_2_O): *λ* 235, 321, 374, 508 nm; HRMS (m/z): [M + H]^+^ calcd. for C_25_H_18_O_12_, 511.087; found, 511.087.

#### High Performance Liquid Chromatography

Compounds from enzymatic assays were analyzed or prepared by RP-HPLC on a 1100 series chromatographic system (Agilent Technologies) with a VP NUCLEODUR 100-5 C18ec column (250 × 10 mm ID, 5 µM, Macherey-Nagel) and a UNIVERSAL RP guard column (4 × 3 mm ID, Macherey-Nagel). The mobile phases were water with 0.1% trifluoroacetic acid (TFA; solution A) and acetonitrile with 0.1% TFA (solution B). A gradient was run with 2% B from 0 to 1 min, 2–100% B from 1 to 15 min, 100–2% B from 20 to 21 min and 2% B from 21 to 24 min. As second purification step for NMR samples, a Kinetex® Biphenyl 100 Å column (250 × 10 mm ID, 5 µM, Phenomenex) was used with a UNIVERSAL RP guard column (4 × 3 mm ID, Macherey-Nagel). The mobile phases were water with 0.1% TFA or 0.1% formic acid (FA; solution A) and acetonitrile with 0.1% TFA or 0.1% FA (solution B). Isocratic methods were used for compound **6** (90% B) and compound **9** (60% B) at a flow rate of 3–5 mL/min at 30 °C and absorbance was measured at 254 nm as well as 350 nm, 488 nm, and 500 nm. For analysis of the flavin cofactors, a NUCLEODUR Sphinx RP (150 × 3 mm ID, 5 µM, Macherey-Nagel) with a UNIVERSAL RP guard column (4 × 3 mm ID, Macherey-Nagel) was used. The mobile phases were water with 0.1% TFA (solution A) and acetonitrile with 0.1% TFA (solution B). The following gradient was used: 2% B for 0–1 min, 2–100% B from 1 to 15 min, 100% B from 15 to 20 min and 2% B from 20 to 21 min with a flow rate of 1 mL/min at 30 °C. Absorbance was measured at wavelengths of 210 nm, 254 nm, 260 nm, and 280 nm.

#### Chiral HPLC analysis

Dissolved and filtered samples of lenticulone (**11**) were analyzed by chiral HPLC on a 1100 series chromatographic system (Agilent Technologies) using a CHIRALPAK^®^ AS-H (250 × 4.6 mm ID, 5 µM, Chiral Technologies Europe). The mobile phase was 100% ethanol. The flow rate was 0.25 mL/min at 30 °C and absorbance was measured at 240 nm as well as 350 nm, and 495 nm. Additional tested columns: CHIRALPAK^®^ AD-H (250 × 4.6 mm ID, 5 µM, Chiral Technologies Europe), CHIRALCEL^® O^D-H (250 × 4.6 mm ID, 5 µM, Chiral Technologies Europe) and MultoHigh Chiral-AM-HR (250 × 4.6 mm ID, 5 µM, Isera).

#### Chromatographic and mass spectral conditions

In total, 5 µL of sample were injected and analyzed with an Agilent 6520 QTOF equipped with a dual ESI ion source. Chromatographic separation was performed with a flow rate of 0.75 mL/min, temperature controlled at 30 °C in a column oven compartment with a NUCLEODUR Sphinx RP column (150 mm × 3 mm ID, 5 µM, Macherey-Nagel) coupled with a UNIVERSAL RP guard column (4 mm × 3 mm ID, Macherey-Nagel). The gradient was as follows: 0–1.5 min, 10% B; 1.5-22.5 min, 100% B; 22.5–30 min, 100% B; 30–30.1 min, 10% B; and 30.1–37.5 min, 10% B. Mobile phase A and mobile phase B consisted of water with 0.1% formic acid and acetonitrile with 0.1% formic acid, respectively. Reference mass correction was performed with constant infusion of purine and HP-0921 with 10 µL/min through the secondary nebulizer needle. Mass spectral analysis was performed in full-scan mode and in MS² mode. MS-Source parameters were as follows: Gas temperature, 250 °C; drying gas flow, 12 L/min; nebulizer pressure, 30 psi; capillary voltage, 3000 V, fragmentor voltage, 150 V; skimmer voltage, 65 V; octopole voltage, 750 V. Collision energy was set to 36 V, yielding overall best fragmentation efficacy. Additional measurements of intermediates of enzyme assays were conducted with a Waters ACQUITY I-class UPLC in combination with a Waters HSS T3 (C18) column (2.1 mm × 100 mm, 1.8 μm particle size) coupled to a Waters Acquity photo diode array detector (Waters) and a Waters Synapt G2-Si HDMS electrospray ionization (ESI)/quadrupole time-of-flight (Q-TOF) system. The following software was used for MS and HPLC data analysis: Agilent MassHunter Workstation Acquisition B.04.00, Agilent OpenLAB ChemStation Edition Rev. C.01.07, Agilent MassHunter Workstation Qualitative Analysis B.08.01, Agilent OpenLAB ChemStation Edition Rev. C.01.07, and Waters MassLynx V4.1.

#### Photometric analysis

The spectra were recorded on UV-1800PC (inside the anaerobic chamber) and UV-1650PC spectrophotometers (Shimadzu Corp.) with a 10-mm QS quartz cuvette (Hellma Analytics).

#### Flash chromatography

Silica gel chromatography was performed using silica gel from Macherey-Nagel (60, particle size 0.040–0.063 mm). The solvents for the mobile phase were chosen as described in the according sections.

#### Thin layer chromatography

TLC was performed using pre-coated TLC-sheets ALUGRAM^®^ Xtra SIL G/UV_254_ (Macherey-Nagel). The compounds were detected with UV light (254 nm and 366 nm).

#### Circular dichroism spectroscopy

CD spectroscopy of 7,8-dideoxy-6-oxo-griseorhodin C (**4**) and lenticulone (**11**) were recorded in a 0.2-mm path length cuvette at 20 °C with a Jasco J-810 spectropolarimeter (Jasco, Japan) equipped with a Peltier temperature controller PTC-423S (Jasco). The samples were dissolved in methanol, final concentrations: 0.6 mg/mL of compound **4** and 0.2 mg/mL of compound **11**. Instrumental parameters: spectral range, 180–700 nm, bandwidth, 1 nm, scanning speed, 500 nm/min. The CD spectra shown in Supplementary Figs. 32 and 50 represent the average of five accumulations after solvent subtraction.

#### IR spectroscopy and density functional theory calculations

Solid state IR spectra of compounds **4** and **12** were recorded on a Tensor 27 FTIR spectrometer (Bruker) equipped with an attenuated total reflection (ATR) cell (Pike instruments) at a resolution of 4 cm^−1^. To facilitate the assignment of characteristic vibrational modes, 11 conformers of **4** and 27 conformers of **12** were modeled using the conformer search algorithm in SPARTAN (Wavefunction, Inc.). To identify the most abundant conformers for each compound, geometry optimization, energy calculation, and frequency calculation on all unique conformers were performed at the DFT level (B3LYP/6-31 G(d)) in Gaussian 16 Rev. C.01 (Gaussian, Inc.). A conformer was identified with 99% abundance for **4** and a conformer with 97% abundance for compound **12** (calculated with respect to Δ*G*) that were used for comparison to the corresponding experimental spectra. IR bands were constructed with Lorentzian band shapes around each calculated intensity with a band width of 6 cm^−1^. Calculated frequencies were uniformly scaled by 0.97. Characteristic bands were assigned through visualization of vibrational modes in GaussView 6 (Gaussian Inc.).

#### NMR spectroscopy

^1^H- and ^13^C-NMR spectra of compounds **9** and **11** were recorded with an Avance III HD 400 instrument (Bruker) (^1^H: 400 MHz, ^13^C: 100 MHz). All other ^1^H- and ^13^C-NMR spectra were recorded with a FT-NMR DMX 600 instrument (Bruker) (^1^H: 600 MHz, ^13^C: 150 MHz). DMSO, CDCl_3_ and 1,4-dioxane-d_8_ were used as solvents. Chemical shifts refer to the *δ*-scale and are reported in ppm with a solvent resonance as an internal standard (CHCl_3_: *δ*_H_ 7.26; CDCl_3_: *δ*_C_ 77.16, DMSO: *δ*_H_ 2.50, *δ*_C_ 39.51, 1,4-dioxane-d_8_: *δ*_H_ 3.53). Data was analyzed and processed using the Bruker TopSpin 4.0.7 software.

#### Chemical structures and figures

Chemical structures for figures were drawn with PerkinElmer ChemDraw 19.0. UV and MS traces of enzymatic assays were plotted using GraphPaD Prism 8.3.0.

### Reporting summary

Further information on research design is available in the Nature Research Reporting Summary linked to this article.

## Supplementary information

Supplementary Information

Reporting Summary

## Data Availability

The authors declare that the main data supporting the findings of this study are available within the article and its Supplementary Information files. Extra data are available from the corresponding author upon request.
