# Supplementary material for: Enzymatic spiroketal formation via oxidative rearrangement of pentangular polyketides
Source: Nat Commun. 2021 Mar 4;12:1431. doi: 10.1038/s41467-021-21432-9 (PMC7933358; doi:10.1038/s41467-021-21432-9)
Supplement: Supplementary file 1 — Supplementary Information [file 41467_2021_21432_MOESM1_ESM.docx]

**Supplementary Information**

**Enzymatic spiroketal formation via oxidative rearrangement of pentangular polyketides**

**Authors:** Britta Frensch^1^, Thorsten Lechtenberg^1^, Michel Kather^2^, Zeynep Yunt^3^, Martin Betschart^4^, Bernd Kammerer^2,5^, Steffen Lüdeke^4^, Michael Müller^4^, Jörn Piel^6^, Robin Teufel^1^*

**Affiliations:**

^1^Faculty of Biology, University of Freiburg, Schänzlestrasse 1, 79104 Freiburg, Germany.

^2^BIOSS Center for Biological Signaling Studies, University of Freiburg, Schänzlestrasse 18, 79104 Freiburg, Germany.

^3^Department of Molecular Biology and Genetics, Koç University, Istanbul, 34450, Turkey.

^4^Institute of Pharmaceutical Sciences, University of Freiburg, Albertstrasse 25, 79104 Freiburg, Germany.

^5^Hermann Staudinger Graduate School, University of Freiburg, Hebelstrasse 27, 79104 Freiburg, Germany.

^6^Institute of Microbiology, Eidgenössische Technische Hochschule (ETH) Zürich, Vladimir-Prelog-Weg 4, 8093 Zürich, Switzerland.

*Correspondence to: [robin.teufel@zbsa.uni-freiburg.de](mailto:robin.teufel@zbsa.uni-freiburg.de)

**Supplementary Figure 1**. Heterologous production of GrhO5. **A**) SDS-PAGE analysis of MBP-tagged GrhO5 (98 kDa) purified by affinity chromatography. Pierce^TM^ unstained protein MW marker (Thermo Fisher Scientific) was applied as reference. **B**) RP-HPLC analysis (chromatograms at 254 nm) to identify the cofactor of GrhO5 (following denaturation with methanol and cofactor isolation) as flavin adenine dinucleotide (FAD). The two lower chromatograms show FAD and flavin mononucleotide (FMN) as reference. n=3 independent samples of the cofactor of GrhO5 were extracted and analyzed (a representative example is shown).

**Supplementary Figure 2**. Determination of the kinetic parameters of GrhO5 and GrhO6. Enzyme kinetics were determined spectrophotometrically at wavelengths of 556 and 536 nm for collinone (**3**) and lenticulone (**11**), respectively. Turnover rates (k_cat_) were calculated based on extinction coefficients of 4850 l×mol^-1^×cm^-1^ at 556 nm (**3**) and 1700 l×mol^-1^×cm^-1^ at 536 nm (**11**), where only the oxidized substrates absorb light (see Supplementary Fig. 29). **A**) Michaelis-Menten curve for the GrhO5-catalyzed **3** reduction with a K_M_ value of 181 ± 24 µM and a k_cat_ of 6 ± 1 s^-1^. NADPH (1.5 mM) was used as electron donor. **B**) The enzyme activity with NADH was around 20% compared to NADPH (at a concentration of 1.5 mM each) and ca. 0.4 mM **3**. **C**) Comparison of the GrhO5-dependent **3** reduction with the much slower non-enzymatic reduction. NADPH (1.5 mM) was used as electron donor and free FAD was used at the same concentration (1 µM) as GrhO5. **D**) Michaelis-Menten curve for the GrhO6-catalyzed **11** reduction with a K_M_ value of 23 ± 6 µM and a k_cat_ of 0.4 ± 0.03 s^-1^. NADPH (1.5 mM) was used as electron donor. **E**) The enzyme activity with NADH was around 40% compared to NADPH (at a concentration of 1.5 mM each) with ca. 50 µM **11**. For each tested condition n=3 independent replicates were analyzed and error bars represent the standard error of mean. The proper determination of the stoichiometry of NADPH consumption and product formation for GrhO5 and GrhO6 proved unfeasible due to the spontaneous (re)oxidation of the intermediates **5** and **10** to **3** and **11**, respectively (that are then reduced again by GrhO5/GrhO6 which accordingly increases NADPH demand). Also, the final products can undergo reduction/oxidation cycles, which further increases NADPH consumption. In the case of GrhO5, e.g., the consumption of 4-5 NADPH per collinone was observed.

**Supplementary Figure 3**. Reduction of collinone (**3**) produces dihydrocollinone (**5**). **A**) RP-HPLC chromatograms at 254 nm show the enzymatic reduction with GrhO5 as well as the non-enzymatic reductions with titanium(III)citrate or dithiothreitol (DTT). **B**) LC-HRMS showing compound **3** measured in positive ion mode (calc. [M+H]^+^ 535.087, found 535.088) and compound **5** measured in negative ion mode (calc. [M-H]^-^ 535.088, found 535.088). The measurement of dihydrocollinone (**5**) was performed without the column via direct injection into the ion source because otherwise **5** completely oxidized to **3**. **C**) Comparison of the UV-Vis spectra of both compounds. The two maxima between 400 and 500 nm in the spectrum of **5** are characteristic for reduced compounds with hydronaphthoquinone moieties. For all assays, at least n=3 independent replicates were analyzed and representative examples are shown.


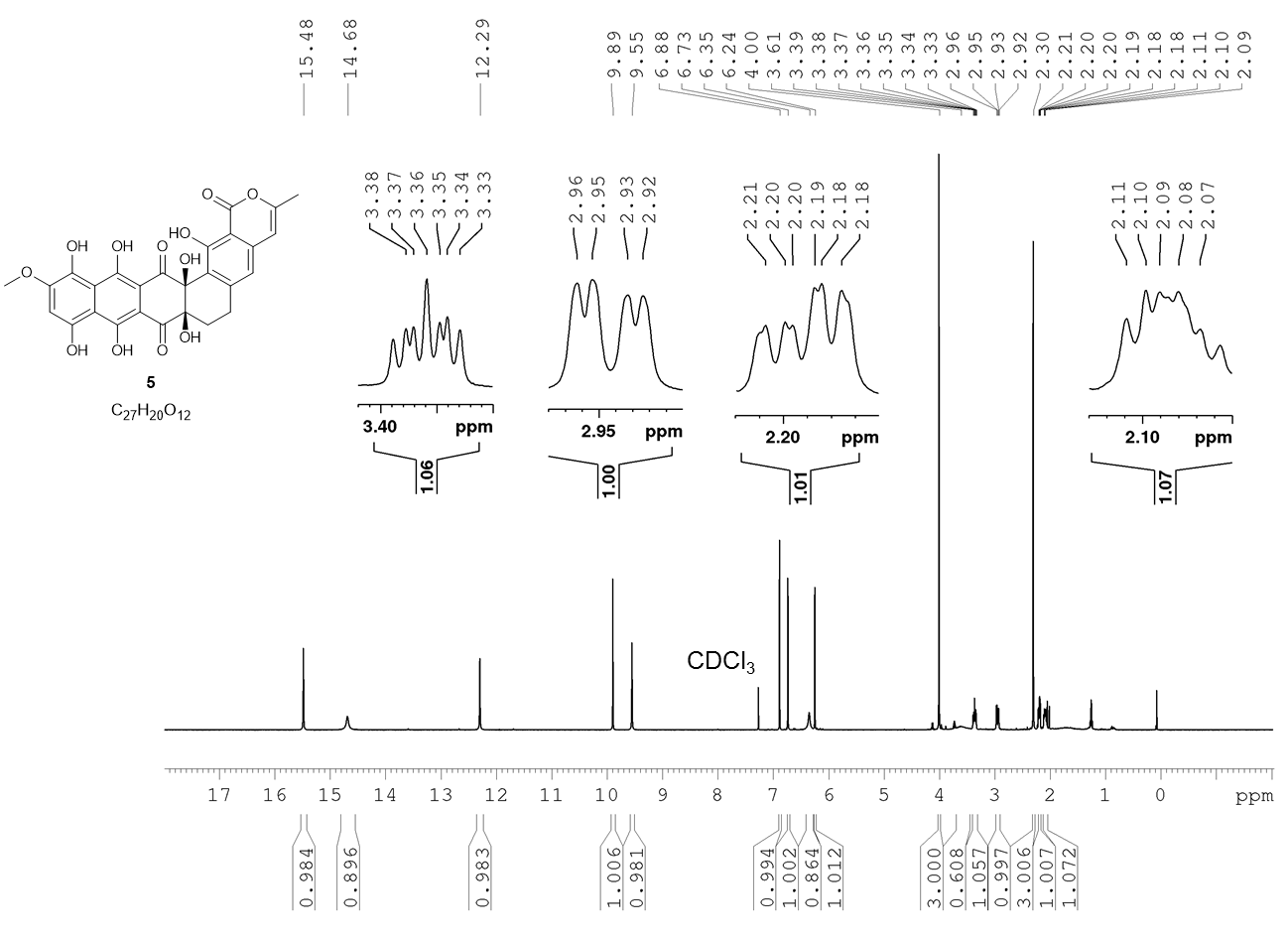


**Supplementary Figure 4.** ^1^H NMR spectrum of dihydrocollinone (**5**) (CDCl_3_, 600 MHz).


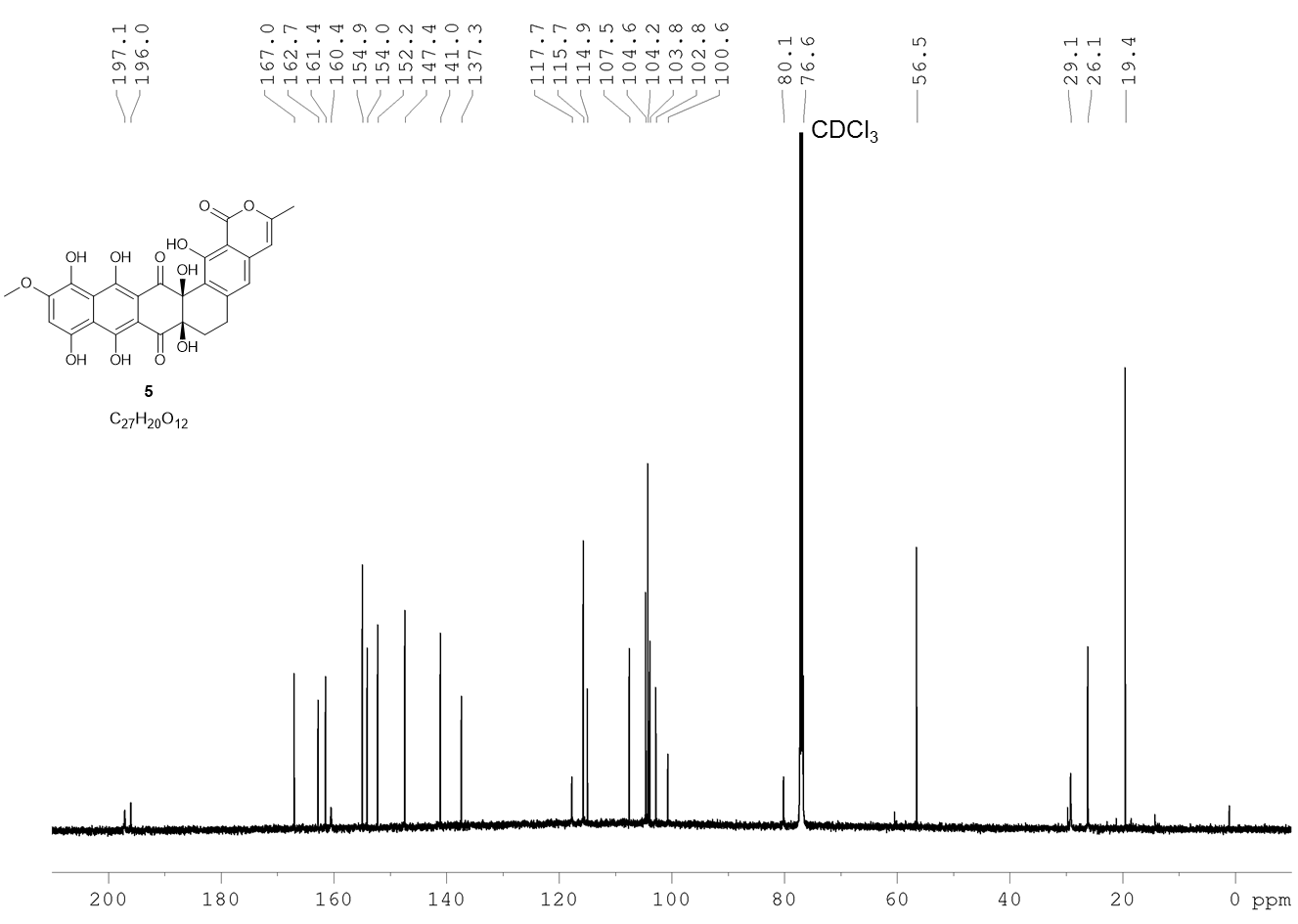


**Supplementary Figure 5**. ^13^C NMR spectrum of dihydrocollinone (**5**) (CDCl_3_, 150 MHz).


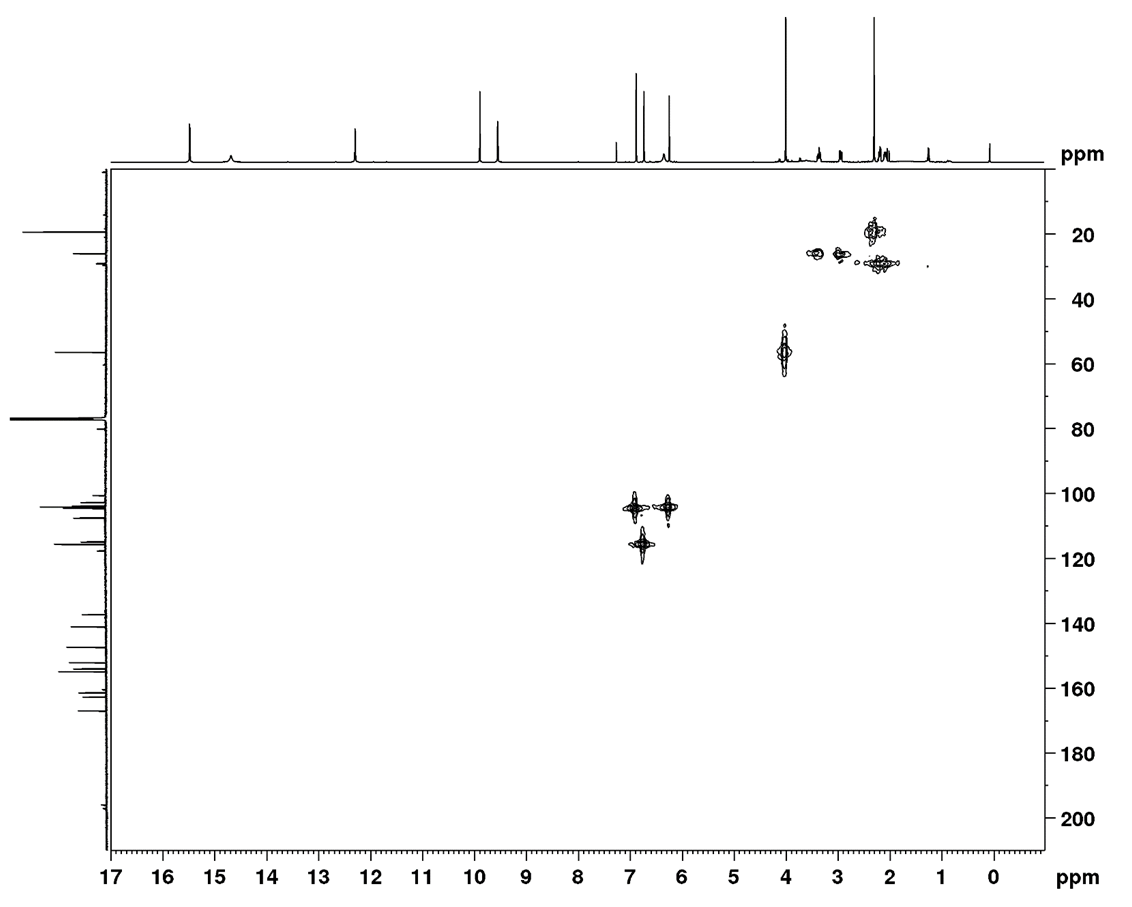


**Supplementary Figure 6**. HSQC NMR spectrum of dihydrocollinone (**5**) (CDCl_3_, 600 MHz).

**
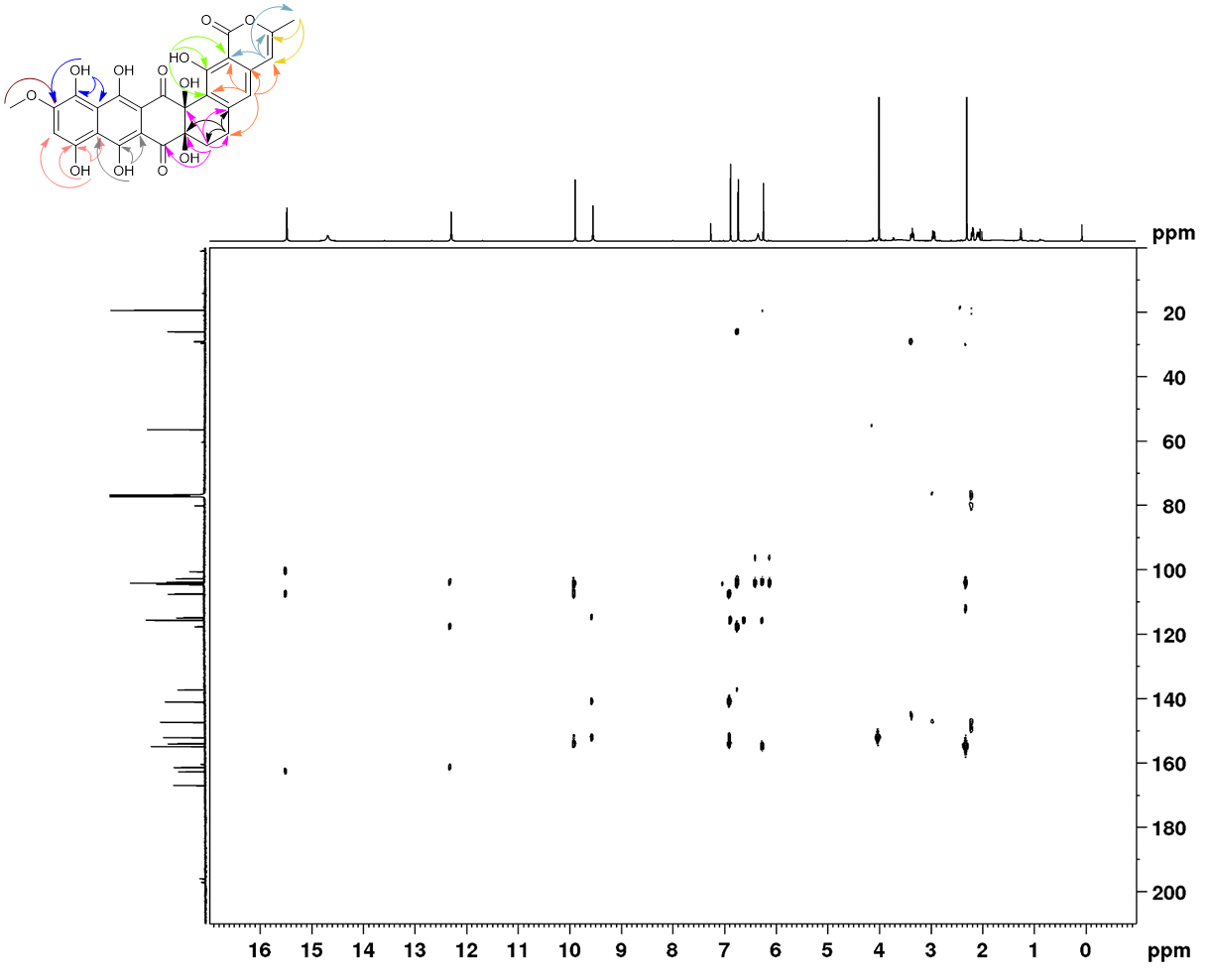
**

**Supplementary Figure 7.** HMBC NMR spectrum of dihydrocollinone (**5**) (CDCl_3_, 600 MHz). The arrows indicate the HMBC correlations of each proton.

**Supplementary Figure 8**. Uncropped RP-HPLC traces for enzyme assays with substrate **3** in presence of O_2_ at λ = 254 nm. See Fig. 2 for full legend. The peaks between 2 and 9 min are residual NAD(P)H and/or NAD(P)^+^ and their degradation products. The peak at 16.8 min appeared only sporadically in assays lacking GrhO1 (e.g., this compound was not observed in other GrhO5 and RubL assays shown in Supplementary Fig. 52), which likely represents another minor shunt product that was not further characterized. For all assays, at least n=3 independent replicates were conducted and representative examples are shown.

**Supplementary Figure 9**. Methylation of secocollinone (**7**) to the stable derivative **6**. Introduced methyl groups are highlighted in the structure at the bottom. **A**) RP-HPLC analysis showing the time dependent formation of secocollinone after the derivatization with DMS of samples from an enzymatic assay. The maximum amount was obtained after 3 min. **B**) The derivatization reaction of the extract of the deletion mutant *S. albus* KR5 (Δ*grhO1*) analyzed after silica gel chromatography via RP-HPLC at 254 nm shows **6** at 15.6 min. **C**) Extracted ion chromatogram (EIC) of compound **6** ([M+H]^+^ = 635.21) and **D**) UV-Vis spectrum of **6**. The experiment was repeated n=2 times independently.


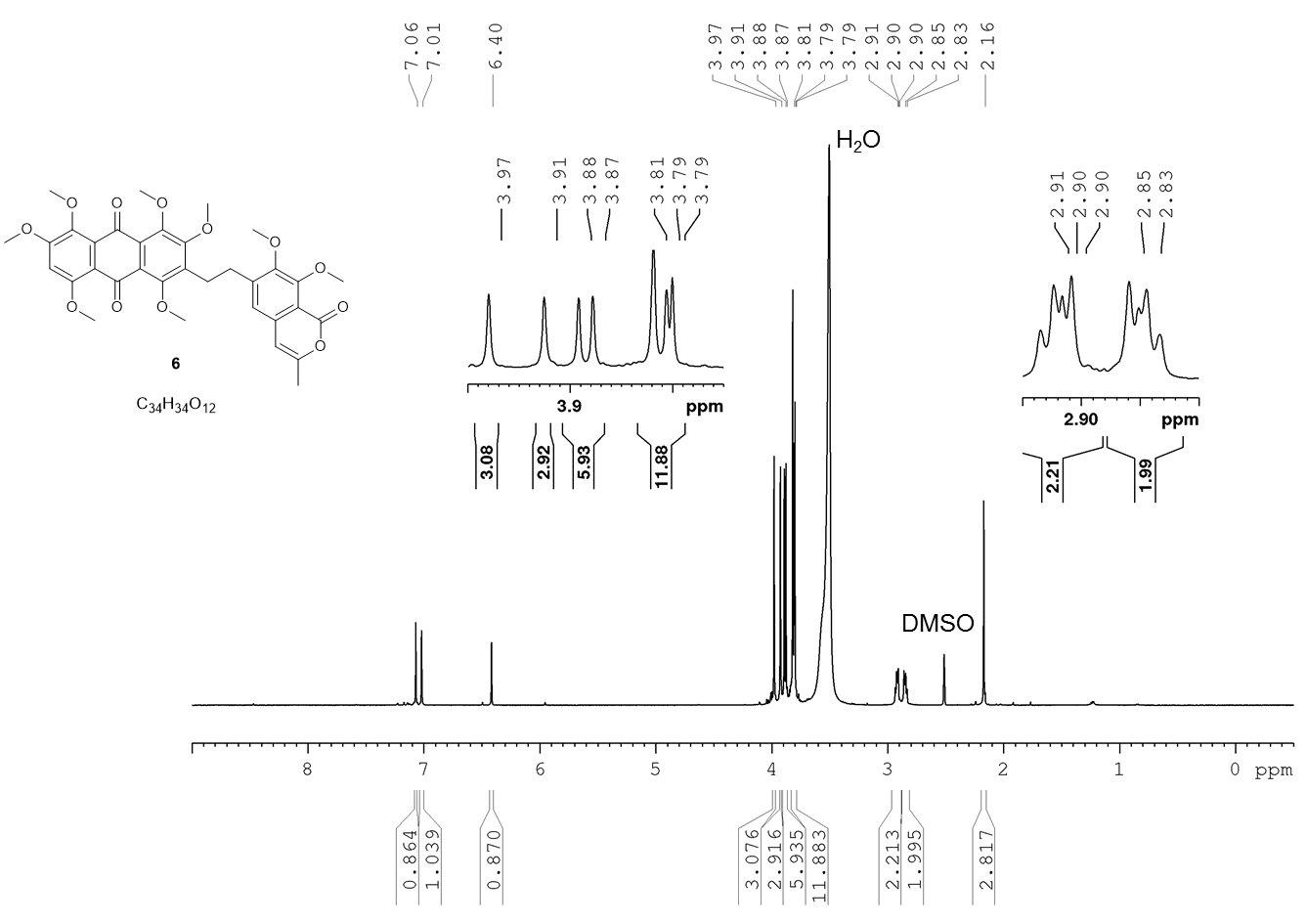


**Supplementary Figure 10.** ^1^H NMR spectrum of compound **6** (DMSO-d_6_, 600 MHz).

**
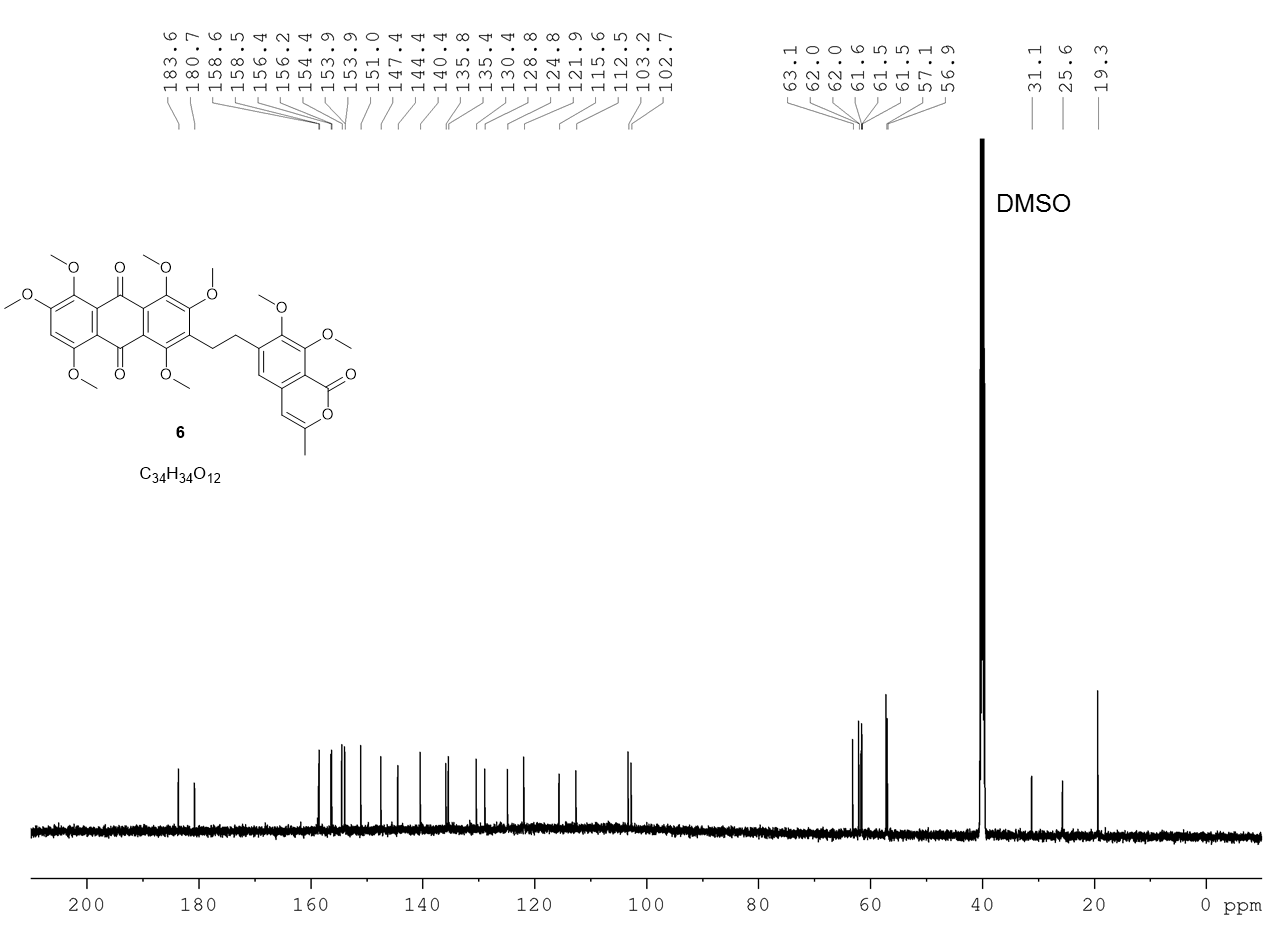
**

**Supplementary Figure 11**. ^13^C NMR spectrum of compound **6** (DMSO-d_6_, 150 MHz).

**
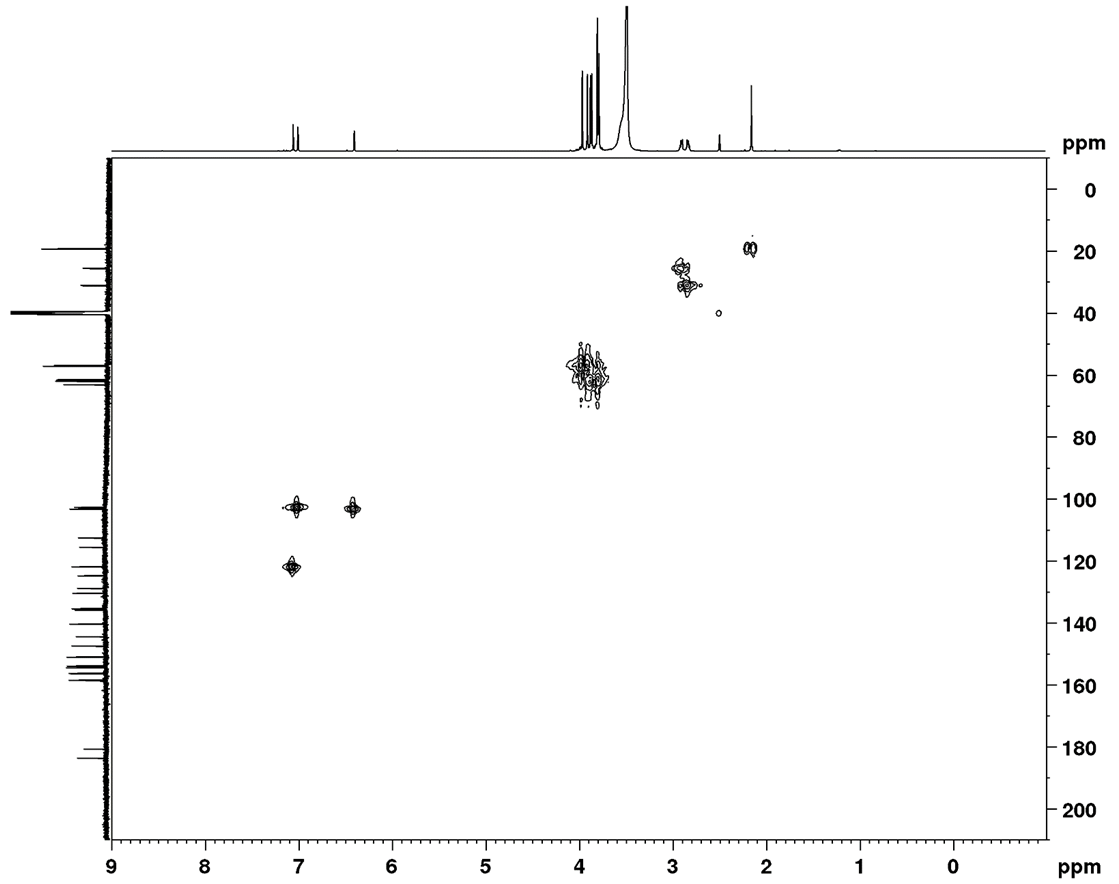
**

**Supplementary Figure 12**. HSQC NMR spectrum of compound **6** (DMSO-d_6_, 600 MHz).

**
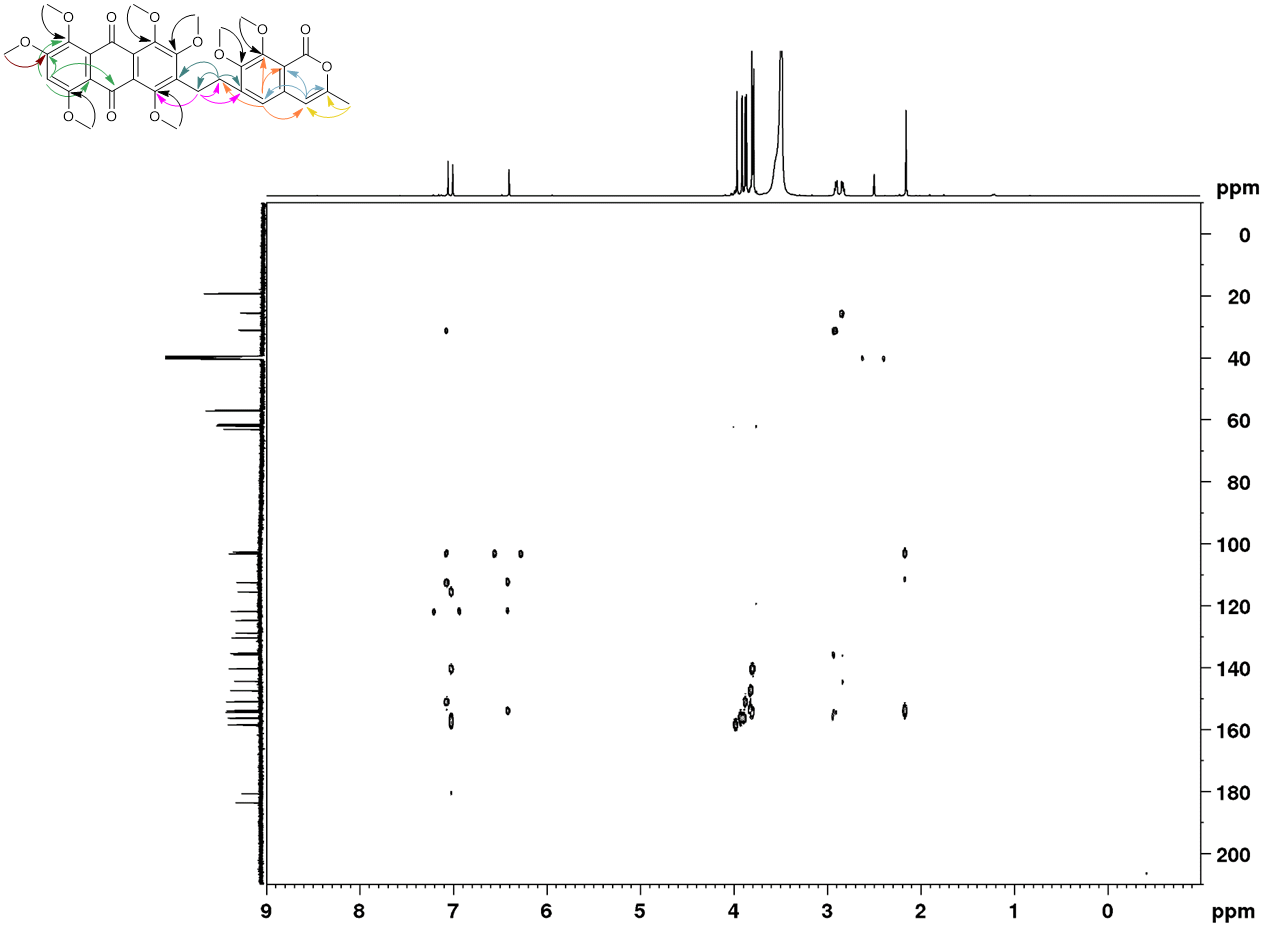
**

**Supplementary Figure 13**. HMBC NMR spectrum of compound **6** (DMSO-d_6_, 600 MHz). The arrows indicate the HMBC correlations of each proton.

**Supplementary Figure 14**. Spontaneous conversion of isolated compound **8**. **A**) HPLC chromatogram (gradient from the LC-HRMS method was used, which accordingly results in different retention times compared to other HPLC runs shown, e.g., in Fig. 2) at 254 nm shows that isolated compound **8** decomposes into compound **9** and dihydrolenticulone (**10**) (among other non-characterized compounds) during the removal of the solvent under reduced pressure. Compound **10** directly autooxidizes to **11** during the measurement. **B**) LC-HRMS analysis of compound **8** (calc. for C_27_H_20_O_14_ [M-H]^-^ 567.078, found 567.077). The analysis in the middle shows the mass of the spontaneously formed **9** (calc. for C_26_H_18_O_12_ [M-H]^-^ 521.073, found 521.074). LC-HRMS analysis on the right side shows both spontaneously formed dihydrolenticulone (**10**) (calc. for C_26_H_18_O_12_ [M-H]^-^ 521.073, found 521.072) and the oxidized form lenticulone (**11**) (calc. for C_26_H_16_O_12_ [M-H]^-^ 519.057, found 519.057). The formed compounds were further identified on the basis of characteristic UV-Vis spectra and retention time. At least n=3 independent replicates were analyzed and representative examples are shown.

**Supplementary Figure 15**. Autooxidation of reduced lenticulone (purified by HPLC). **A**) HPLC analysis (chromatogram at 254 nm) of purified dihydrolenticulone (**10**) ([M+H]^+^ = 523.09) showing the autooxidation to **11** ([M+H]^+^ = 521.07). **B**) EIC’s for masses corresponding to **10** (bottom) and **11** (top). The upper EIC trace demonstrates the autooxidation of **10** to **11** during the measurement, as ions with [M+H]^+^ = 521.07 (**11**) are also observed at the acquisition time of **10**. At least n=3 independent replicates were performed with similar results.

**Supplementary Figure 16**. Identification of compound **11** as the previously reported lenticulone. **A**) LC-HRMS analysis of **11** (calc. for C_26_H_16_O_12_ [M+H]^+^ 521.072, found 521.070). **B**) UV-Vis spectrum of **11**. **C**) RP-HPLC analysis at 254 nm showing the comparison of purified lenticulone (**11**) (produced by an *in vitro* GrhO5 assay) with a GrhO5 assay quenched after 10 min and an extract of the deletion mutant *S. albus* KR42 (Δ*grhJ*), from which **11** was first isolated and characterized^1^. Retention times, UV-Vis, MS and MS^2^ spectra were identical. For all assays and extractions, at least n=3 independent replicates were analyzed.

**Supplementary Figure 17**. Reduction of lenticulone (**11**, grey trace) to dihydrolenticulone (**10**) under anaerobic conditions. Compound **11** was readily reduced by 1 mM Ti(III)citrate as well as by 5 µM GrhO6 or 5 µM GrhO5 with 1.5 mM NADH and NADPH, as shown by the distinct changes of the UV-Vis spectrum of the compound. For all assays, at least n=3 independent replicates were analyzed and representative examples are shown.

**
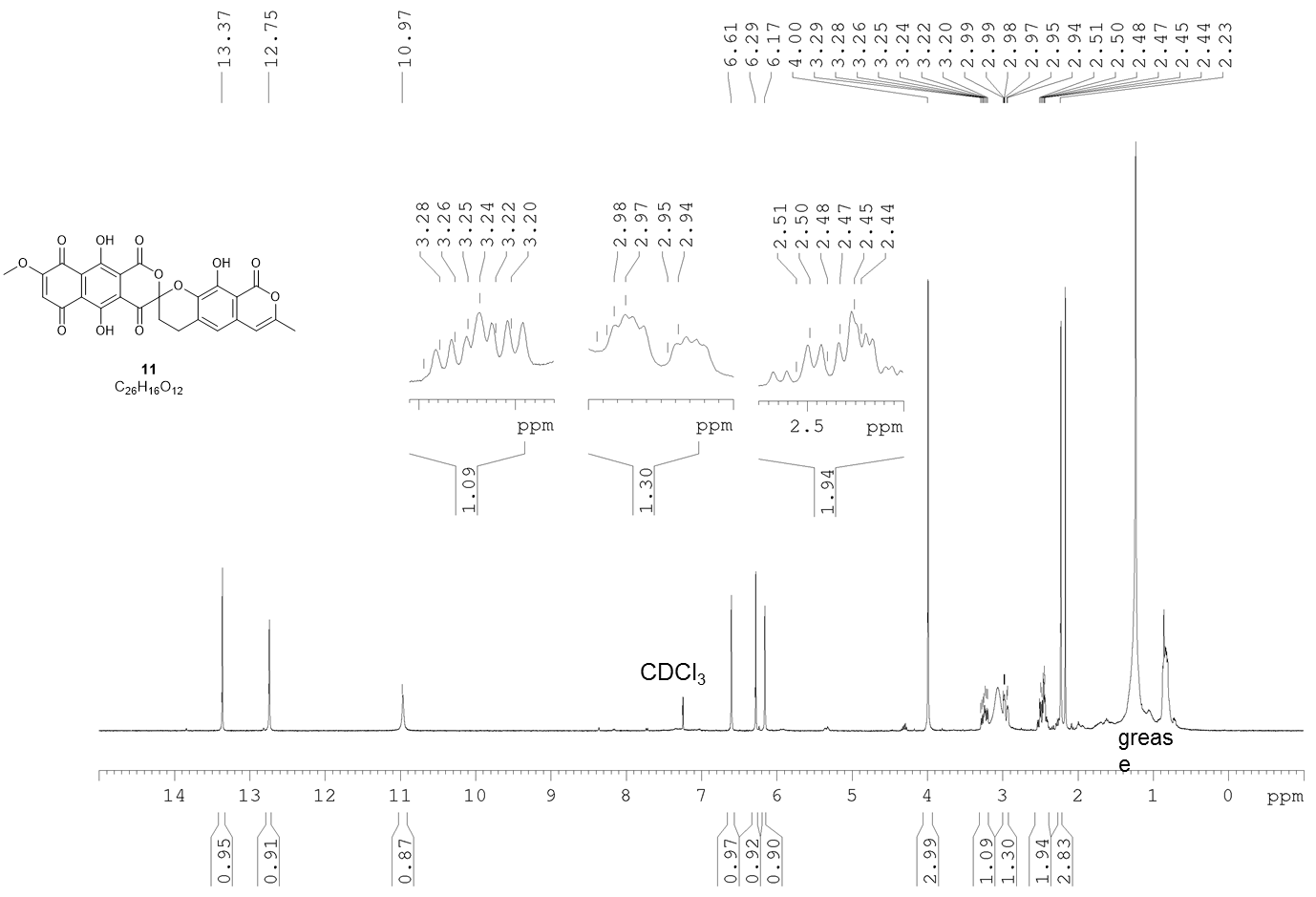
**

**Supplementary Figure 18**. ^1^H NMR spectrum of lenticulone (**11**) (CDCl_3_, 400 MHz).

**
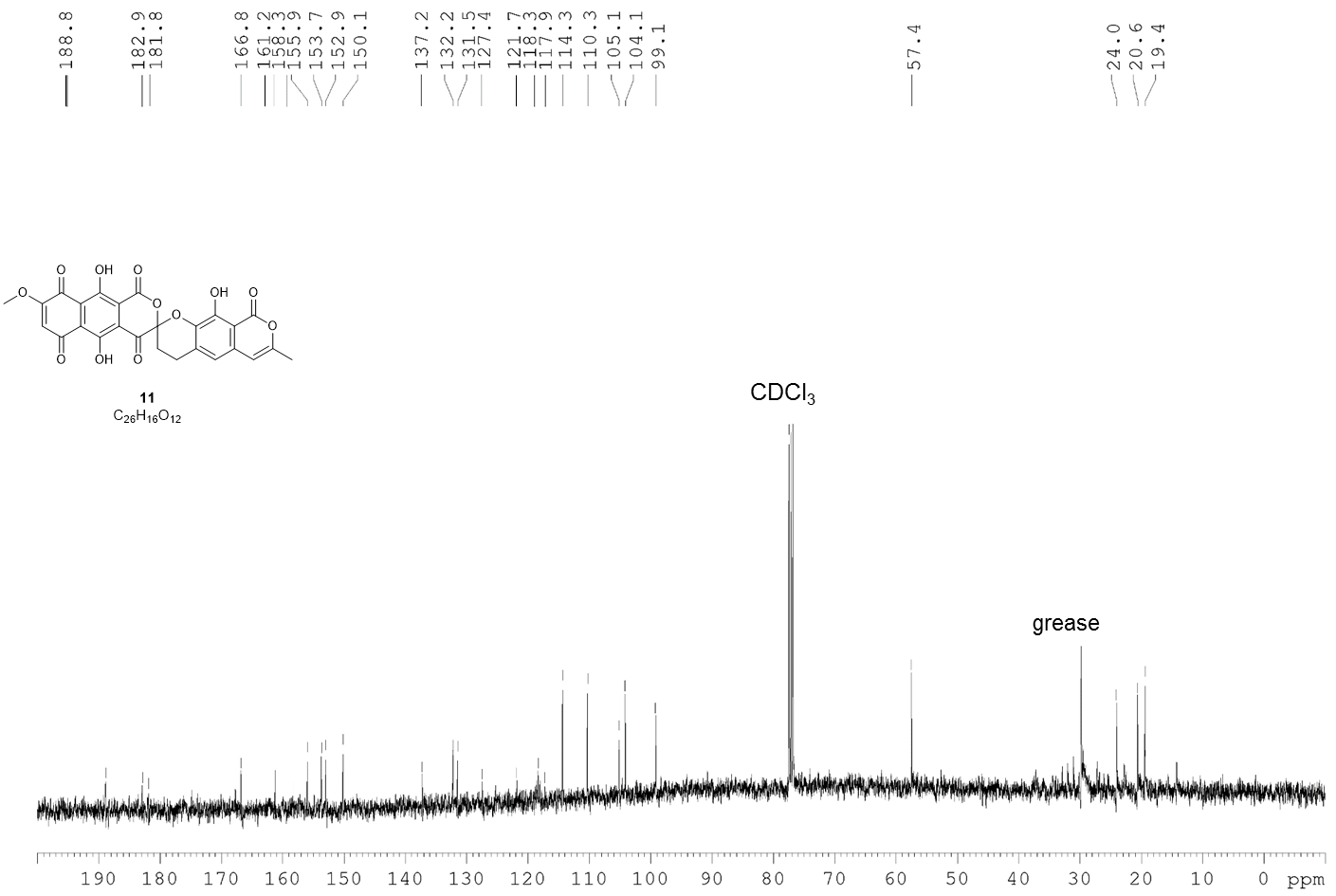
**

**Supplementary Figure 19**. ^13^C NMR spectrum of lenticulone (**11**) (CDCl_3_, 100 MHz).

**
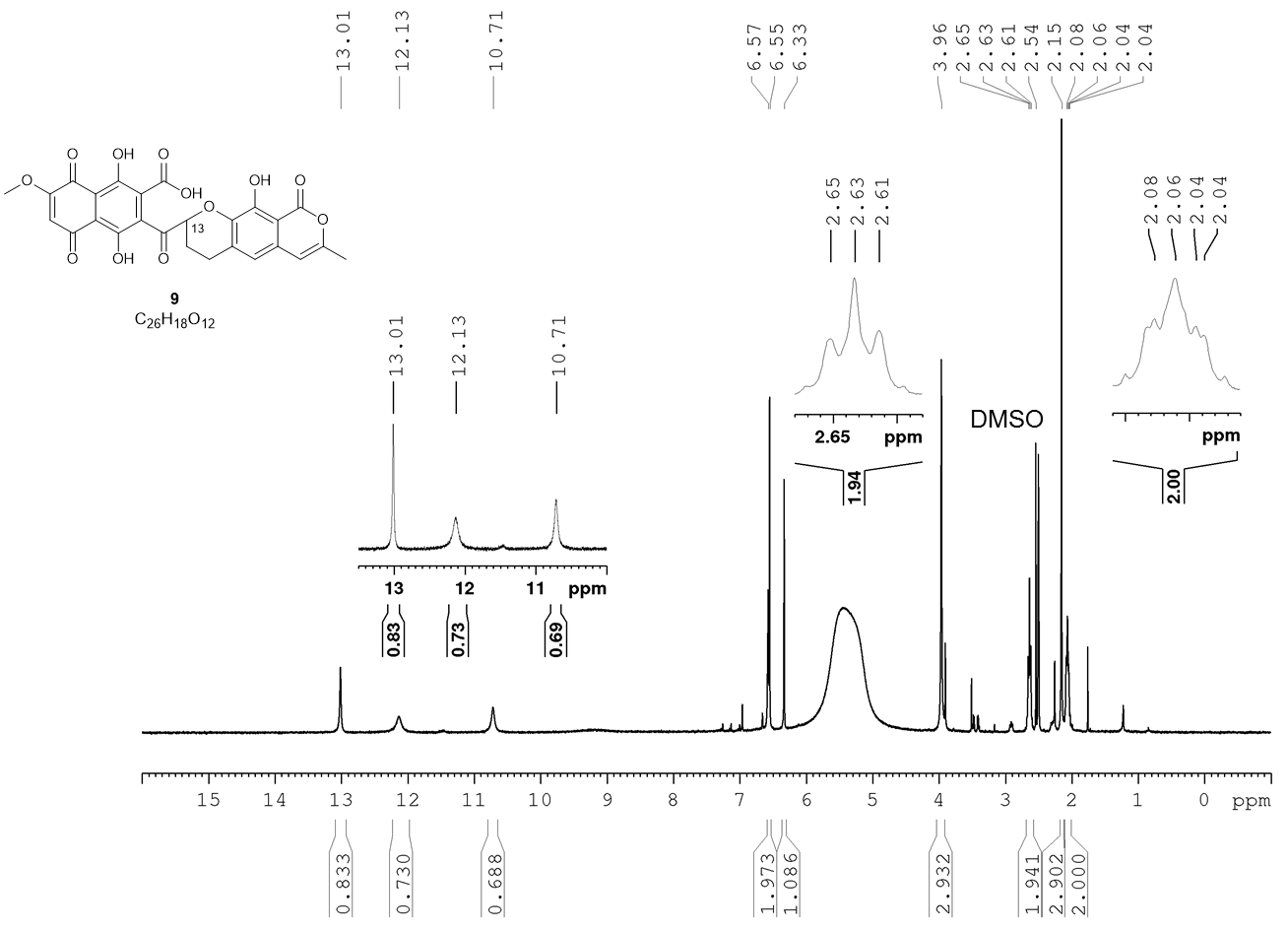
**

**Supplementary Figure 20**. ^1^H NMR spectrum of compound **9** (DMSO-d_6_/1% TFA-d, 400 MHz). The proton at C13 is not detectable.


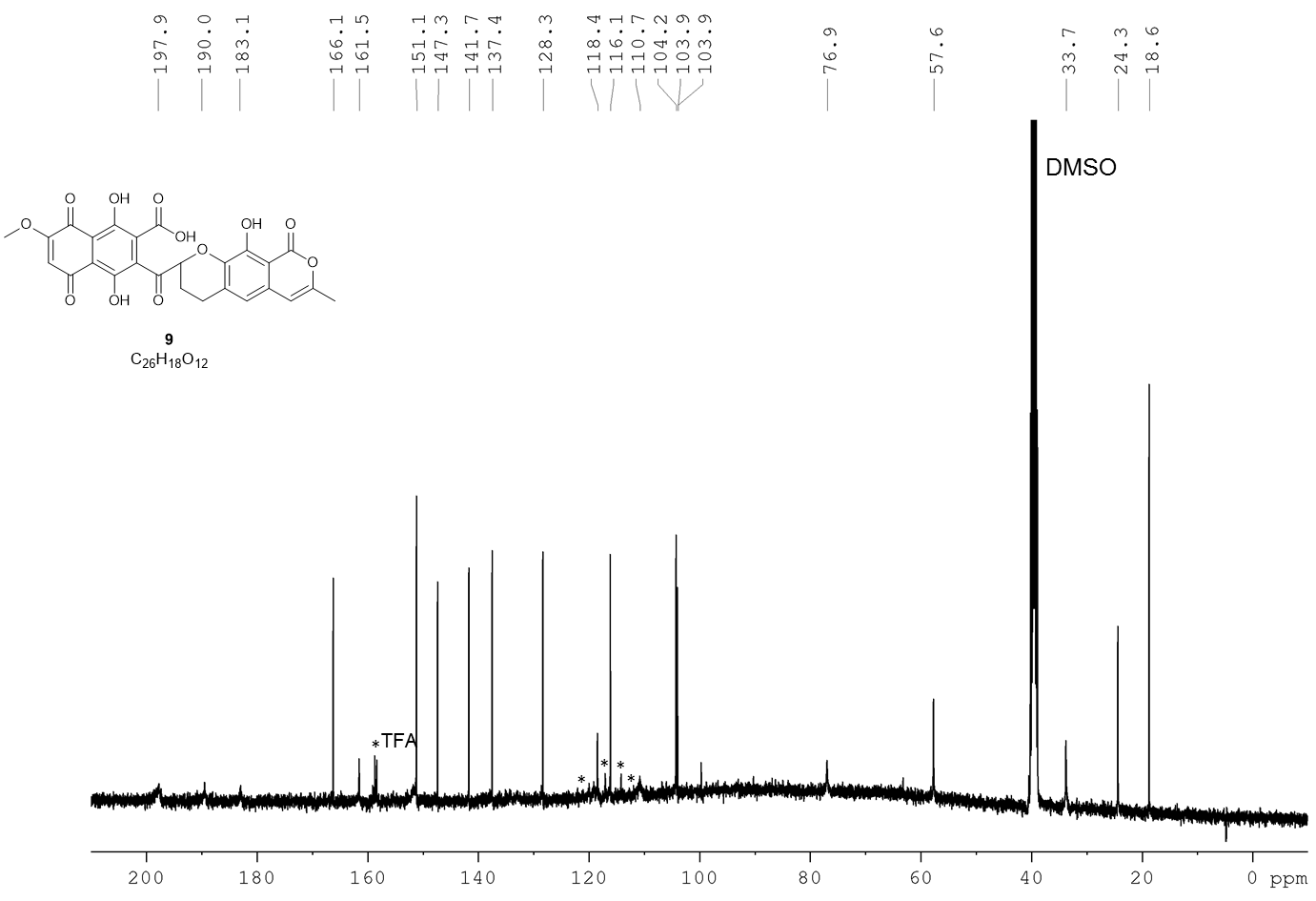


**Supplementary Figure 21.** ^13^C NMR spectrum of compound **9** (DMSO-d_6_/1% TFA-d, 100 MHz). Peaks with an asterisk belong to TFA.


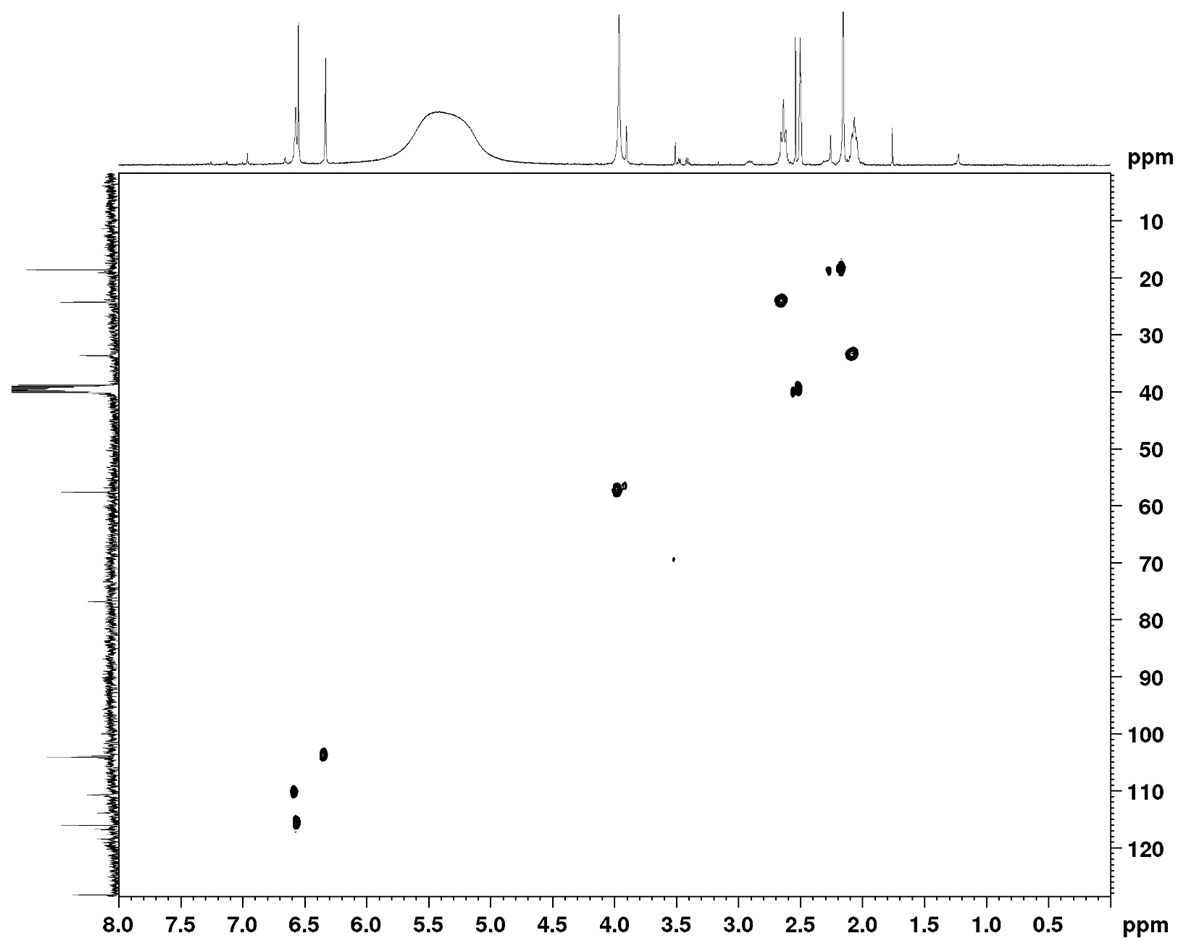


**Supplementary Figure 22.** HSQC NMR spectrum of compound **9** (DMSO-d_6_/1% TFA-d, 400 MHz).


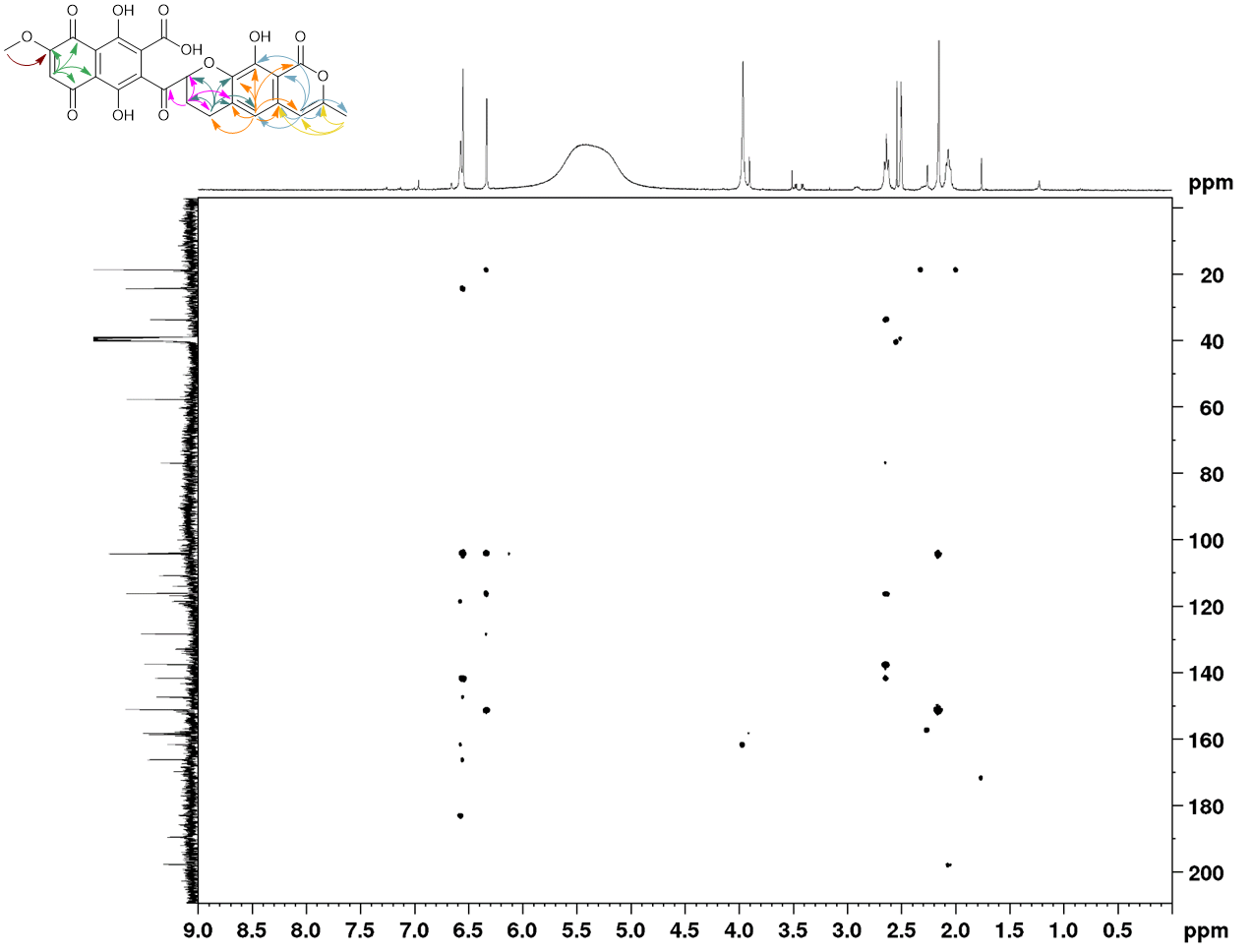


**Supplementary Figure 23.** HMBC NMR spectrum of compound **9** (DMSO-d_6_/1% TFA-d, 400 MHz). The arrows indicate the HMBC correlations of each proton.

**
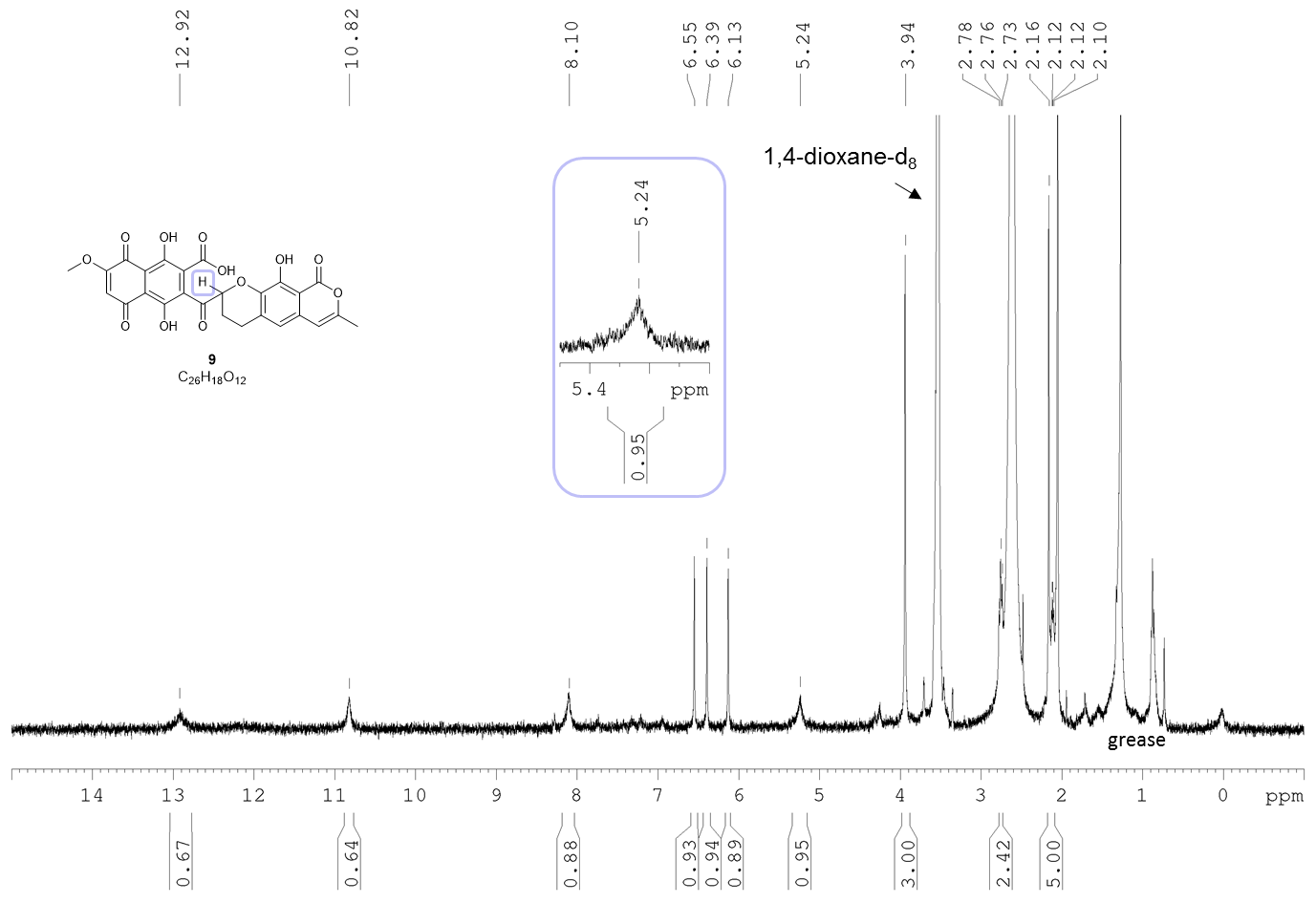
**

**Supplementary Figure 24**. ^1^H NMR spectrum of compound **9** (1,4-dioxane-d_8_, 400 MHz).

**Supplementary Figure 25.** MS/MS fragmentation pathway and characteristic fragments of compound **9**. For more fragments, see Supplementary Table 1. For shown MS experiments, at least n=3 independent replicates were analyzed (representative examples are shown).

**Supplementary Figure 26**. Isotope labeling assay of compound **8** produced by GrhO5 in presence of 55% H_2_^18^O (*v*/*v*) that was purified by RP-HPLC and then analyzed by LC-HRMS. The incubation time with labeled water was only 3 min, before organic extraction of the assay and direct injection of the EtOAc dissolved **8** onto the HPLC for purification, to avoid complete degradation of **8** for subsequent analysis by LCMS. **A**) EIC’s for masses corresponding to compound **8** ([M-H]^-^ = 567.08), shunt product **9** ([M-H]^-^ = 521.07) and lenticulone (**11**) ([M-H]^-^ = 519.06). Only low ion counts were measured for unstable compound **8**, which largely decomposed to **9** and **11** during the removal of the solvent under reduced pressure (see also Supplementary Fig. 14). Compounds **9** and **11** were further identified on the basis of characteristic UV-Vis spectra and retention time. **B**) Isotopic distribution of **8**, **9**, and **11**. The results show the incorporation of a single ^18^O-atom (21%) as well as two ^18^O-atoms (14%) in compound **8**. In contrast, both **9** and **11** retain only a single ^18^O-atom (13 and 27%), confirming the elimination of one H_2_^18^O-derived ^18^O by decarboxylation (see also Fig. 3). The experiment was repeated n=2 times independently with similar results.

**Supplementary Figure 27**. Isotope labeling assay of compound **9** produced by GrhO5 in presence of 97% ^18^O_2_ gas or 56% H_2_^18^O (*v*/*v*). **A**) LC-HRMS analysis of the isotope labeling assays with GrhO5. When H_2_^18^O was used, the incubation time was 5 min. The results show the incorporation of a single ^18^O-atom (39%). The small amounts of a second ^18^O-atom (6%) likely resulted from the spontaneous exchange with water. When ^18^O_2_ was added to the assays, 77% of the **9** had a single ^18^O-atom incorporated. **B**) Cutout of the MS/MS fragmentation pattern of the labeled compound **9** to show the incorporation of the ^18^O-atoms in the western and eastern half of the molecule. The fragments with m/z of [M+H]^+^ = 321.041 and [M+H]^+^ = 333.048 show the incorporation of (H_2_^18^O-derived) ^18^O in the western molecule half. The two fragments with m/z of [M+H]^+^ = 207.053 and [M+H]^+^ = 233.066 show the incorporation of (^18^O_2_-derived) ^18^O in the eastern molecule half (see Supplementary Figures 25 & 28 and Supplementary Tables 1-3). For all assays, at least n=3 independent replicates were conducted and representative examples are shown.

**Supplementary Figure 28**. Isotope labeling assay of lenticulone (**11**). Compound **10** produced by GrhO5 in presence of 97% ^18^O_2_ gas or 56% H_2_^18^O (*v*/*v*), followed by the autooxidation to **11**. **A**) LC-HRMS analysis of the labeling assays with GrhO5. When labeled water was used, the incubation time was 1 or 5 min. The results show the incorporation of a single ^18^O-atom (43%). The small amounts of a second ^18^O-atom (15%) likely resulted from the spontaneous exchange of ketones with water. When ^18^O_2_ was added, 79% of **11** had a single ^18^O-atom incorporated. **B**) Cutout of the MS/MS fragmentation pattern of the labeled compound **11** to show the incorporation of the ^18^O-atoms in the western and eastern half of the molecule. The fragments with m/z of [M+H]^+^ = 249.029 and [M+H]^+^ = 277.017 show the incorporation of (H_2_^18^O-derived) ^18^O in the western molecule half. The two fragments with m/z of [M+H]^+^ = 207.050 and [M+H]^+^ = 249.036 show the incorporation of (^18^O_2_-derived) ^18^O in the eastern molecule half. For more fragments see Supplementary Table 4 and 5. For all assays, at least n=3 independent replicates were conducted and representative examples are shown.

**Supplementary Figure 29**. Comparison of the UV-Vis spectra of the reduced and corresponding oxidized compounds. **A**) UV-Vis spectra of **5**, **8** and **10** featuring the double absorption peak between 400 and 500 nm that is characteristic for the naphthohydroquinone moiety. **B**) The UV-Vis spectra of compounds **3**, **9** and **11** instead shows a single, flatter absorption peak in this range, which is indicative of an oxidized A ring.

**Supplementary Figure 30**. Heterologous production of GrhO1. **A**) SDS-PAGE analysis of His_8_-tagged GrhO1 (52 kDa) after size exclusion chromatography. Pierce^TM^ unstained protein MW marker (Thermo Fisher Scientific) was used as reference. **B**) Size exclusion chromatography of GrhO1. The enzyme was purified successfully and peak fractions (highlighted in yellow) were used for enzymatic assays. For the purification, at least n=3 independent replicates were performed (representative example is shown).

**Supplementary Figure 31**. RP-HPLC analysis (chromatograms at 254 nm) showing that purified compound **9** could not be further converted by GrhO1 and GrhO5 (GrhO6 also does not convert **9**, see Supplementary Figure 49). The bottom trace shows purified shunt product **9** that spontaneously decomposes to several compounds (retention times between 8.5 – 10 min). For all assays, n=3 independent replicates were conducted and analyzed (representative examples are shown).

**Supplementary Figure 32**. Racemic mixture of lenticulone (**11**). **A**) Possible mechanism for spontaneous racemization via ring-opening and recyclization. **B**) UV circular dichroism (CD) spectrum of lenticulone (**11**) showing that the enzymatically formed compound **11** is present as racemic mixture. **C**) Chiral HPLC analysis at 350 nm showing no separation of racemic **11**, probably as result of the rapid spontaneous racemization (several columns were tested and non resulted in proper separation, see methods).

**Supplementary Figure 33**. Heterologous production of GrhO6. **A**) SDS-PAGE analysis of His_6_-tagged GrhO6 (60 kDa) after Ni^2+^ affinity chromatography. Pierce^TM^ unstained protein MW marker (Thermo Fisher Scientific) was applied as reference. **B**) HPLC analysis (chromatograms at 254 nm) identified the cofactor of GrhO6 (following denaturation with methanol and cofactor isolation) as FAD. The two lower chromatograms show FAD and FMN as reference. At least n=3 independent samples of the cofactor of GrhO6 were extracted and analyzed (a representative example is shown).

**Supplementary Figure 34**. UV-Vis spectra and LC-HRMS analysis of compounds **4** and **12**. **A**) UV-Vis spectrum and LC-HRMS analysis of 7,8-dideoxy-6-oxo-griseorhodin C (**4**) (calc. [M+H]^+^ 493.077, found 493.076). **B**) UV-Vis spectrum of compound **12**. LC-HRMS measurement was performed with a lower fragmentor voltage (100 V) to avoid excessive in-source fragmentation of the mother ion 511.087 to 493.077. The LC-HRMS analysis shows compound **12** (calc. for C_25_H_18_O_12_ [M+H]^+^ 511.087, found 511.087, [**4**+H_2_O]) and the fragment with m/z of [M+H]^+^ = 493.077 (calc. 493.077) after the loss of a water molecule. For both compounds, at least n=3 independent measurements were performed by MS.

**Supplementary Figure 35**. LC-HRMS and UV-Vis spectroscopic analysis of 7,8-dideoxygriseorhodin C (**13**). **A**) Shown is the RP-HPLC trace at 254 nm of purified **13** from the deletion mutant *S. albus* KR7 (Δ*grhO7*). **B**) UV-Vis spectrum of **13**. **C**) LC-HRMS analysis of **13** (calc. [M+H]^+^ 495.092, found 495.092). For the compound, at least n=3 independent measurements were performed.


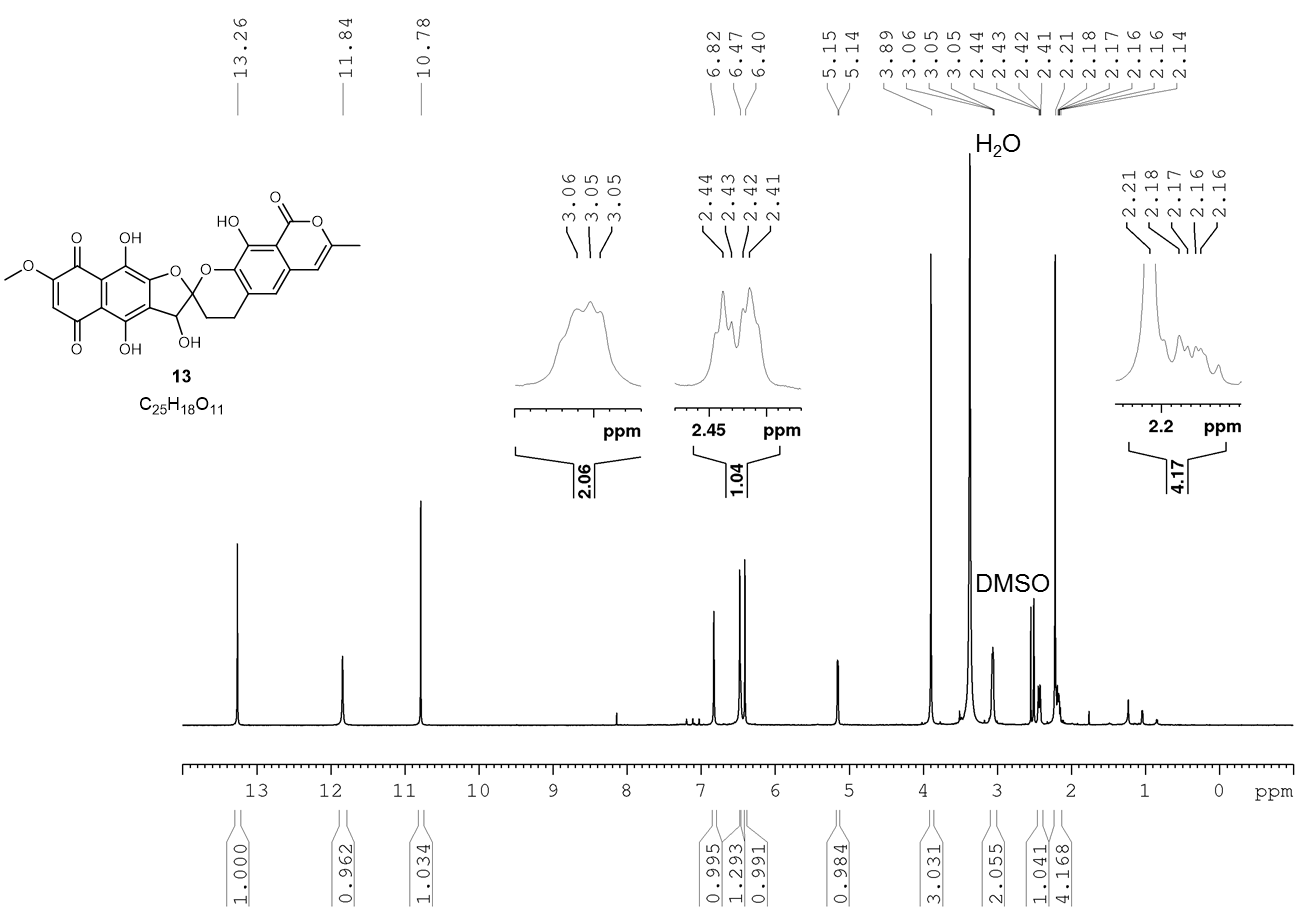


**Supplementary Figure 36**. ^1^H NMR spectrum of 7,8-dideoxygriseorhodin C (**13**) (DMSO-d_6_, 600 MHz).

**
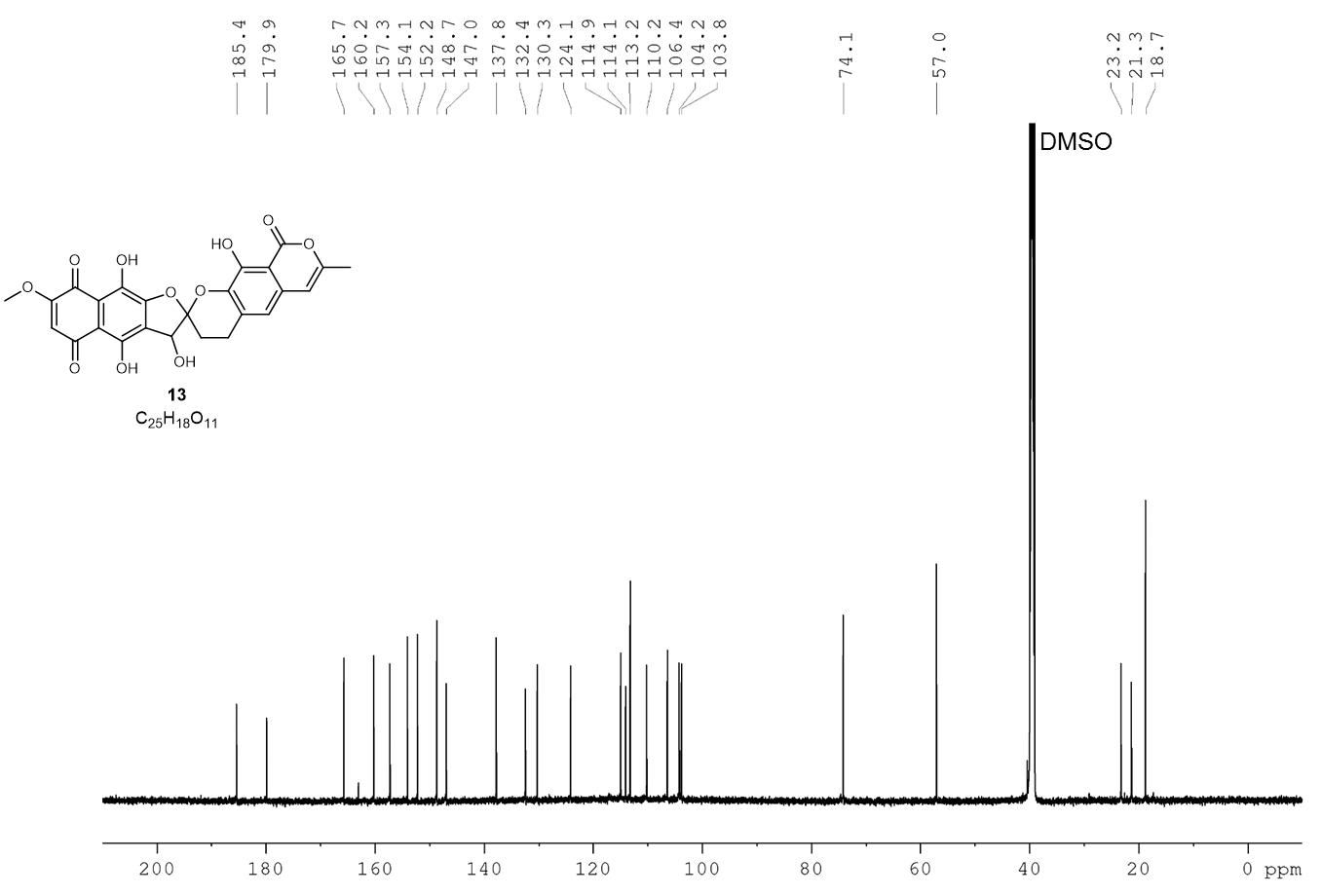
**

**Supplementary Figure 37**. ^13^C NMR spectrum of 7,8-dideoxygriseorhodin C (**13**) (DMSO-d_6_, 150 MHz).

**Supplementary Figure 38**. RP-HPLC traces at 254 nm of 7,8-dideoxy-6-oxo-griseorhodin C (**4**) produced by two different methods. **A**) Chemical oxidation of 7,8-dideoxygriseorhodin C (**13**) to **4** using Dess-Martin periodinane (DMP). **B**) Chromatogram showing traces of compound **4** enzymatically formed from **3** by GrhO5 and GrhO6. For both experiments n=3 independent replicates were analyzed and a representative example is shown.


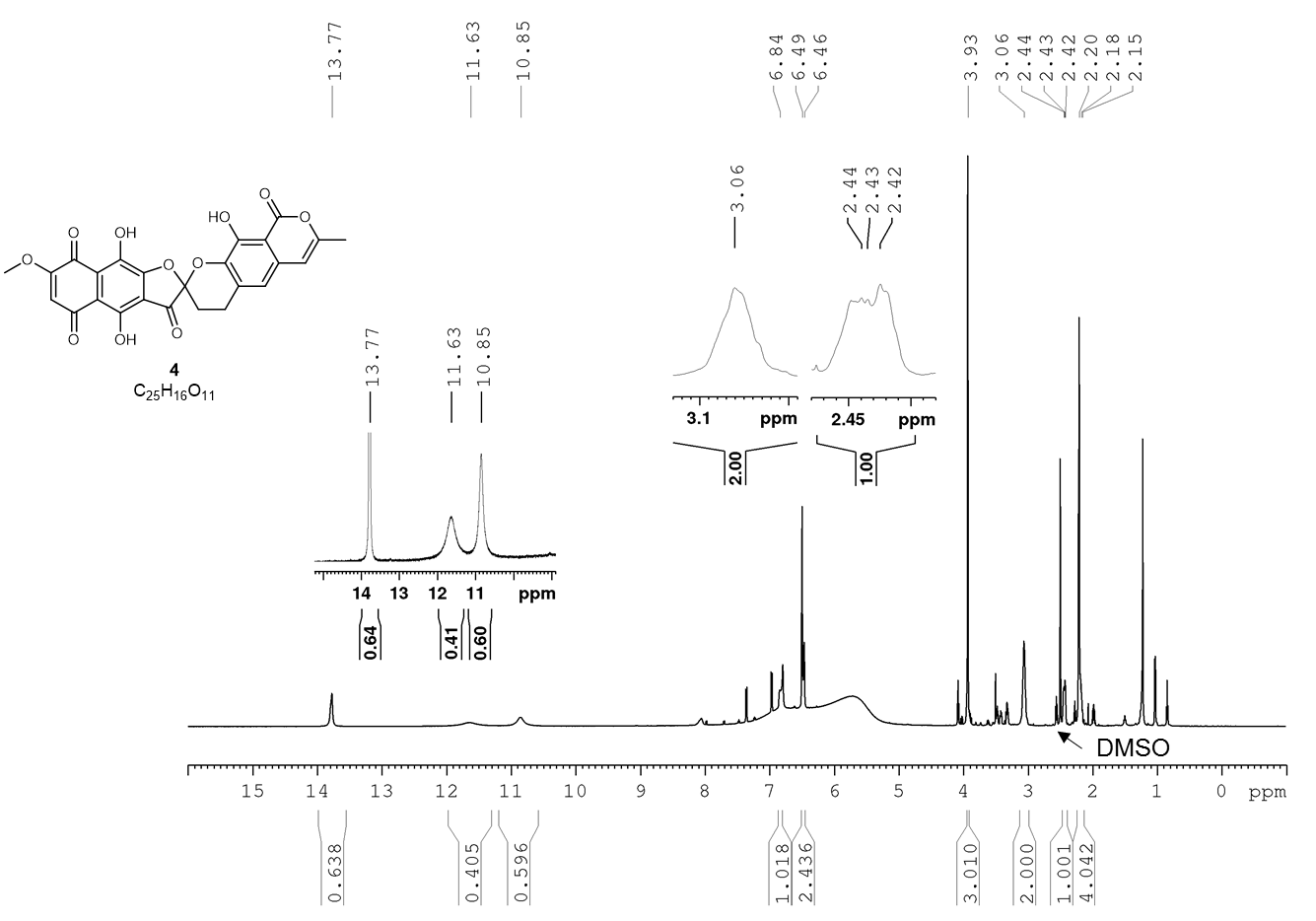


**Supplementary Figure 39**. ^1^H NMR spectrum of 7,8-dideoxy-6-oxo-griseorhodin C (**4**) (DMSO-d_6_/1% TFA-d, 600 MHz).


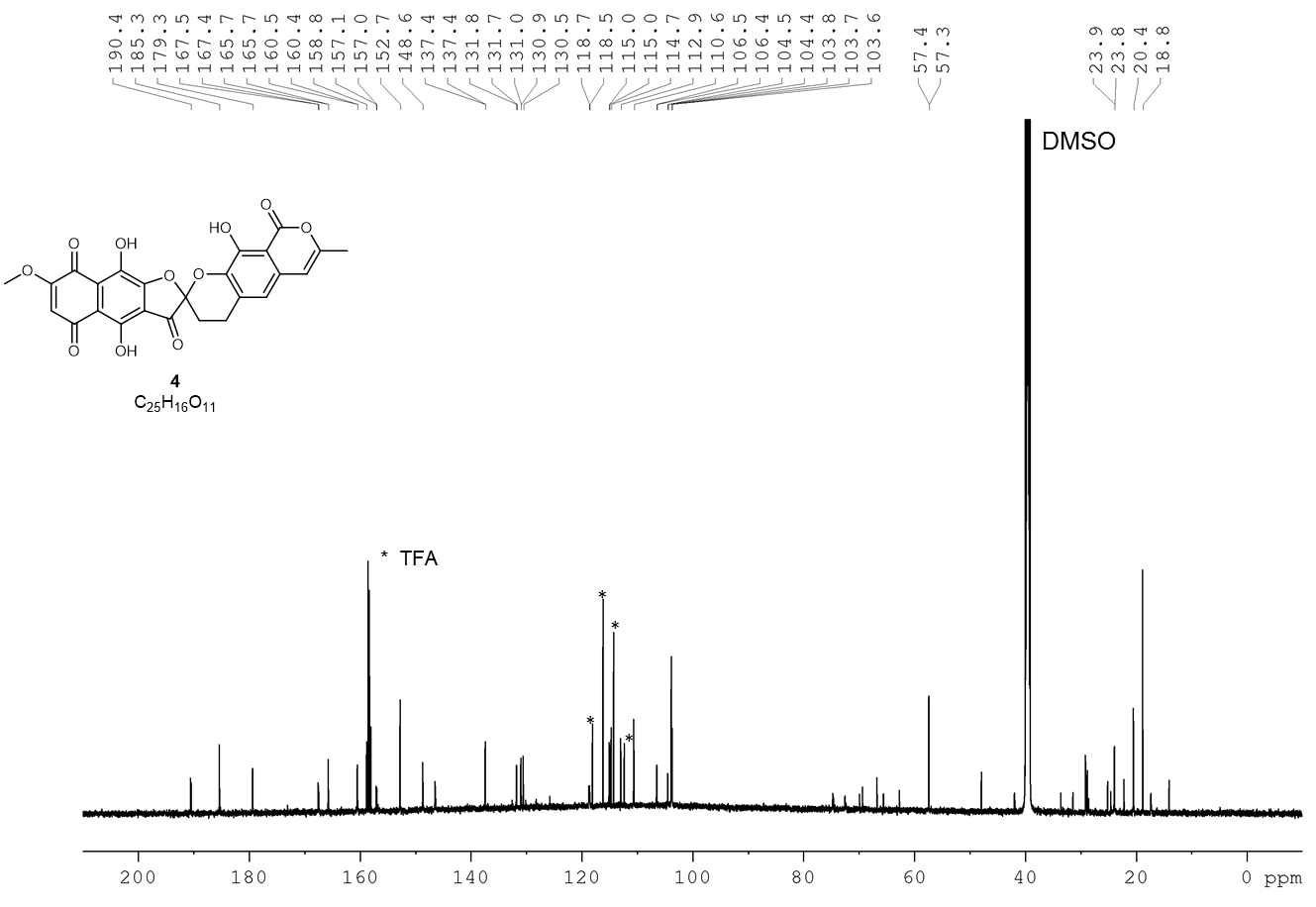


**Supplementary Figure 40**. ^13^C NMR spectrum of 7,8-dideoxy-6-oxo-griseorhodin C (**4**) (DMSO-d_6_/1% TFA-d, 151 MHz). Peaks with an asterisk belong to trifluoroacetic acid (TFA). Some peaks appear as double peak because of different isomers of compound **4**.


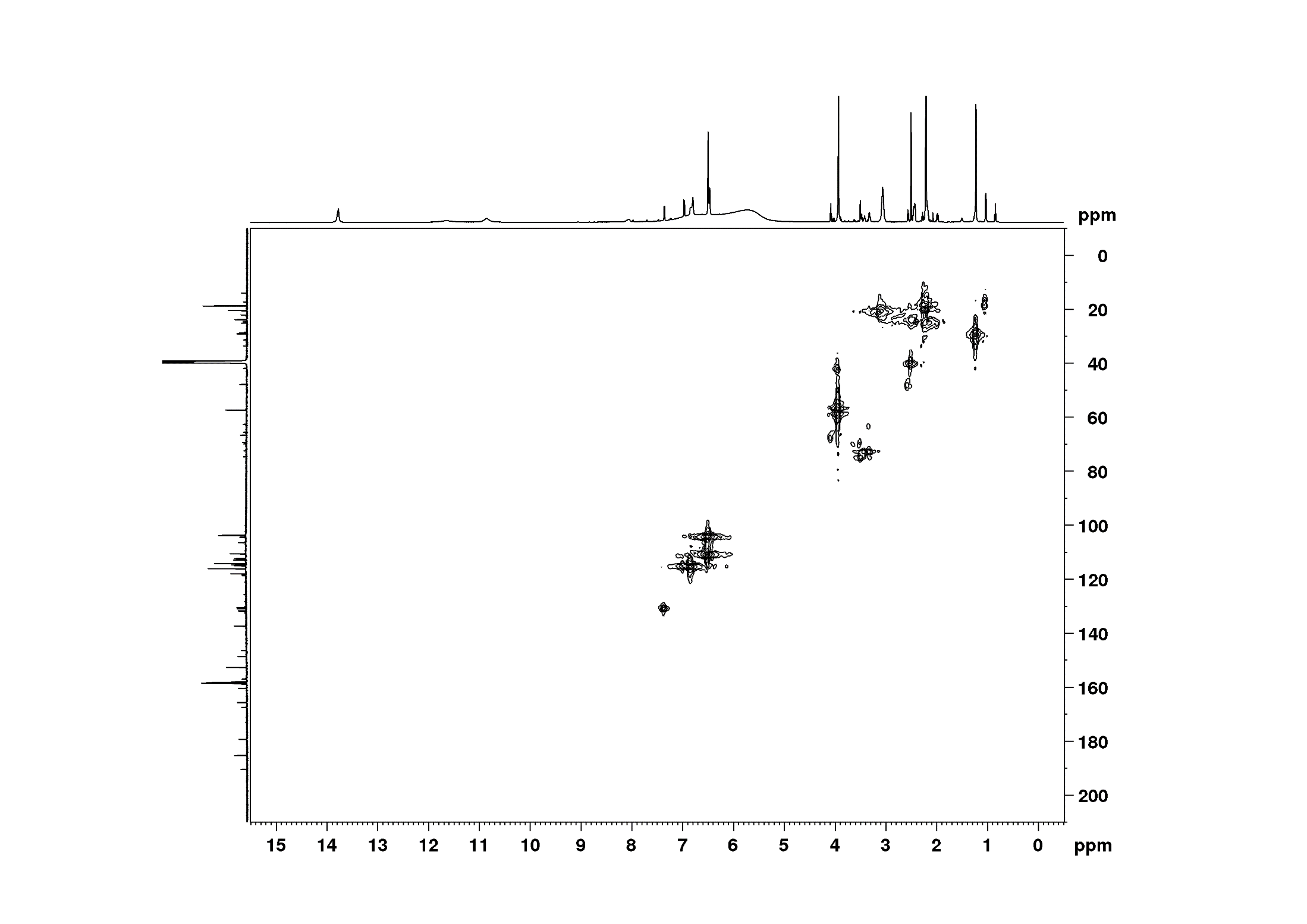


**Supplementary Figure 41**. HSQC NMR spectrum of 7,8-dideoxy-6-oxo-griseorhodin C (**4**) (DMSO-d_6_/1% TFA-d, 600 MHz).


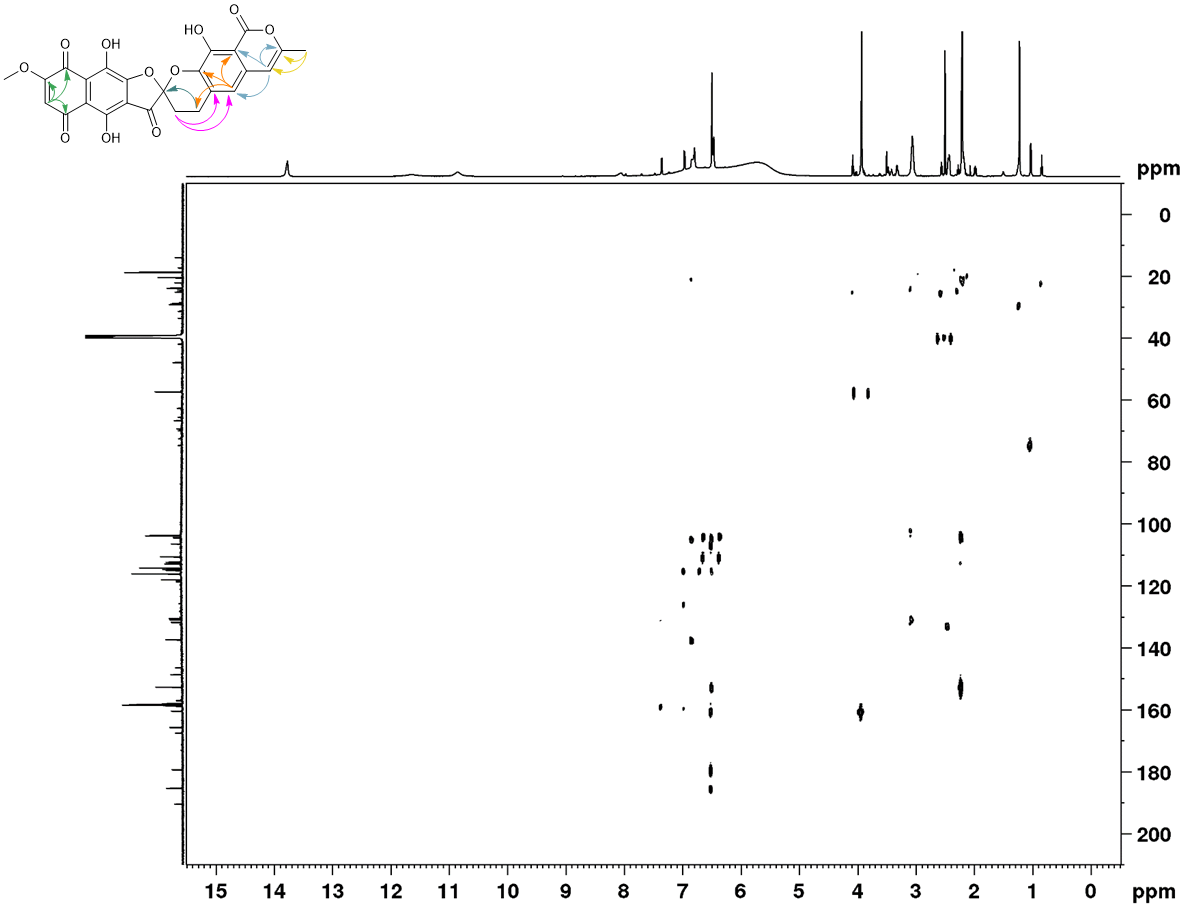


**Supplementary Figure 42**. HMBC NMR spectrum of 7,8-dideoxy-6-oxo-griseorhodin C (**4**) (DMSO-d_6_/1% TFA-d, 600 MHz). The arrows indicate the HMBC correlations of each proton.

**Supplementary Figure 43**. Conversion of compound **4** into compound **12**. **A**) Compound **4** was dissolved in acetonitrile, water was added and the solution was incubated for 60 min at pH 2 and pH 7 with temperatures from 4 – 50°C. The RP-HPLC chromatogram (254 nm) on the right side shows that **4** was readily converted to **12** at neutral pH. The left chromatogram shows that **4** is significantly more stable under acidic conditions. **B**) The reaction was performed in 70% H_2_^18^O (*v*/*v*). The incubation time with labeled water was 2 min. The results show the incorporation of a single ^18^O-atom (41%). Second (23%) and third (5%) ^18^O-atom incorporations likely result from the spontaneous exchange of carbonyls with water (e.g., the C6-ketone). The MS/MS fragmentation pattern of the labeled compound **12** is shown to the right (see Supplementary Figure 49 and Supplementary Table 6 for fragments). For all experiments n=3 independent replicates were analyzed.

**
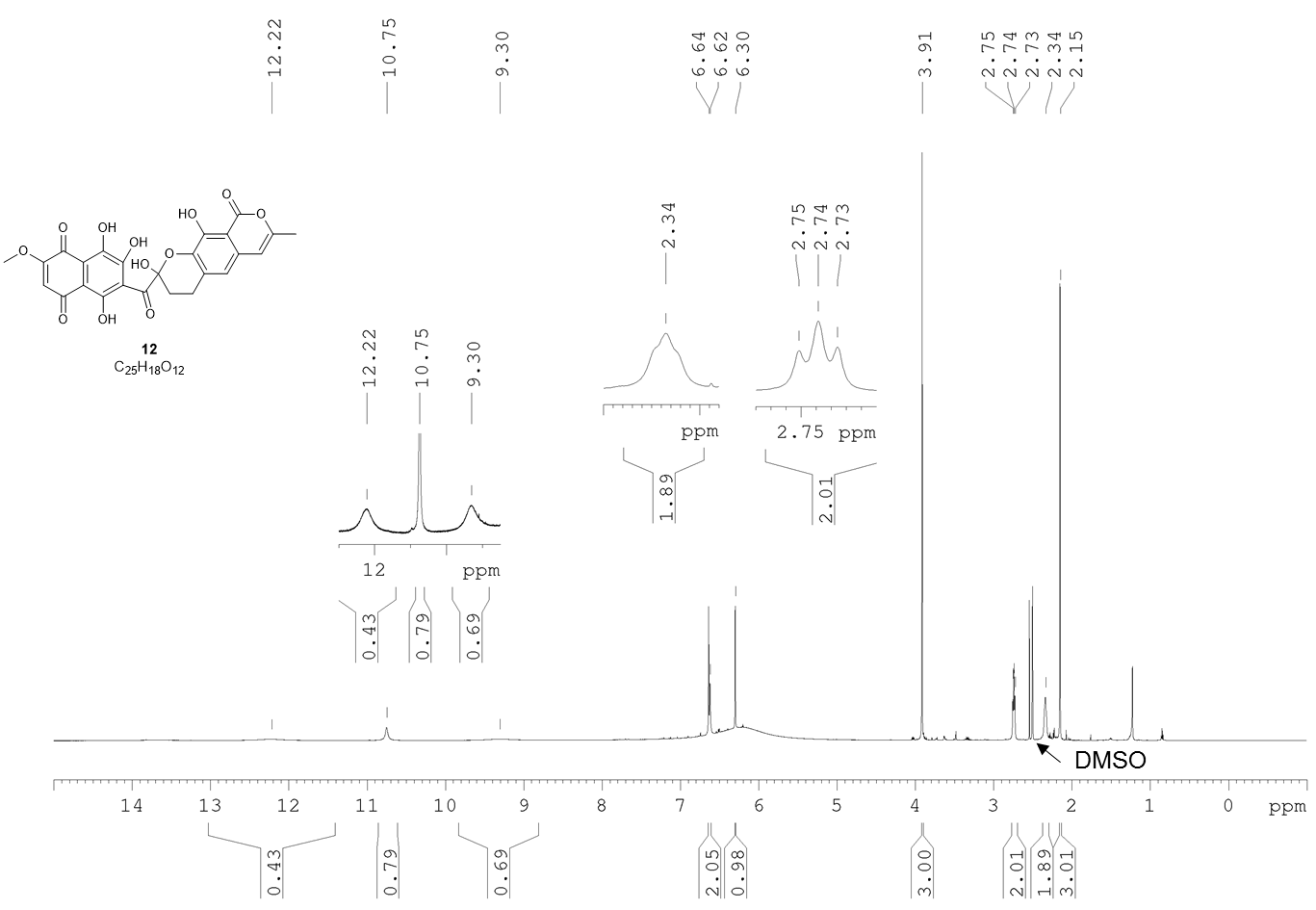
**

**Supplementary Figure 44**. ^1^H NMR spectrum of compound **12** (DMSO-d_6_/1% TFA-d, 600 MHz).

**
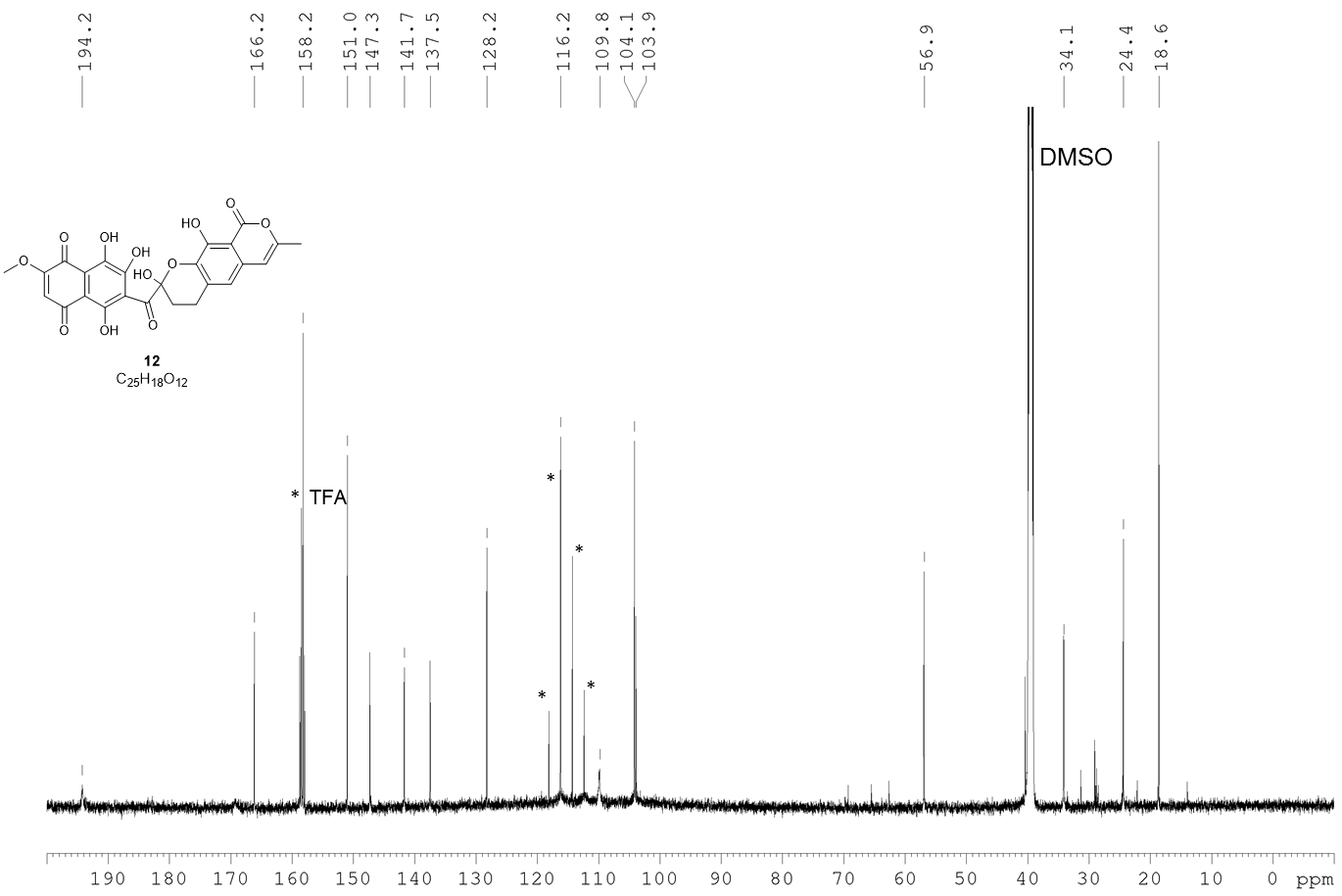
**

**Supplementary Figure 45**. ^13^C NMR spectrum of compound **12** (DMSO-d_6_/1% TFA-d, 150 MHz). The signals of 16 carbon atoms are detectable and nine carbon signals are missing. Peaks with an asterisk belong to trifluoroacetic acid (TFA).

**
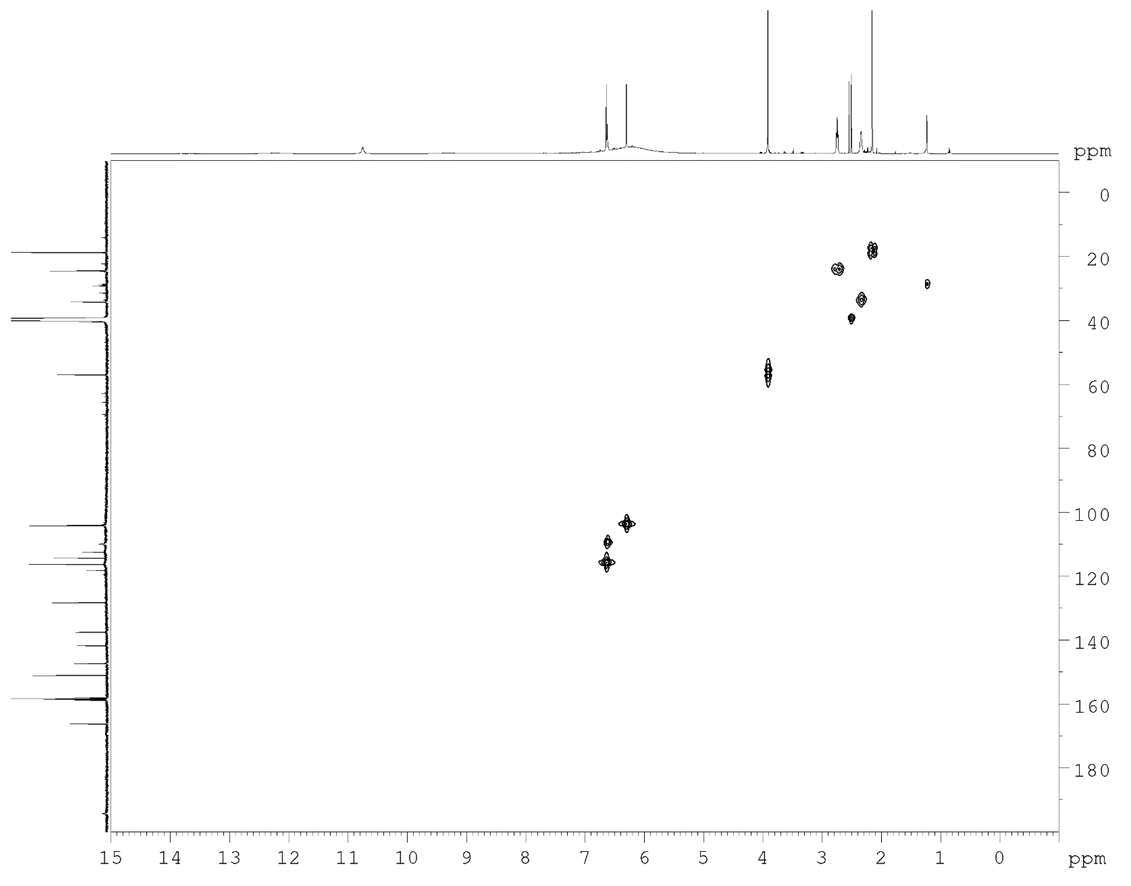
**

**Supplementary Figure 46**. HSQC NMR spectrum of compound **12** (DMSO-d_6_/1% TFA-d, 600 MHz).

**
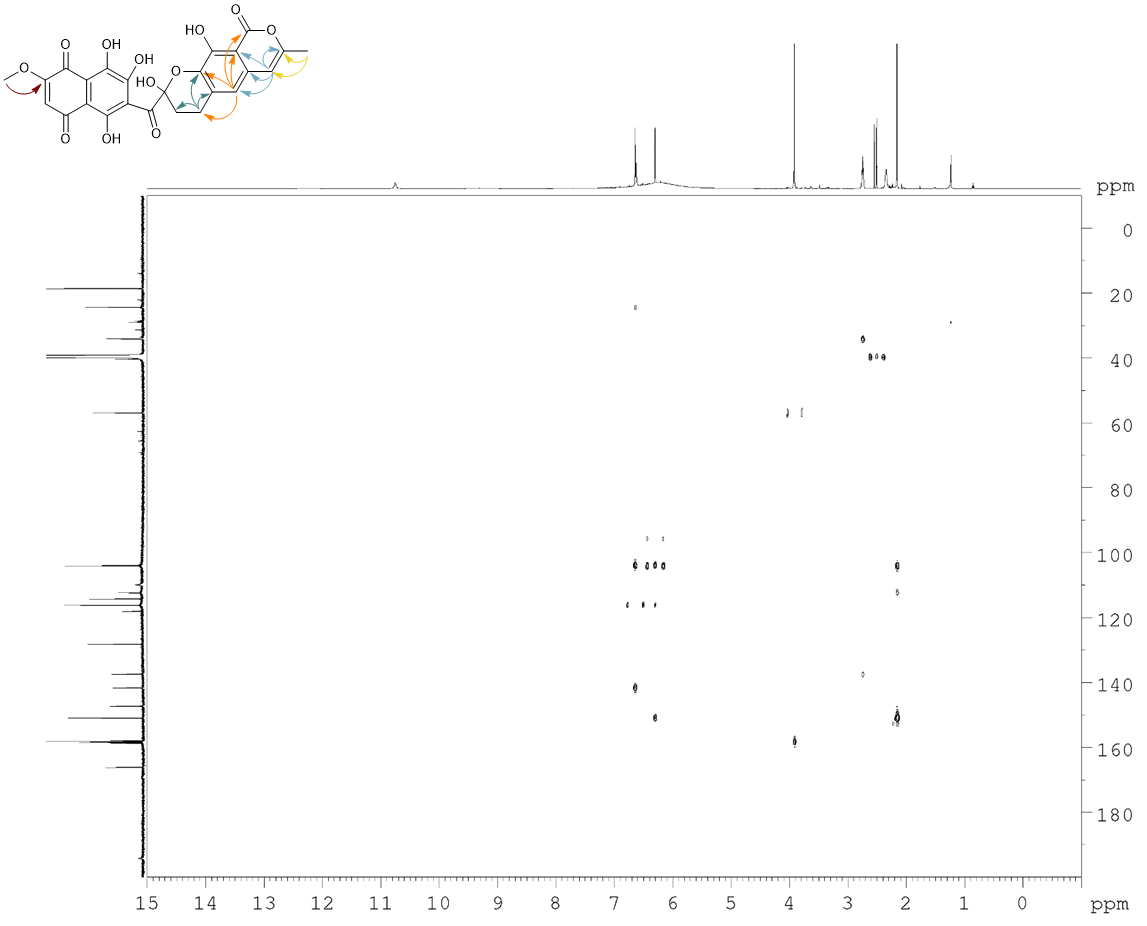
**

**Supplementary Figure 47**. HMBC NMR spectrum of compound **12** (DMSO-d_6_/1% TFA-d, 600 MHz). The arrows indicate the HMBC correlations of each proton.


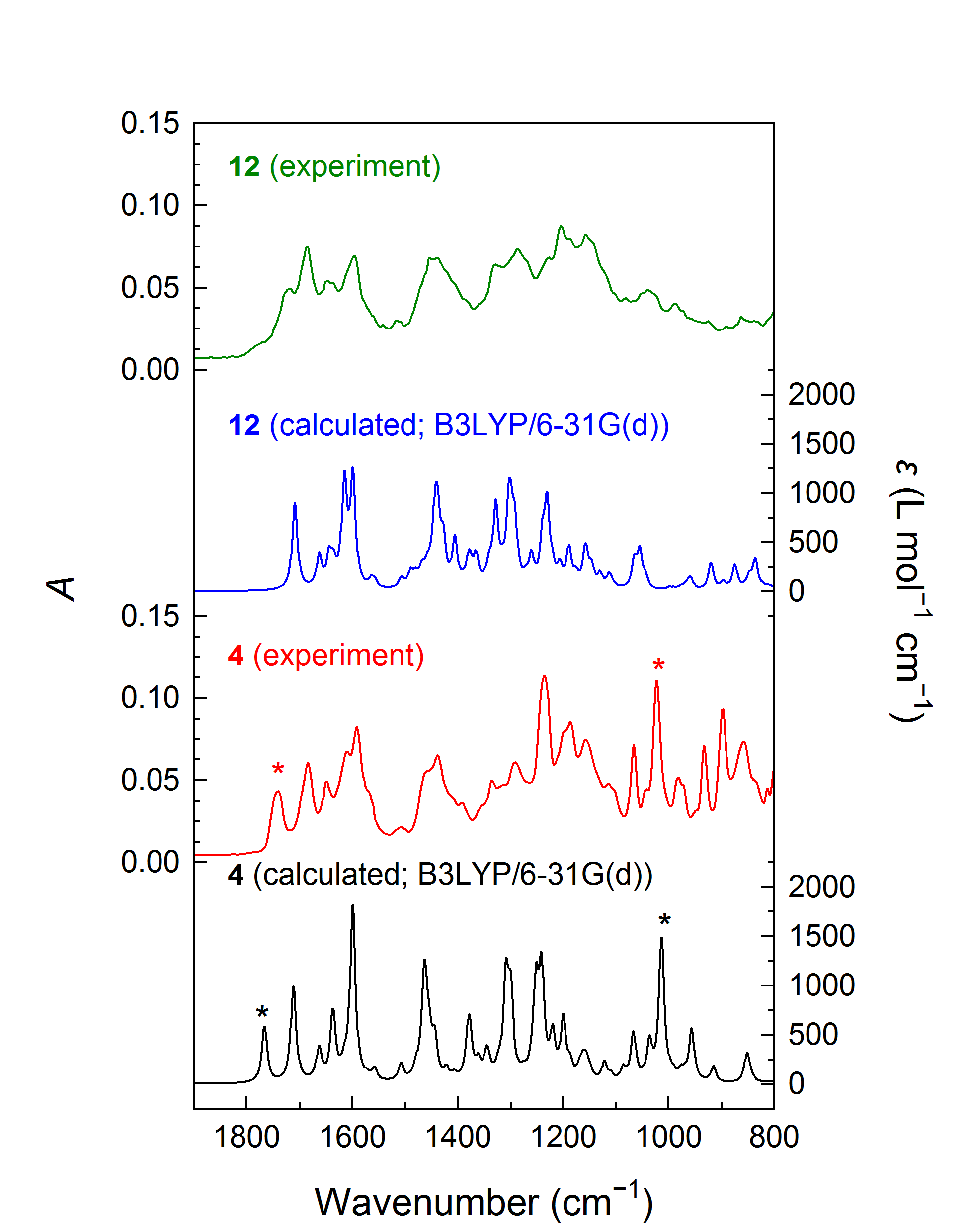


**Supplementary Figure 48**. Comparison of the carbonyl and fingerprint region of experimental solid state FTIR spectra of **4** and **12** to spectra calculated at the DFT level. The bands at 1740 cm^−1^ and 1022 cm^−1^ (marked with asterisks) represent the carbonyl stretch mode and the C–O-stretch mode of the ketone and the acetal oxygen, respectively, which are both located in the strained 5-ring. Both modes have virtually disappeared in **12** fully consistent with hydrolytic ring opening and formation of a hemiacetal at the former spiro-carbon. Note that an alternative structure of **12** with a C6-ketohydrate (instead of the hydrolytic ring-opening) cannot be ruled out. Yet, in particular the disappearing C–O-stretch mode of the acetal oxygen in **12** as well as the chemical shift observed by ^13^C-NMR for C7 (next to the spiro-C) strongly indicate an opened spiroketal (compare to the corresponding chemical shift of C14 of the ring-opened compound **9**) and thus support the structure shown in Fig. 4.

**Supplementary Figure 49.** *In vitro* assays showing the conversion of **11** into **12** by GrhO6, which, in contrast did not convert shunt product **9**. **A**) RP-HPLC chromatogram at 254 nm showing the enzymatic formation of **12** by GrhO6 and NADH or NADPH (both reductants are accepted). The control without NAD(P)H shows formation of a spontaneously formed tautomer of lenticulone (**11**). **B**) RP-HPLC analysis (chromatograms at 254 nm) showing that purified compound **9** could not be converted by GrhO6. **C**) Isotope labeling assay of compound **12** produced from **3** with GrhO5, GrhO1 and GrhO6 in presence of 97% ^18^O_2_ gas. The LC-HRMS results show the incorporation of two ^18^O-atoms. The cutout of the MS/MS fragmentation pattern of the labeled compound **12** shows the incorporation of the ^18^O-atoms in the western and eastern half of the molecule. The ^18^O-incorporation in the eastern half is effected by GrhO5 (see Supplementary Figure 27). The fragments with m/z of [M+H]^+^ = 207.046, [M+H]^+^ = 231.054 and [M+H]^+^ = 233.068 show the incorporation of the ^18^O-atom in the eastern molecule half (by GrhO5). The ^18^O-incorporation by GrhO6 takes place in the western half and is verified by the fragment with m/z of [M+H]^+^ = 291.037. For more fragments see Supplementary Table 6. For all assays, at least n=3 independent replicates were conducted and representative examples are shown.

**Supplementary Figure 50.** UV circular dichroism spectrum of 7,8-dideoxy-6-oxo-griseorhodin C (**4**) that is highly similar compared to, e.g., griseorhodin A^1,2^ and rubromycins CA1 and CA2^3^ and thus suggests the same absolute configuration.

**
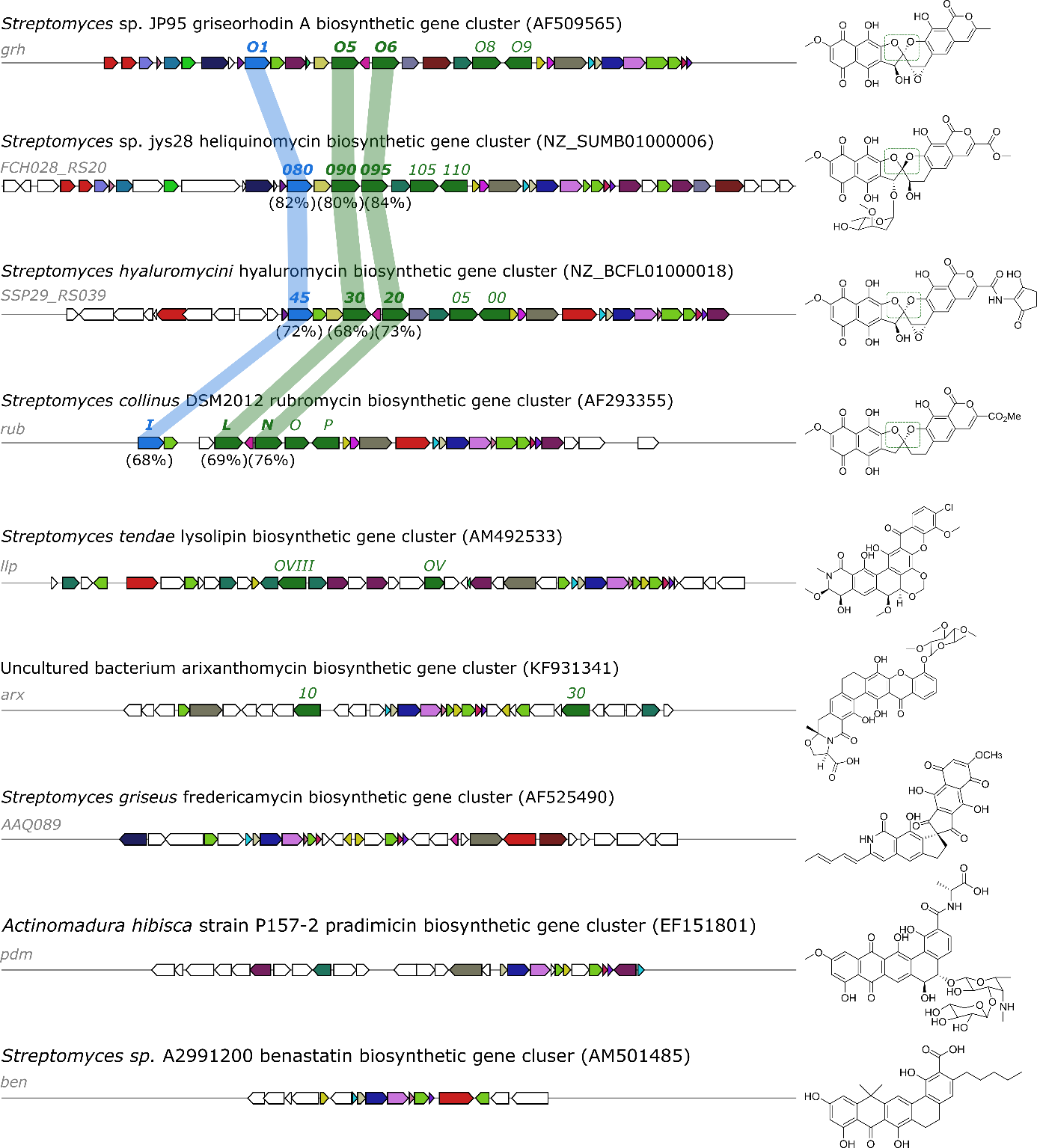
**

**Supplementary Figure 51.** Gene cluster comparison performed with MultiGeneBlast^4^. Same colors indicate high amino acid sequence similarities and predicted similar functions. The first four lines show the biosynthetic gene clusters for production of the spiroketal-containing compounds griseorhodin A (**1**, AF509565), heliquinomycin (NZ_SUMB01000006), hyaluromycin (NZ_BCFL01000018) and rubromycin (**2**, AF293355). These clusters harbor *grhO5* (green), *grhO1* (blue) and *grhO6* (green) homologues. Corresponding homologues are connected with a shadow and the amino acid sequence identities of the encoded proteins are shown (in %). The *grhO8* and *grhO9* genes also show homology to *grhO5* and *grhO6* and encode FAD-dependent monooxygenases involved in early redox tailoring steps. The biosynthetic gene clusters of the non-spiroketal compounds lysolipin (AM492533), fredericamycin A (AF525490), arixanthomycin (KF931341), benastatin A (AM501485) and pradimicin A (EF151801) lack *grhO1*, *grhO5* and *grhO6* gene candidates necessary for spiroketal formation.


**Supplementary Figure 52.** Conversion of **3** into **10** catalyzed by RubL. **A**) SDS-PAGE analysis of His_10_-tagged RubL (61 kDa) after Ni^2+^ affinity chromatography. PageRuler^TM^ plus prestained protein ladder (Thermo Fisher Scientific) was applied as reference. **B**) RP-HPLC chromatogram at 254 nm showing the conversion of collinone (**3**) into dihydrolenticulone (**10**) and shunt product (**9**) catalyzed by equimolar amounts of GrhO5 or RubL. Both reactions were quenched after 5 min and show the same intermediates and products. For these assays, at least n=3 independent replicates were performed with similar results and representative examples are shown.

**
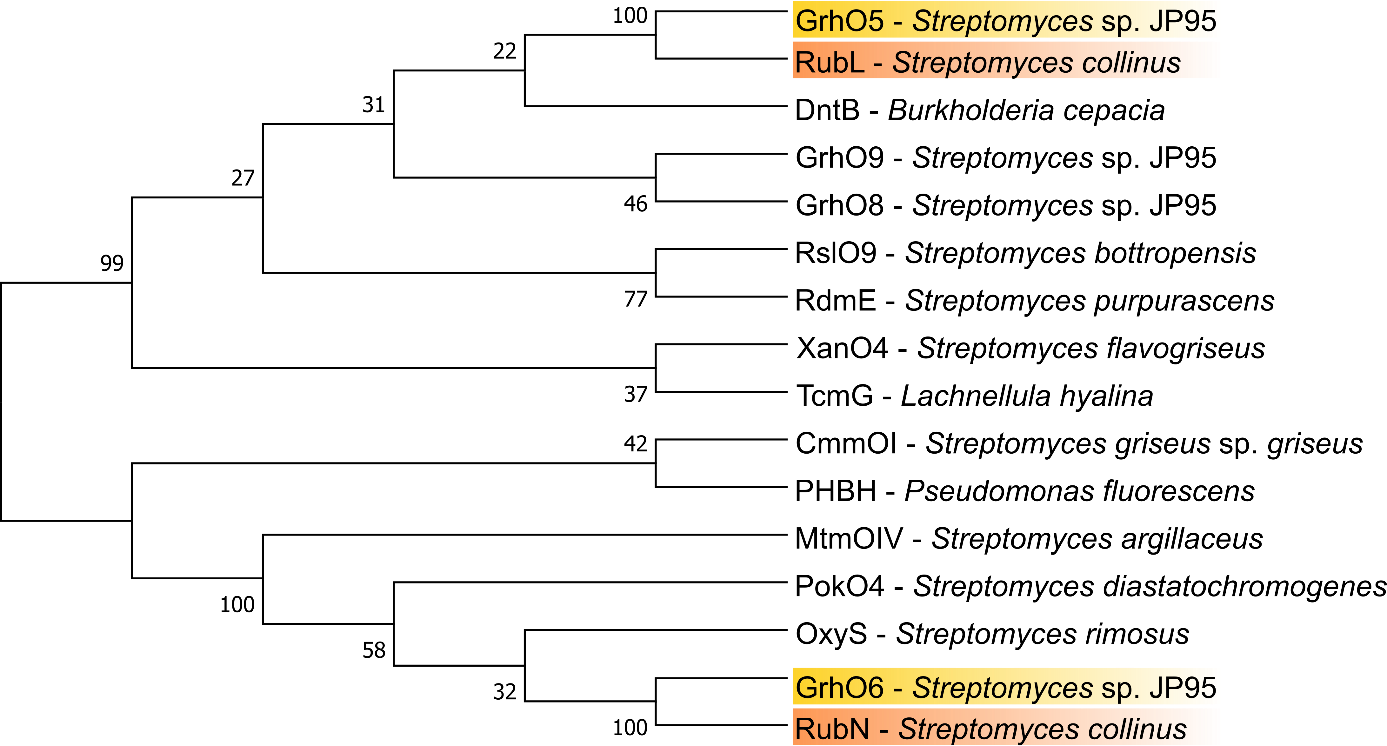
**

**Supplementary Figure 53.** Evolutionary analysis of selected members of the class A flavin monooxygenases including the archetypal *p*-hydroxybenzoate hydroxylase (PHBH) of *Pseudomonas fluorescens*. The evolutionary history was inferred by using the Maximum Likelihood method and JTT matrix-based model^5^. The bootstrap consensus tree inferred from 100 replicates is taken to represent the evolutionary history of the taxa analyzed. The percentage of replicate trees in which the associated taxa clustered together in the bootstrap test are shown next to the branches. Initial tree(s) for the heuristic search were obtained automatically by applying Neighbor-Join and BioNJ algorithms to a matrix of pairwise distances estimated using the JTT model, and then selecting the topology with superior log likelihood value. This analysis involved 16 amino acid sequences. There were a total of 644 positions in the final dataset. GrhO5 and GrhO6 are highlighted in yellow, RubL and RubN are highlighted in orange. Evolutionary analyses were conducted in MEGA X^6^. Accesssion numbers are: GrhO5, AAM33672; RubL, AAM97362; DntB, AAL50019; GrhO9, AAM33676; GrhO8, AAM33675; RslO9, AHL46732; RdmE, AAA83424; XanO4, ADE22300; TcmG, XP_031005989; CmmOI, CAE17524; PHBH, P00438; MtmOIV, CAK50794; PokO4, ACN64854; OxyS, AAZ78342; GrhO6, AAM33673; RubN, AAM97364.

**Supplementary Table 1.** HR-ESI-MS/MS fragmentation of compound **9**.

| Ion | m/z [M+H]^+^ | | Molecular formula |
| --- | --- | --- | --- |
|  | **found** | **calculated** |  |
| [M+H]^+^ | 523.089 | 523.087 | C_26_H_18_O_12_ |
| [M+H]^+^ -H_2_O | 505.077 | 505.077 | C_26_H_16_O_11_ |
| [M+H]^+^ -2H_2_O | 487.067 | 487.066 | C_26_H_14_O_10_ |
| [M+H]^+^ -H_2_O -CO | 477.082 | 477.082 | C_25_H_16_O_10_ |
| [M+H]^+^ -3H_2_O | 469.055 | 469.055 | C_26_H_12_O_9_ |
| [M+H]^+^ -2H_2_O -CO | 459.071 | 459.071 | C_25_H_14_O_9_ |
| [M+H]^+^ -2H_2_O -CO_2_ | 443.076 | 443.076 | C_25_H_14_O_8_ |
| [M+H]^+^ -2H_2_O -2CO | 431.076 | 431.076 | C_24_H_14_O_8_ |
| [M+H]^+^ -2H_2_O -CO -CO_2_ | 415.081 | 415.081 | C_24_H_14_O_7_ |
| [M+H]^+^ -2H_2_O -3CO | 403.081 | 403.081 | C_23_H_14_O_7_ |
| [M+H]^+^ -C_10_H_8_O_4_ | 331.045 | 331.045 | C_16_H_10_O_8_ |
| [M+H]^+^ -C_11_H_8_O_4_ | 319.045 | 319.045 | C_15_H_10_O_8_ |
| [M+H]^+^ -C_13_H_8_O_8_ | 231.065 | 231.065 | C_13_H_10_O_4_ |
| [M+H]^+^ -C_15_H_10_O_8_ | 205.050 | 205.050 | C_11_H_8_O_4_ |
| [M+H]^+^ -C_16_H_10_O_9_ | 177.054 | 177.055 | C_10_H_8_O_3_ |

**Supplementary Table 2.** HR-ESI-MS/MS fragmentation of compound **9** labeled with ^18^O_2_ gas.

| Ion | m/z [M+H]^+^ | | Molecular formula |
| --- | --- | --- | --- |
|  | **found** | **calculated** |  |
| [M+H]^+^ | 525.092 | 525.091 | C_26_H_18_O_11_^18^O |
| [M+H]^+^ -H_2_O^*^ | 507.082 | 507.081 | C_26_H_16_O_10_^18^O |
| [M+H]^+^ -2H_2_O | 489.070 | 489.070 | C_26_H_14_O_9_^18^O |
| [M+H]^+^ -H_2_O -CO | 479.072 | 479.086 | C_25_H_16_O_9_^18^O |
| [M+H]^+^ -2H_2_O -CO | 461.073 | 461.075 | C_25_H_14_O_8_^18^O |
| [M+H]^+^ -2H_2_O -CO_2_ | 445.054 | 445.080 | C_25_H_14_O_7_^18^O |
| [M+H]^+^ -2H_2_O -2CO | 433.076 | 433.080 | C_24_H_14_O_7_^18^O |
| [M+H]^+^ -2H_2_O -CO -CO_2_ | 417.064 | 417.086 | C_24_H_14_O_6_^18^O |
| [M+H]^+^ -2H_2_O -3CO | 405.078 | 405.086 | C_23_H_14_O_6_^18^O |
| [M+H]^+^ -C_10_H_8_O_3_^18^O | 331.042 | 331.045 | C_16_H_10_O_8_ |
| [M+H]^+^ -C_11_H_8_ O_3_^18^O | 319.047 | 319.045 | C_15_H_10_O_8_ |
| [M+H]^+^ -C_13_H_8_O_8_ | 233.065 | 233.069 | C_13_H_10_O_3_^18^O |
| [M+H]^+^ -C_15_H_10_O_8_ | 207.052 | 207.054 | C_11_H_8_O_3_^18^O |
| [M+H]^+^ -C_16_H_10_O_8_^18^O | 177.051 | 177.055 | C_10_H_8_O_3_ |

* in-source fragmentation.

**Supplementary Table 3.** HR-ESI-MS/MS fragmentation of compound **9** labeled with H_2_^18^O.

| Ion | m/z [M+H]^+^ | | Molecular formula |
| --- | --- | --- | --- |
|  | **found** | **calculated** |  |
| [M+H]^+^ | 525.089 | 525.091 | C_26_H_18_O_11_^18^O |
| [M+H]^+^ -H_2_O^*^ | 507.079 | 507.081 | C_26_H_16_O_10_^18^O |
| [M+H]^+^ -2H_2_O | 489.066 | 489.070 | C_26_H_14_O_9_^18^O |
| [M+H]^+^ -H_2_O -CO | 479.081 | 479.086 | C_25_H_16_O_9_^18^O |
| [M+H]^+^ -3H_2_O | 471.055 | 471.060 | C_26_H_12_O_8_^18^O |
| [M+H]^+^ -2H_2_O -CO | 461.069 | 461.075 | C_25_H_14_O_8_^18^O |
| [M+H]^+^ -2H_2_O -CO_2_ | 445.055 | 445.080 | C_25_H_14_O_7_^18^O |
| [M+H]^+^ -2H_2_O -2CO | 433.074 | 433.080 | C_24_H_14_O_7_^18^O |
| [M+H]^+^ -2H_2_O -CO -CO_2_ | 417.074 | 417.086 | C_24_H_14_O_6_^18^O |
| [M+H]^+^ -2H_2_O -3CO | 405.075 | 405.086 | C_23_H_14_O_6_^18^O |
| [M+H]^+^ -C_11_H_8_O_4_ | 321.042 | 321.049 | C_15_H_10_O_7_^18^O |
| [M+H]^+^ -C_13_H_8_O_7_^18^O | 231.058 | 231.065 | C_13_H_10_O_4_ |
| [M+H]^+^ -C_15_H_10_O_7_^18^O | 205.049 | 205.050 | C_11_H_8_O_4_ |
| [M+H]^+^ -C_16_H_10_O_8_^18^O | 177.053 | 177.055 | C_10_H_8_O_3_ |

* in-source fragmentation.

**Supplementary Table 4.** HR-ESI-MS/MS fragmentation of lenticulone (**11**) labeled with ^18^O_2_ gas.

| Ion | m/z [M+H]^+^ | | Molecular formula |
| --- | --- | --- | --- |
|  | **found** | **calculated** |  |
| [M+H]^+^ | 523.0773 | 523.0757 | C_26_H_16_O_11_^18^O |
| [M+H]^+^ -H_2_O | 505.0562 | 505.0651 | C_26_H_14_O_10_^18^O |
| [M+H]^+^ -2H_2_O | 487.0459 | 487.0546 | C_26_H_12_O_9_^18^O |
| [M+H]^+^ -H_2_O -CO | 477.0626 | 477.0702 | C_25_H_14_O_9_^18^O |
| [M+H]^+^ -2H_2_O -CO | 459.0525 | 459.0597 | C_25_H_12_O_8_^18^O |
| [M+H]^+^ -2H_2_O -2CO | 431.0605 | 431.0647 | C_24_H_12_O_7_^18^O |
| [M+H]^+^ -2H_2_O -3CO | 403.0665 | 403.0698 | C_23_H_12_O_6_^18^O |
| [M+H]^+^ -C_13_H_10_O_4_^18^O | 275.0156 | 275.0186 | C_13_H_6_O_7_ |
| [M+H]^+^ -C_13_H_6_O_7_ | 249.0359 | 249.0643 | C_13_H_10_O_4_^18^O |
| [M+H]^+^ -C_15_H_8_O_4_ | 207.0502 | 207.0538 | C_11_H_8_O_3_^18^O |

**Supplementary Table 5.** HR-ESI-MS/MS fragmentation of lenticulone (**11**) labeled with H_2_^18^O.

| Ion | m/z [M+H]^+^ | | Molecular formula |
| --- | --- | --- | --- |
|  | **found** | **calculated** |  |
| [M+H]^+^ | 523.0772 | 523.0757 | C_26_H_16_O_11_^18^O |
| [M+H]^+^ -H_2_O | 505.0644 | 505.0651 | C_26_H_14_O_10_^18^O |
| [M+H]^+^ -2H_2_O | 487.0534 | 487.0546 | C_26_H_12_O_9_^18^O |
| [M+H]^+^ -H_2_O -CO | 477.0694 | 477.0702 | C_25_H_14_O_9_^18^O |
| [M+H]^+^ -2H_2_O -CO | 459.0595 | 459.0597 | C_25_H_12_O_8_^18^O |
| [M+H]^+^ -2H_2_O -2CO | 431.0649 | 431.0647 | C_24_H_12_O_7_^18^O |
| [M+H]^+^ -2H_2_O -3CO | 403.0702 | 403.0698 | C_23_H_12_O_6_^18^O |
| [M+H]^+^ -C_13_H_10_O_5_ | 277.0169 | 277.0229 | C_13_H_6_O_6_^18^O |
| [M+H]^+^ -C_13_H_6_O_7_ | 249.0288 | 249.0643 | C_13_H_10_O_4_^18^O |
| [M+H]^+^ -C_15_H_8_O_3_^18^O | 205.0483 | 205.0495 | C_11_H_8_O_4_ |

**Supplementary Table 6.** HR-ESI-MS/MS fragmentation of compound **12** labeled with ^18^O_2_ gas.

| Ion | m/z [M+H]^+^ | | Molecular formula |
| --- | --- | --- | --- |
|  | **found** | **calculated** |  |
| [M+H]^+^ | 515.0943 | 515.0956 | C_25_H_18_O_10_^18^O_2_ |
| [M+H]^+^ -H_2_O^*^ | 497.0841 | 497.0850 | C_25_H_16_O_9_^18^O_2_ |
| [M+H]^+^ -2H_2_O | 479.0669 | 479.0745 | C_25_H_14_O_8_^18^O_2_ |
| [M+H]^+^ -2H_2_O -CO | 451.0696 | 451.0796 | C_24_H_14_O_7_^18^O_2_ |
| [M+H]^+^ -3H_2_O -CO | 433.0636 | 433.0690 | C_24_H_12_O_6_^18^O_2_ |
| [M+H]^+^ -C_2_H_6_O_5_ | 405.0608 | 405.0741 | C_23_H_12_O_5_^18^O_2_ |
| [M+H]^+^ -C_11_H_10_O_4_^18^O | 291.0365 | 291.0385 | C_14_H_8_O_6_^18^O |
| [M+H]^+^ -C_13_H_10_O_3_^18^O_2_ | 265.0233 | 265.0343 | C_12_H_8_O_7_ |
| [M+H]^+^ -C_12_H_8_O_7_^18^O | 233.0680 | 233.0694 | C_13_H_10_O_3_^18^O |
| [M+H]^+^ -C_12_H_10_O_7_^18^O | 231.0529 | 231.0538 | C_13_H_8_O_3_^18^O |
| [M+H]^+^ -C_14_H_10_O_7_^18^O | 207.0456 | 207.0538 | C_11_H_8_O_3_^18^O |
| [M+H]^+^ -C_15_H_10_O_7_^18^O_2_ | 177.0530 | 177.0546 | C_10_H_8_O_3_ |

* in-source fragmentation.

**Supplementary Table 7.** List of oligonucleotides to clone *grhO5*, *grhO1* and *grhO6*. Restriction sites are underlined and the respective enzymes are indicated (Thermo Fisher Scientific).

| Name | Oligonucleotide sequence | Restriction enzymes |
| --- | --- | --- |
| *grhO5* for | 5’ GTTCATATGGAGCAGCAGCGGCACACG 3’ | NdeI |
| *grhO5* rev | 5’ GTGGTCTGCAGTCAGCGCGCCAGGACGG 3’ | PstI |
| *grhO1* for | 5’ CATGCCATGGCATGCTGCGCGACGACCTCACA 3’ | NcoI |
| *grhO1* rev | 5’ CCGGAATTCTCAGGAGCCGATGCTCCGGC 3’ | EcoRI |
| *grhO6* for | 5’ CCGGAATTCGCTGGTCCCGCGCGGTAGCATGCCGGACACCAAGGGC 3' | EcoRI |
| *grhO6* rev | 5’ AAAACTGCAGTCAGCTGAAGACCGCGCGGGT 3' | PstI |

**Supplementary References**

1. Yunt, Z. *et al.* Cleavage of four carbon-carbon bonds during biosynthesis of the griseorhodin a spiroketal pharmacophore. *J. Am. Chem. Soc.* **131,** 2297–2305 (2009).

2. Ortega, H. E., Batista, J. M., Melo, W. G. P., Clardy, J. & Pupo, M. T. Absolute Configurations of Griseorhodins A and C. *Tetrahedron Lett.* **58,** 4721–4723 (2017).

3. Harunari, E., Imada, C. & Igarashi, Y. Konamycins A and B and Rubromycins CA1 and CA2, Aromatic Polyketides from the Tunicate-Derived Streptomyces hyaluromycini MB-PO13T. *J. Nat. Prod.* **82,** 1609–1615 (2019).

4. Medema, M. H., Takano, E. & Breitling, R. Detecting Sequence Homology at the Gene Cluster Level with MultiGeneBlast. *Mol. Biol. Evol.* **30,** 1218–1223 (2013).

5. Jones, D. T., Taylor, W. R. & Thornton, J. M. The rapid generation of mutation data matrices from protein sequences. *Comput. Appl. Biosci.* **8,** 275–282 (1992).

6. Kumar, S., Stecher, G., Li, M., Knyaz, C. & Tamura, K. MEGA X: Molecular Evolutionary Genetics Analysis across Computing Platforms. *Mol. Biol. Evol.* **35,** 1547–1549 (2018).
